# 7th Drug hypersensitivity meeting: part two

**DOI:** 10.1186/s13601-016-0122-y

**Published:** 2016-08-25

**Authors:** Javier Dionicio Elera, Cosmin Boteanu, Maria Aranzazu Jimenez Blanco, Rosario Gonzalez-Mendiola, Irene Carrasco García, Antonio Alvarez, Jose Julio Laguna Martinez, Jaume Martí Garrido, Carla Torán Barona, Carolina Perales Chorda, Ramón López Salgueiro, Miguel Díaz Palacios, Dolores Hernández Fernández De Rojas, Emre Ali Acar, Ayse Aktas, Aylin Türel Ermertcan, Peyker Temiz, Chien-Yio Lin, Chung-Yee Rosaline Hui, Ya-Ching Chang, Chih-Hsun Yang, Wen-Hung Chung, Fabrícia Carolino, Diana Silva, Eunice Dias De Castro, Josefina R. Cernadas, Luis Felipe Ensina, Carolina Aranda, Ines Camelo Nunes, Alex Lacerda, Ana Maria Martins, Ekaterini Goudouris, Marcia Ribeiro, José Francisco Da Silva Franco, Leandra Queiroz, Dirceu Solé, Ceyda Tunakan Dalgiç, Aytül Zerrin Sin, Fatma Düsünür Günsen, Gökten Bulut, Fatma Ömür Ardeniz, Okan Gülbahar, Emine Nihal Mete Gökmen, Ali Kokuludag, Ana M. Montoro De Francisco, Talía Mª De Vicente Jiménez, Adriana M. Mendoza Parra, Angella M. Burgos Pimentel, Amelia García Luque, Luis Amaral, Leonor Carneiro Leão, Nicole Pinto, Joana Belo, João Marques, Pedro Carreiro-Martins, Paula Leiria-Pinto, Amel Chaabane, Haifa Ben Romdhane, Nadia Ben Fredj, Zohra Chadly, Naceur A. Boughattas, Karim Aouam, Astrid P. Uyttebroek, Chris H. Bridts, Antonino Romano, Didier G. Ebo, Vito Sabato, Anabela Lopes, Joana Cosme, Rita Aguiar, Tatiana Lourenço, Maria-João Paes, Amélia Spínola-Santos, Manuel Pereira-Barbosa, Cíntia Rito Cruz, Rute Pereira Dos Reis, Elza Tomaz, Ana Paula Pires, Filipe Inácio, Filipe Benito-Garcia, Inês Mota, Magna Correia, Ângela Gaspar, Marta Chambel, Susana Piedade, Mário Morais-Almeida, Alla Nakonechna, Yurij Antipkin, Tetiana Umanets, Fernando Pineda, Francisca Arribas, Volodymyr Lapshyn, Pablo Andrés Miranda, Bautista De La Cruz Hoyos, Aranzazu Jimenez Blanco, Marta Del Pozo, Alessandra Vultaggio, Francesca Nencini, Sara Pratesi, Andrea Matucci, Enrico Maggi, Ivana Cegec, Danica Juricic Nahal, Viktorija Erdeljic Turk, Matea Radacic Aumiler, Ksenija Makar Ausperger, Iva Kraljickovic, Iveta Simic, Yukie Yamaguchi, Tomoya Watanabe, Megumi Satoh, Tomohiko Tanegashima, Kayoko Oda, Hidefumi Wada, Michiko Aihara, Jaechun Jason Lee, Jay Chol Choi, Hwa Young Lee, Rosa-Anita Rodrigues Fernandes, Emília Faria, Joana Pita, Nuno Sousa, Carmelita Ribeiro, Isabel Carrapatoso, Ana Todo Bom, Ana Rodolfo, Eunice Dias-Castro, Marina Voronova, Diana Kury Valle, Verónica Pacheco Coronel, Carolina Perales Chordá, Roselle Catherine Yu Madamba, Marta Ferrer, Maria Jose Goikoetxea, Carmen D’Amelio, Amalia Bernad, Olga Vega, Gabriel Gastaminza, Beatriz Pola Bibián, Marina Lluncor Salazar, Gemma Vilà-Nadal, Ana María Fiandor Roman, Javier Dominguez Ortega, Miguel Gonzalez Muñoz, Santiago Quirce Gancedo, Maria Rosario Cabañas Moreno, Kathrin Scherer Hofmeier, Vladyslava Barzylovych, Beatriz Pola, Marina Lluncor, Ana Fiandor, Teresa Bellón, Javier Domínguez, Santiago Quirce, Min-Suk Yang, Sun-Sin Kim, Sae-Hoon Kim, Hye-Ryun Kang, Heung-Woo Park, Sang-Heon Cho, Kyung-Up Min, Yoon-Seok Chang, Clémence Delahaye, Jenny Flabbee, Julie Waton, Olivia Bauvin, Annick Barbaud, Najah Ben Fadhel, Sandra Jerkovic Gulin, Anca Chiriac, Bárbara Kong Cardoso, Regina Viseu, Ana Moreira, Susana Cadinha, Ana Castro Neves, Patrícia Barreira, Daniela Malheiro, J. P. Moreira Da Silva, Ružica Jurakic-Toncic, Suzana Ljubojevic, Petra Turcic, Liesbeth Gilissen, Sara Huygens, An Goossens, Inmaculada Andreu, Alicia Martinez Romero, Pau Gomez Cabezas, Pedro Ayuso Parejo, Maria Del Carmen Plaza-Serón, Inmaculada Doña, Natalia Blanca-López, Carlos Flores, María Luisa Galindo, Ana Molina, James Richard Perkins, José Antonio Cornejo-García, José Augusto García-Agúndez, Elena García-Martín, Paloma Campo, María Gabriela Canto, Miguel Blanca, Rosa María Guéant-Rodríguez, Raquel Jurado-Escobar, Esther Barrionuevo, María Salas, Gabriela Canto, Jean-Louis Guéant, Toru Usui, Arun Tailor, Lee Faulkner, John Farrell, Ana Alfirevic, B. Kevin Park, Dean J. Naisbitt, Oswaldo Trelles, María Auxiliadora Guerrero, Alex Upton, Mayumi Ueta, Hiromi Sawai, Chie Sotozono, Katushi Tokunaga, Shigeru Kinoshita, Chonlaphat Sukasem, Patompong Satapornpong, Therdpong Tempark, Pawinee Rerknimitr, Kulprapat Pairayayutakul, Jettanong Klaewsongkram, N. Koomdee, T. Jantararoungtong, S. Santon, A. Puangpetch, U. Intusoma, W. Tassaneeyakul, V. Theeramoke, Elena Ramirez, Alberto Manuel Borobia, Hoi Tong, Jose Luis Castañer, Francisco José De Abajo, Violeta Régnier Galvao, Rebecca Pavlos, Elizabeth Mckinnon, Kristina Williams, Alicia Beeghly-Fadiel, Alec Redwood, Elizabeth Phillips, Mariana Castells, Elisa Boni, Marina Russello, Marina Mauro, Kok Loong Ue, Krzysztof Rutkowski, Victor Soriano Gomis, Jorge Frances Ferre, Angel Esteban Rodriguez, Vicente Cantó Reig, Javier Fernandez Sanchez, Christine Breynaert, Erna Van Hoeyveld, Rik Schrijvers, Aranzazu Jimenez Blanco, Raquel Fuentes Irigoyen, Daniel Collado, Yolanda Vida, Francisco Najera, Ezequiel Perez-Inestrosa, Pablo Mesa-Antunez, Cristobalina Mayorga, María José Torres, Line K. Tannert, Charlotte G. Mortz, Per Stahl Skov, Carsten Bindslev-Jensen, Wolfgang Pfützner, Hannah Dörnbach, Johanna Visse, Michele Rauber, Christian Möbs, Abdelbaset A. Elzagallaai, Lindsey Chow, Awatif M. Abuzgaia, Michael J. Rieder, Jason Trubiano, Emily Woolnough, Kaija Stautins, Christina Cheng, Kenichi Kato, Hiroaki Azukizawa, Takaaki Hanafusa, Ichiro Katayama, Toshiharu Fujiyama, Hideo Hashizume, Takatsune Umayahara, Taisuke Ito, Yoshiki Tokura, Mira Silar, Mihaela Zidarn, Helena Rupnik, Peter Korosec, Alec James Redwood, Kaija Strautins, Katie White, Abha Chopra, Katherine Konvinse, Shay Leary, Simon Mallal, Rosario Cabañas, Ana María Fiandor, Andrew Sullivan, Paul Whitaker, Daniel Peckham, Wei Yann Haw, Marta E. Polak, Carolann Mcguire, Michael R. Ardern-Jones, Yumi Aoyama, Tetsuo Shiohara, Sara Correia, Asli Gelincik, Semra Demir, Fatma Sen, Hamza Ugur Bozbey, Muge Olgac, Derya Unal, Raif Coskun, Bahauddin Colakoglu, Suna Buyuozturk, Esin Çatin-Aktas, Gunnur Deniz, Jose Julio Laguna, J. Dionicio, Tahia Fernandez, I. Olazabal, Maria Dolores Ruiz, Maria José Torres, Alberto Lafuente, Jorge Núñez, Tahia Diana Fernández, Francisca Palomares, Rubén Fernández, Maria Isabel Sanchez, Tahía Fernandez, Arturo Ruiz, Adriana Ariza, Amalia Bernad Alonso, Carmen D’Amelio Garófalo, Olga Vega Matute, Marta Ferrer Puga, María José Goikoetxea Lapresa, Gabriel Gastaminza Lasarte, Antonia Thinnes, Hans F. Merk, Jens Malte Baron, Martin Leverkus, Galina Balakirski, Andrew Gibson, Monday Ogese, Zaid Al-Attar, Fiazia Yaseen, Xiaoli Meng, Rozalind Jenkins, John Farrel, Khetam Alhilali, Yanni Xue, Patricia Illing, Nicole Mifsud, Heidi Fettke, Jeffrey Lai, Rebecca Ho, Patrick Kwan, Anthony Purcell, Monday O. Ogese, Catherine Betts, Paul Thomson, Mohammad Alhaidari, Neill Berry, Paul M. O’Neill, Abdulaziz Alzahrani, Marie Eliane Azoury, Lucia Fili, Rami Bechara, Noémie Scornet, Cathy Nhim, Richard Weaver, Nancy Claude, Delphine Joseph, Bernard Maillere, Paola Parronchi, Marc Pallardy, Axel Patrice Villani, Aurore Rozières, Benoît Bensaïd, Mathilde Tardieu, Floriane Albert, Virginie Mutez, Tugba Baysal, Janet Maryanski, Jean-François Nicolas, Osami Kanagawa, Marc Vocanson, Shuen-Iu Hung, Caroline J. Harrison, Rosalind E. Jenkins, Neil S. French, Maria Isabel Montañez, Tahia D. Fernandez, Angela Martin-Serrano, Maria Jose Torres, Noemi Molina, Sally Wood, Munir Pirmohamed, María Isabel Montañez, Ángela Martín-Serrano, Ezequiel Pérez-Inestrosa, Dolores Pérez-Sala, Antonio E. Guzmán, Tai-Ming Ko, Yuan-Tsong Chen, Jer-Yuarn Wu, Francisco J. Sánchez-Gómez, Juan M. González-Morena, María J. Torres, Alejandra Monroy Arreola, Jesus Agustin Badillo Corona, Silvia Mendez Flores, Judith Dominguez Cherit, Noe Valentin Duran Figueroa, Jose Luis Castrejon Flores, James Perkins, Diana Pérez-Alzate, Gador Bogas, María J Torres, Luis Mario Tubella Marti, Fernando Pineda De La Losa, Francisca Arribas Poves, Jaime Tubella Lopez, Teodora Lopez Santiago

**Affiliations:** 1 Hospital de la Cruz Roja, Madrid, Spain; 2 IIS La Fe, Valencia, Spain; 3 Department of Internal Medicine, Faculty of Medicine, Celal Bayar University, Manisa, Turkey; 4 Department of Allergy and Immunology, Faculty of Medicine, Celal Bayar University, Manisa, Turkey; 5 Department of Dermatology, Faculty of Medicine, Celal Bayar University, Manisa, Turkey; 6 Department of Pathology, Faculty of Medicine, Celal Bayar University, Manisa, Turkey; 7 Department of Dermatology, Chang Gung Memorial Hospital, Linkou, Taiwan; 8 Department of Dermatology, Drug Hypersensitivity Clinical and Research Center, Chang Gung Memorial Hospital, Linkou, Taiwan; 9 Serviço de Imunoalergologia, Centro Hospitalar de São João E.P.E., Porto, Portugal; 10 Laboratório de Imunologia, Faculdade de Medicina, Universidade do Porto, Porto, Portugal; 11 Federal University of São Paulo, São Paulo, Brazil; 12 Federal University of Rio de Janeiro, Rio De Janeiro, Brazil; 13 Pontificia Universidade Católica de Campinas, Campinas, Brazil; 14 Private Practice, Goiania, Brazil; 15 Department of Allergy and Clinical Immunology, Ege University Medical Faculty, Izmir, Turkey; 16 Hospital Central de la Defensa, IMIDEF, Madrid, Spain; 17 Centro Hospitalar de Lisboa Central, Hospital de Dona Estefânia, Lisbon, Portugal; 18 Faculty of Medicine, University hospital, University of Monastir, Monastir, Tunisia; 19 Faculty of Medicine and Health Sciences, Department of Immunology, Allergology, Rheumatology and Antwerp University Hospital, Immunology, Allergology, Rheumatology, University of Antwerp, Antwerp, Belgium; 20 Allergy Unit, Complesso Integrato Columbus, Rome, Italy; 21 Immunoallergy Department - Hospital de Santa Maria, Centro Hospitalar Lisboa Norte, Lisbon, Portugal; 22 Faculdade de Medicina de Lisboa, Lisbon, Portugal; 23 Serviço de Imunoalergologia, Hospital de São Bernardo, Setúbal, Portugal; 24 CUF Descobertas Hospital, Lisbon, Portugal; 25 Royal Liverpool and Broadgreen University Hospitals NHS Trust, Liverpool, UK; 26 Institute of Pediatrics, Obstetrics and Gynaecology, Kiev, Ukraine; 27 Diater Laboratorios, Madrid, Spain; 28 Universidad Nacional de Colombia, Cartagena, Colombia; 29 Allergy Unit, Hospital Central Cruz Roja, Madrid, Spain; 30 AOU careggi, Florence, Italy; 31 Department of Internal Medicine, Florence, Italy; 32 Ege University Medical Faculty, Izmir, Turkey; 33 University Hospital Zagreb, Zagreb, Croatia; 34 Yokohama City University Graduate School of Medicine, Yokohama, Japan; 35 Jeju National University School of Medicine, Jeju, South Korea; 36 Allergy and Clinical Immunology Department, Coimbra University Hospital Center, Coimbra, Portugal; 37 Allergy and Clinical Immunology Consult, Leiria Hospital Center, Leiria, Portugal; 38 Coimbra University Hospital Centre, Coimbra, Portugal; 39 Centro Hospitalar de São João, Porto, Portugal; 40 State Hospital 52, Moscow, Russia; 41 Clinica Universidad de Navarra, Pamplona, Spain; 42 La Paz Hospital, Madrid, Spain; 43 Allergy Unit, Department of Dermatology, University Hospital Basel, Basel, Switzerland; 44 Drug Allergy Diagnostics Centre, Institute of Paediatrics, Obstetrics and Gynaecology of NAMS, Kiev, Ukraine; 45 Allergy Department, La Paz Hospital Institute for Health Research (IdiPAZ), Madrid, Spain; 46 Allergy Department, La Paz Hospital Institute for Health Research (IdiPAZ), Consorcio Piel en RED, Madrid, Spain; 47 Seoul National University Borame Hospital, Seoul, South Korea; 48 Seoul National University Hosptial Healthcare System Gangnam Center, Seoul, South Korea; 49 Seoul National University Bundang Hospital, Seongnam, South Korea; 50 Seoul National University Hosptial, Seoul, South Korea; 51 Seoul National University College of Medicine, Seoul, South Korea; 52 Dermatology and Allergy Department, Brabois Hospital, University Hospital of Nancy, Lorraine University, 54500 Vandoeuvre-Lès-Nancy, France; 53 Department of Dermatology and Venereology, General Hospital Sibenik, Sibenik, Croatia; 54 Dermatology Department, Nicolina Medical Centre, „P. Poni“ Research Institute of Macromolecular Chemistry, Apollonia University, Iasi, Romania; 55 Hospital de S.Bernardo, Centro Hospitalar de Setúbal, Setúbal, Portugal; 56 Centro Hospitalar Vila Nova Gaia e Espinho, Vila Nova Gaia, Portugal; 57 Department of Dermatology and Venereology, School of Medicine University of Zagreb, University Hospital Center Zagreb, Zagreb, Croatia; 58 Department of Pharmacology, Faculty of Pharmacy and Biochemistry, University of Zagreb, Zagreb, Croatia; 59 University Hospitals Leuven, Leuven, Belgium; 60 CIPF, Valencia, Spain; 61 Allergy service, Infanta Leonor University Hospital, Madrid, Spain; 62 Allergy Unit, IBIMA, Regional University Hospital of Malaga, UMA, Malaga, Spain; 63 Research Laboratory, IBIMA, Regional University Hospital of Malaga, UMA, Malaga, Spain; 64 Department of Pharmacology, University of Extremadura, Cáceres, Spain; 65 Department of Biochemistry and Molecular Biology, University of Extremadura, Cáceres, Spain; 66 Research Laboratory and Allergy Unit, IBIMA, Regional University Hospital of Malaga, UMA, Malaga, Spain; 67 University of Lorraine and University Hospital Center (CHU) of Nancy, INSERM U-95Faculty of Medicine, Vandoeuvre-Les-Nancy, France; 68 University of Liverpool, Liverpool, UK; 69 Department of Computer Architecture, University of Malaga, Malaga, Spain; 70 Department of Frontier Medical Science and Technology for Ophthalmology, Kyoto Prefectural University of Medicine, Kyoto, Japan; 71 Department of Human Genetics, Graduate School of Medicine, The University of Tokyo, Tokyo, Japan; 72 Department of Ophthalmology, Kyoto Prefectural University of Medicine, Kyoto, Japan; 73 Ramathibodi Hospital, Mahidol University, Bangkok, Thailand; 74 Division of Pharmacogenomics and Personalized Medicine, Department of Pathology, Faculty of Medicine Ramathibodi Hospital, Mahidol University, Bangkok, Thailand; 75 Division of Pediatric Dermatology, Department of Pediatrics, Faculty of Medicine, Chulalongkorn University, Bangkok, Thailand; 76 Division of Dermatology, Department of Medicine, Faculty of Medicine, Chulalongkorn University, Bangkok, Thailand; 77 Raj Pracha Samasai Institute, Department of disease control, Ministry of Public Health, Nonthaburi, Thailand; 78 Division of Allergy and Clinical Immunology, Department of Medicine, Faculty of Medicine, Allergy and Clinical Immunology Research Group, Chulalongkorn University, Bangkok, Thailand; 79 Department of Pediatrics, Faculty of Medicine, Prince of Songkla University, Songkla, Thailand; 80 Department of Pharmacology, Faculty of Medicine, Khon Kaen University, Khon Kaen, Thailand; 81 Manarom Hospital, Bangkok, Thailand; 82 IdiPAZ, Madrid, Spain; 83 Hospital Universitario La Paz, IdiPAZ, Madrid, Spain; 84 Hospital Universitario La Paz, Madrid, Spain; 85 Hospital Universitario Ramón y Cajal, Madrid, Spain; 86 Hospital Universitario Príncipe de Asturias, Alcalá De Henares, Madrid, Spain; 87 Brigham and Women`s Hospital, Division of Rheumatology, Immunology and Allergy, Harvard Medical School, Boston, MA USA; 88 Institute for Immunology and Infectious Diseases, Murdoch University, Perth, Australia; 89 Vanderbilt University Medical Center, Nashville, TN USA; 90 Vanderbilt Epidemiology Center, Institute for Medicine and Public Health, Nashville, TN USA; 91 Vanderbilt University Medical Center, Vanderbilt-Ingram Cancer Center, Nashville, TN USA; 92 Hospital Sant’Anna, Como, Italy; 93 Department of Allergy, Guy’s and St Thomas’ NHS Foundation Trust, London, UK; 94 Universitary General Hospital of Alicante, Alicante, Spain; 95 Laboratory of Clinical İmmunology, Department of Microbiology and İmmunology, KU Leuven, Leuven, Belgium; 96 Laboratory Medicine, Immunology, University Hospitals, KU Leuven, Leuven, Belgium; 97 Allergy Unit, Hospital Central de la Cruz Roja, Madrid, Spain; 98 Pharmacy Service, Hospital Central de la Cruz Roja, Madrid, Spain; 99 University of Malaga, Malaga, Spain; 100 Allergy Service, Carlos Haya, Malaga, Spain; 101 Odense Research Center for Anaphylaxis, Odense C, Denmark; 102 Department of Dermatology and Allergy Center, Odense C, Denmark; 103 Odense Research Center for Anaphylaxis, Odense C/Copenhagen, Denmark; 104 Department of Dermatology and Allergology, Philipps University Marburg, Marburg, Germany; 105 Western University, London, Canada; 106 Institute of Immunology and Infectious Diseases, Murdoch University, Perth, Australia; 107 Department of Infectious Diseases, Alfred Health, Melbourne, Australia; 108 Department of Dermatology, Osaka University, Osaka, Japan; 109 Department of Dermatology, Nara Medical University, Nara, Japan; 110 Department of Dermatology, Tokyo Medical and Dental University, Tokyo, Japan; 111 Hamamatsu University School of Medicine, Hamamatsu, Japan; 112 Shimada City Municipal Hospital, Shimada, Japan; 113 University Clinic for Respiratory and Allergic Diseases, Golnik, Slovenia; 114 ARSDERMA, Ljubljana, Slovenia; 115 Servicio de Alergia, Hospital Universitario La Paz, Madrid, Spain; 116 Servicio de Farmacología Clínica, Hospital Universitario La Paz, Madrid, Spain; 117 St James’s Hospital, Leeds, UK; 118 University of Southampton, Southampton, UK; 119 Kawasaki Hospital Kawasaki Medical School, Okayama, Japan; 120 Kyorin University School of Medicine, Tokyo, Japan; 121 Centro Hospitalar Vila Nova Gaia, Vila Nova Gaia, Portugal; 122 Centro Hospitalar de Setúbal, Setúbal, Portugal; 123 Istanbul Faculty of Medicine, Istanbul University, Istanbul, Turkey; 124 Regional Hospital of Málaga-IBIMA, Málaga, Spain; 125 Research Laboratory, IBIMA, Málaga, Spain; 126 Inmunology Department, Alfonso X el Sabio University, Madrid, Spain; 127 Research Laboratory-IBIMA, Málaga, Spain; 128 Research Unit for Allergic Diseases, IBIMA-Regional University Hospital of Malaga-UMA, Málaga, Spain; 129 Allergy Unit, IBIMA-Regional University Hospital of Malaga-UMA, Málaga, Spain; 130 Regional Hospital of Málaga, Málaga, Spain; 131 Clínica Universidad de Navarra, Pamplona, Spain; 132 Department for Dermatology and Allergology, University Hospital of Aachen, Aachen, Germany; 133 MRC Centre for Drug Safety Science, Department Molecular and Clinical Pharmacology, University of Liverpool, Liverpool, UK; 134 Regional Adult Cystic Fibrosis Unit, St James’s Hospital, Leeds, UK; 135 Infection and Immunity Program, Monash Biomedicine Discovery Institute and Department of Biochemistry and Molecular Biology, Monash University, Clayton, Australia; 136 Royal Melbourne Hospital, University of Melbourne, Parkville, Australia; 137 AstraZeneca R&D, Cambridge, UK; 138 INSERM UMR99, Univ Paris-Sud, University Paris-Saclay, Châtenay-Malabry, France; 139 Department of Experimental and Clinical medicine, University of Florence, Florence, Italy; 140 UMR CNRS807, Univ Paris-Sud, University Paris-Saclay, Châtenay-Malabry, France; 141 Institut de Recherches Internationales Servier, Suresnes, France; 142 SIMOPRO, IBiTecS, CEA, Saclay, France; 143 Inserm U1111 - CIRI, Edouard Herriot Hospital, Lyon I University, Lyon, France; 144 Inserm U1111 - CIRI, Lyon I University, Lyon, France; 145 Inserm U1111 - CIRI, Lyon, France; 146 INSERM UMR 99, Université Paris-Sud, Châtenay-Malabry, Lyon, France; 147 Unité de Thérapie Cellulaire et Génique (UTCG) and URE 004 (ImCelVir), Université Nice Sophia Antipolis, Nice, France; 148 Inserm U1111 - CIRI, Lyon-Sud Hospital, Lyon I University, Lyon, France; 149 National Yang-Ming University, Taipei, Taiwan; 150 Research Laboratory, Regional University Hospital of Malaga, UMA, Malaga, Spain; 151 Allergy Unit, Regional University Hospital of Malaga, UMA, Malaga, Spain; 152 Department of Organic Chemistry, University of Malaga, IBIMA, BIONAND, Malaga, Spain; 153 Department of Organic Chemistry, Andalusian Centre for Nanomedicine and Biotechnology-BIONAND, Universidad de Malaga-IBIMA, Malaga, Spain; 154 Andalusian Centre for Nanomedicine and Biotechnology-BIONAND, Research Laboratory Carlos Haya Hospital-IBIMA, Malaga, Spain; 155 Research Laboratory Carlos Haya Hospital-IBIMA, Malaga, Spain; 156 Allergy Service, Carlos Haya Hospital, Malaga, Spain; 157 Research Laboratory, IBIMA-Regional University Hospital of Malaga, University of Malaga, Malaga, Spain; 158 BIONAND, Malaga, Spain; 159 Allergy Unit, IBIMA-Regional University Hospital of Malaga, University of Malaga, Malaga, Spain; 160 Department of Organic Chemistry, IBIMA – University of Malaga, Malaga, Spain; 161 Department of Chemical and Physical Biology, Centro de Investigaciones Biológicas, Madrid, Spain; 162 Pharmacy Unit, Regional University Hospital of Malaga, Malaga, Spain; 163 Academia Sinica, Taipei, Taiwan; 164 Department of Chemical and Physical Biology, Centro de Investigaciones Biológicas-CSIC, Madrid, Spain; 165 Department of Organic Chemistry, University of Málaga, Malaga, Spain; 166 Allergy Unit, IBIMA-Regional Hospital of Málaga, Malaga, Spain; 167 Instituto Politecnico Nacional, Mexico, Mexico; 168 Instituto Nacional de Ciencias Medicas y Nutricion, Mexico, Mexico; 169 Alergology Service. Hospital Delfos, Barcelona, Spain; 170 Immunology Department, La Paz Hospital Institute for Health Research (IdiPAZ), Consorcio Piel en RED, Madrid, Spain; 171 Research Unit and Centro de Investigación Biomédica en Red de Enfermedades Respiratorias de Enfermedades Respiratorias, Instituto de Salud Carlos III, Madrid, Spain; 172 IRCCS Oasi Maria S.S., Troina, Italy

## Abstract

Poster walk 11: miscellaneous drug hypersensitivity 2 (P92–P94, P96–P101)

P92 16 years of experience with proton pump inhibitors (PPIs)

Javier Dionicio Elera, Cosmin Boteanu, Maria Aranzazu Jimenez Blanco, Rosario Gonzalez-Mendiola, Irene Carrasco García, Antonio Alvarez, Jose Julio Laguna Martinez

P93 Allergy evaluation of quinolone induced adverse reactions

Jaume Martí Garrido, Carla Torán Barona, Carolina Perales Chorda, Ramón López Salgueiro, Miguel Díaz Palacios, Dolores Hernández Fernández De Rojas

P94 Bupropion-induced acute urticaria and angioedema, a case report

Emre Ali Acar, Ayse Aktas, Aylin Türel Ermertcan, Peyker Temiz

P96 Delayed type hypersensitivity and study of cross-reactivity between proton-pump inhibitors

Chien-Yio Lin, Chung-Yee Rosaline Hui, Ya-Ching Chang, Chih-Hsun Yang, Wen-Hung Chung

P97 Diagnostic work-up in suspected hypersensitivity to proton-pump inhibitors: looking at cross-reactivity

Fabrícia Carolino, Diana Silva, Eunice Dias De Castro, Josefina R. Cernadas

P98 Management of infusion-related hypersensitivity reactions to enzyme replacement therapy for lysosomal diseases

Luis Felipe Ensina, Carolina Aranda, Ines Camelo Nunes, Alex Lacerda, Ana Maria Martins, Ekaterini Goudouris, Marcia Ribeiro, José Francisco Da Silva Franco, Leandra Queiroz, Dirceu Solé

P99 Management of insulin allergy with continuous subcutaneous insulin infusion

Ceyda Tunakan Dalgiç, Aytül Zerrin Sin, Fatma Düsünür Günsen, Gökten Bulut, Fatma Ömür Ardeniz, Okan Gülbahar, Emine Nihal Mete Gökmen, Ali Kokuludag

P100 Off-label use of icatibant for management of serious angioedema associated with angiotensin inhibitors

Ana M. Montoro De Francisco, Talía Mª De Vicente Jiménez, Adriana M. Mendoza Parra, Angella M. Burgos Pimentel, Amelia García Luque

P101 Thiocolchicoside anaphylaxis: an unusual suspect?

Luis Amaral, Fabricia Carolino, Leonor Carneiro Leão, Eunice Castro, Josefina Cernadas

Poster walk 12: betalactam hypersensitivity (P102–P111)

P102 A curious delayed reading: a case report of a β-lactam allergy in a child

Nicole Pinto, Joana Belo, João Marques, Pedro Carreiro-Martins, Paula Leiria-Pinto

P103 Betalactam-induced hypersensitivity: a 10-years’ experience

Amel Chaabane, Haifa Ben Romdhane, Nadia Ben Fredj, Zohra Chadly, Naceur A. Boughattas, Karim Aouam

P104 Cefazolin hypersensitivity: towards optimized diagnosis

Astrid P. Uyttebroek, Chris H. Bridts, Antonino Romano, Didier G. Ebo, Vito Sabato

P105 Clavulanic acid allergy: two cases report

Anabela Lopes, Joana Cosme, Rita Aguiar, Tatiana Lourenço, Maria-João Paes, Amélia Spínola-Santos, Manuel Pereira-Barbosa

P106 Diagnosis of betalactam allergy in an allergy department

Cíntia Rito Cruz, Rute Pereira Dos Reis, Elza Tomaz, Ana Paula Pires, Filipe Inácio

P107 Diagnostic work-up of 410 patients with suspicion of betalactam antibiotic hypersensitivity

Filipe Benito-Garcia, Inês Mota, Magna Correia, Ângela Gaspar, Marta Chambel, Susana Piedade, Mário Morais-Almeida

P108 Immediate selective hypersensitivity reactions to clavulanic acid

Alla Nakonechna, Yurij Antipkin, Tetiana Umanets, Fernando Pineda, Francisca Arribas, Volodymyr Lapshyn

P109 Prevalence and incidence of penicillin hypersensitivity reactions in Colombia

Pablo Andrés Miranda, Bautista De La Cruz Hoyos

P110 Selective sensitization to amoxicilin and clavulanic acid

Jose Julio Laguna Martinez, Aranzazu Jimenez Blanco, Javier Dionicio Elera, Cosmin Boteanu, Rosario Gonzalez-Mendiola, Marta Del Pozo

P111 Infliximab-specific T cells are detectable also in treated patients who have not developed anti-drug antibodies

Alessandra Vultaggio, Francesca Nencini, Sara Pratesi, Andrea Matucci, Enrico Maggi

Poster walk 13: biologicals, local anesthetics, others (P112–P118)

P112 A case report of allergic immediate systemic reaction to adalimumab and certolizumab

Ceyda Tunakan Dalgiç, Fatma Düsünür Günsen, Gökten Bulut, Fatma Ömür Ardeniz, Okan Gülbahar, Emine Nihal Mete Gökmen, Aytül Zerrin Sin, Ali Kokuludag

P113 Allergy to local anesthetics: negative predictive value of skin tests

Ivana Cegec, Danica Juricic Nahal, Viktorija Erdeljic Turk, Matea Radacic Aumiler, Ksenija Makar Ausperger, Iva Kraljickovic, Iveta Simic

P114 Cutaneous adverse reactions of molecular targeted agents: a retrospective analysis in 150 patients in our department

Yukie Yamaguchi, Tomoya Watanabe, Megumi Satoh, Tomohiko Tanegashima, Kayoko Oda, Hidefumi Wada, Michiko Aihara

P115 Generalized paralysis induced by local lidocaine injection

Jaechun Jason Lee, Jay Chol Choi, Hwa Young Lee

P116 Hypersensitivity to local anaesthetics: a 10 year review

Rosa-Anita Rodrigues Fernandes, Emília Faria, Joana Pita, Nuno Sousa, Carmelita Ribeiro, Isabel Carrapatoso, Ana Todo Bom

P117 Local anaesthetics: a rare culprit in hypersensitivity reactions

Ana Rodolfo, Eunice Dias-Castro, Josefina Cernadas

P118 Stevens–Johnson syndrome in clinical practice: a variant of clinical course

Marina Voronova

Poster walk 14: RCM (P119–P128)

P119 13 cases of severe anaphylactic reactions due to radiocontrast media

Jaume Martí Garrido, Ramon Lopez Salgueiro, Diana Kury Valle, Verónica Pacheco Coronel, Carolina Perales Chordá, Dolores Hernandez Fernandez De Rojas

P120 Anaphylactic shock after administration of iodinated contrast medium during cardiac catheterization

Roselle Catherine Yu Madamba, Marta Ferrer, Maria Jose Goikoetxea, Carmen D’Amelio, Amalia Bernad, Olga Vega, Gabriel Gastaminza

P121 Anaphylactic shock and cardiac arrest induced by gadolinium-based contrast agents

Beatriz Pola Bibián, Marina Lluncor Salazar, Gemma Vilà Nadal, Ana María Fiandor Roman, Javier Dominguez Ortega, Miguel Gonzalez Muñoz, Santiago Quirce Gancedo, Maria Rosario Cabañas Moreno

P122 Anaphylaxis to gadobenate and cross-reactivity to other gadolinium-based contrast agents in two patients

Kathrin Scherer Hofmeier

P123 Anaphylaxis to glatiramer acetate in a patient with multiple sclerosis

Fabrícia Carolino, Vladyslava Barzylovych, Josefina R. Cernadas

P124 Delayed hypersensitivity reaction to radiocontrast media

Fabrícia Carolino, Diana Silva, Leonor Leão, Josefina R. Cernadas

P125 Drug reaction with eosinophilia and systemic symptoms induced by iodixanol

Gemma Vilà-Nadal, Beatriz Pola, Marina Lluncor, Ana Fiandor, Teresa Bellón, Javier Domínguez, Santiago Quirce

P126 Electronic consultation support system for radiocontrast media hypersensitivity changes clinician’s behavior

Min-Suk Yang, Sun-Sin Kim, Sae-Hoon Kim, Hye-Ryun Kang, Heung-Woo Park, Sang-Heon Cho, Kyung-Up Min, Yoon-Seok Chang

P127 Hypersensitivity reactions to iodinated contrast media: skin testing and follow-up

Danica Juricic Nahal, Ivana Cegec, Viktorija Erdeljic Turk, Iva Kraljickovic, Matea Radacic Aumiler, Ksenija Makar Ausperger, Iveta Simic

P128 Would iodine allergy exist?

Clémence Delahaye, Jenny Flabbee, Julie Waton, Olivia Bauvin, Annick Barbaud

Poster walk 15: MPE/type 4 (P129–P137)

P129 Delayed hypersensitivity cutaneous reactions: a case/control study from a tunisian database

Karim Aouam, Najah Ben Fadhel, Zohra Chadly, Nadia Ben Fredj, Naceur A. Boughattas, Amel Chaabane

P130 Delayed hypersensitivity reactions to cephalosporins: a review of seven cases

Joana Cosme, Anabela Lopes, Amélia Spínola-Santos, Manuel Pereira-Barbosa

P131 Diclofenac induced allergic contact dermatitis: case series of four patients

Sandra Jerkovic Gulin, Anca Chiriac

P132 Late-onset maculopapular rash to irbesartan

Bárbara Kong Cardoso, Elza Tomaz, Regina Viseu, Filipe Inácio

P133 Nonimmediate hypersensitivity reactions to betalactams: a retrospective analysis

Ana Moreira, Susana Cadinha, Ana Castro Neves, Patricia Barreira, Daniela Malheiro, J. P. Moreira Da Silva

P134 Occupational airborne contact dermatitis to omeprazole

Ružica Jurakic-Toncic, Suzana Ljubojevic, Petra Turcic

P135 Ornidazole-induced fixed drug eruption confirmed by positive patch test on a residual pigmented lesion

Liesbeth Gilissen, Sara Huygens, An Goossens

P136 Repeated delayed reaction induced by amoxicillin and amoxicillin clavulanate

Inmaculada Andreu, Ramon Lopez-Salgueiro, Alicia Martinez Romero, Pau Gomez Cabezas

P137 Systemic photosensitivity from fenofibrate in a patient photo-sensitized to ketoprofen

Liesbeth Gilissen, An Goossens

Poster walk 16: HLA genetics (P138–P146)

P138 A copy number variation in ALOX5 and PTGER1 is associated with nonsteroidal anti-inflammatory drugs induced urticaria and/or angioedema

Pedro Ayuso Parejo, Maria Del Carmen Plaza-Serón, Inmaculada Doña, Natalia Blanca López, Carlos Flores, Luisa Galindo, Ana Molina, James Richard Perkins, Jose Antonio Cornejo-García, José Augusto García-Agúndez, Elena García-Martín, Paloma Campo, María Gabriela Canto, Miguel Blanca

P139 Association of galectin-3 (LGALS3) single nucleotide polymorphisms with non-steroidal anti-inflammatory drugs-induced urticaria/angioedema

José Antonio Cornejo-Garcia, Inmaculada Doña, Rosa María Guéant-Rodríguez, Natalia Blanca-López, María Carmen Plaza-Serón, Raquel Jurado-Escobar, Esther Barrionuevo, María Salas, María Luisa Galindo, Gabriela Canto, Miguel Blanca, Jean-Louis Guéant

P140 Detection of T cell responses to ticlopidine using peripheral blood mononuclear cells from HLA-A*33:03+ healthy donors

Toru Usui, Arun Tailor, Lee Faulkner, John Farrell, Ana Alfirevic, B. Kevin Park, Dean J. Naisbitt

P141 Epistasis approaches to identify novel genes potentially involved in NSAIDs hypersensitivity

James Richard Perkins, Jose Antonio Cornejo García, Oswaldo Trelles, Inmaculada Doña, Esther Barrionuevo, María Salas, María Auxiliadora Guerrero, Miguel Blanca, Alex Upton

P142 Genetic predisposition of cold medicine related SJS/TEN with severe ocular complications

Mayumi Ueta, Hiromi Sawai, Chie Sotozono, Katushi Tokunaga, Shigeru Kinoshita

P143 HLA-B*13:01 and dapsone induced hypersensitivity in Thai population

Chonlaphat Chonlaphat Sukasem, Patompong Satapornpong, Therdpong Tempark, Pawinee Rerknimitr, Kulprapat Pairayayutakul, Jettanong Klaewsongkram

P144 HLA-B*15:02 alleles and lamotrigine-induced cutaneous adverse drug reactions in Thai

Chonlaphat Sukasem, N. Koomdee, T. Jantararoungtong, S. Santon, A. Puangpetch, U. Intusoma, W. Tassaneeyakul, V. Theeramoke

P145 HLA-B*38:01 and HLA-A*24:02 allele frequencies in Spanish patients with lamotrigine-induced SCARs

Teresa Bellón, Elena Ramirez, Alberto Manuel Borobia, Hoi Tong, Jose Luis Castañer, Francisco José De Abajo

P146 Overrepresentation of a class II HLA haplotype in severe hypersensitivity type I reactions to carboplatin

Violeta Régnier Galvao, Rebecca Pavlos, Elizabeth Mckinnon, Kristina Williams, Alicia Beeghly-Fadiel, Alec Redwood, Elizabeth Phillips, Mariana Castells

Poster walk 17: in vivo diagnosis + sIgE (P147–P154)

P147 Absence of specific Ig-e against beta-lactams 9 months after an allergic reaction to amoxicillin-clavulanic acid

Elisa Boni, Marina Russello, Marina Mauro

P148 Drug provocation tests in suspected opioid allergy

Kok Loong Ue, Krzysztof Rutkowski

P149 Improvement to the specific IgE cut-off in the assess of β-lactamic allergy

Victor Soriano Gomis, Jorge Frances Ferre, Angel Esteban Rodriguez, Vicente Cantó Reig, Javier Fernandez Sanchez

P150 Initial false negative specific IgE to gelatin in a patient with gelatin-induced anaphylaxis

Christine Breynaert, Erna Van Hoeyveld, Rik Schrijvers

P151 Inmediate reactions to beta-lactam antibiotics: pattern of skin test response over the time

Jose Julio Laguna Martinez, Rosario Gonzalez Mendiola, Javier Dionicio Elera, Cosmin Boteanu, Aranzazu Jimenez Blanco, Marta Del Pozo, Raquel Fuentes Irigoyen

P152 New fluorescent dendrimeric antigens for the evaluation of dendritic cell maturation as a test to detect allergy reactions to amoxicillin

Daniel Collado, Yolanda Vida, Francisco Najera, Ezequiel Perez-Inestrosa, Pablo Mesa-Antunez, Cristobalina Mayorga, María José Torres, Miguel Blanca

P153 Positive skin test or positive specific IgE to penicillin does not predict penicillin allergy

Line K. Tannert, Charlotte G. Mortz, Per Stahl Skov, Carsten Bindslev-Jensen

P154 Significance of skin testing and in vitro-analysis of neuromuscular blocking agents in diagnosis of perioperative drug hypersensitivity: evaluation of a negative control population

Wolfgang Pfützner, Hannah Dörnbach, Johanna Visse, Michele Rauber, Christian Möbs

Poster walk 18: in vitro/ex vivo (P155–P158, P160–P164)

P155 Diagnostic value of the lymphocyte toxicity assay (LTA) and the in vitro platelet toxicity assay (IPTA) for β-lactam allergy

Abdelbaset A. Elzagallaai, Lindsey Chow, Awatif M. Abuzgaia, Michael J. Rieder

P156 Enzyme linked immunospot assay used in the diagnosis of severe cutaneous adverse reactions to antimicrobials

Alec Redwood, Jason Trubiano, Rebecca Pavlos, Emily Woolnough, Kaija Stautins, Christina Cheng, Elizabeth Phillips

P157 Evaluation of in vitro diagnostic methods for identifying the culprit drugs in drug hypersensitivity

Kenichi Kato, Hiroaki Azukizawa, Takaaki Hanafusa, Ichiro Katayama

P158 Ex-vivo expanded skin-infiltrating T cells from severe drug eruptions are reactive with causative drugs: a possible novel method for determination of causative drugs

Toshiharu Fujiyama, Hideo Hashizume, Takatsune Umayahara, Taisuke Ito, Yoshiki Tokura

P160 In vitro release of IL-2, IL-5 and IL-13 in diagnosis of patients with delayed-type nickel hypersensitivity

Mira Silar, Mihaela Zidarn, Helena Rupnik, Peter Korosec

P161 Single cell analysis of drug responsive T cells; identification of candidate drug reactive T cell receptors in abacavir and carbamazepine hypersensitivity

Alec James Redwood, Kaija Strautins, Katie White, Abha Chopra, Katherine Konvinse, Shay Leary, Rebecca Pavlos, Simon Mallal, Elizabeth Phillips

P162 Specificity and sensitivity of LTT in DRESS: analysis of agreement with the Spanish pharmacovigilance system probability algorithm

Rosario Cabañas, Elena Ramirez, Ana María Fiandor, Teresa Bellón

P163 The role of interleukin-22 in β-lactam hypersensitivity

Andrew Sullivan, Paul Whitaker, Daniel Peckham, B. Kevin Park, Dean J. Naisbitt

P164 Vancomycin-specific T cell responses and teicoplanin cross-reactivity

Wei Yann Haw, Marta E. Polak, Carolann Mcguire, Michael R. Ardern-Jones

Poster walk 19: BAT and biomarkers (P165–P173)

P165 A combination of early biomarkers useful for the prediction of severe ADRs

Yumi Aoyama, Tetsuo Shiohara

P166 Basophil activation test in the diagnostic approach of reactions during general anaesthesia

Ana Moreira, Susana Cadinha, Patrícia Barreira, Ana Castro Neves, Daniela Malheiro, Sara Correia, J. P. Moreira Da Silva

P167 IL-10 can be related to successful desensitization

Asli Gelincik, Semra Demir, Fatma Sen, Hamza Ugur Bozbey, Muge Olgac, Derya Unal, Raif Coskun, Bahauddin Colakoglu, Suna Buyuozturk, Esin Çatin-Aktas, Gunnur Deniz

P168 Immediate reactions to proton pump inhibitors: value of basophil activation test

Maria Salas, Jose Julio Laguna, Esther Barrionuevo, J. Dionicio, Tahia Fernandez, R. Gonzalez-Mendiola, I. Olazabal, Maria Dolores Ruiz, Miguel Blanca, Cristobalina Mayorga, Maria José Torres

P169 Improvement of the elevated tryptase criterion to discriminate IgE from non-IgE mediated allergic reactions

Gabriel Gastaminza, Alberto Lafuente, Carmen D’Amelio, Amalia Bernad, Olga Vega, Roselle Catherine Madamba, M. Jose Goikoetxea, Marta Ferrer, Jorge Núñez

P170 Low expression of Tim-3 could serve as a biomarker for control and diagnose maculopapular exanthema induced by drugs

Tahia Diana Fernández, Inmaculada Doña, Francisca Palomares, Rubén Fernández, Maria Salas, Esther Barrionuevo, Maria Isabel Sanchez, Miguel Blanca, Maria José Torres, Cristobalina Mayorga

P171 Role of basophil activation test using two different activation markers for the diagnosis of allergy to fluoroquinolones

Esther Barrionuevo, Tahía Fernandez, Arturo Ruiz, Adriana Ariza, Maria Salas, Inmaculada Doña, Ana Molina, Miguel Blanca, Maria Jose Torres, Cristobalina Mayorga

P172 The importance of basophil activation test in anaphylaxis due to celecoxib

Amalia Bernad Alonso, Carmen D’Amelio Garófalo, Olga Vega Matute, Marta Ferrer Puga, María José Goikoetxea Lapresa, Roselle Catherine Yu Madamba, Gabriel Gastaminza Lasarte

P173 The role of basophil activation test in the diagnosis of immediate type drug hypersensitivity to betalactam antibiotics

Antonia Thinnes, Hans F. Merk, Jens Malte Baron, Martin Leverkus, Galina Balakirski

Poster walk 20: TCR recognition, cellular (P174–P183)

P174 Characterisation of the effect of co-inhibitory signalling on the activation of drug-derived antigen-specific T-cells

Andrew Gibson, Monday Ogese, Lee Faulkner, B. Kevin Park, Dean J. Naisbitt

P175 Characterization of drug hapten-specific T cell responses in piperacillin hypersensitive patients

Zaid Al-Attar, Fiazia Yaseen, Xiaoli Meng, Rozalind Jenkins, Paul Whitaker, Daniel Peckham, Lee Faulkner, John Farrel, Kevin Park, Dean Naisbitt

P176 Characterization of the response of T-cells to telaprevir and its metabolite in normal volunteers

Zaid Al-Attar, Khetam Alhilali, Yanni Xue, John Farrell, Lee Faulkner, Kevin Park, Dean Naisbitt

P177 Characterization of the T cell receptor signatures of drug-responsive T cells

Patricia Illing, Nicole Mifsud, Heidi Fettke, Jeffrey Lai, Rebecca Ho, Patrick Kwan, Anthony Purcell

P178 Defining the signals between hepatocytes and immune cells in idiosyncratic drug-induced liver injury (DILI)

Monday O. Ogese, Lee Faulkner, B. Kevin Park, Catherine Betts, Dean J. Naisbitt

P179 Development of novel chemicals that do not bind to HLA-B*57:01 or activate CD8 + T-cells through modification of the 6-amino cyclopropyl group of abacavir

Paul Thomson, John Farrell, Mohammad Alhaidari, Neill Berry, Paul M. O’Neill, B. Kevin Park, Dean J. Naisbitt

P180 Generation and characterization of dapsone- and nitroso-dapsone-specific T-cell clones using lymphocytes from healthy volunteers

Abdulaziz Alzahrani, Monday O. Ogese, John Farrell, Lee Faulkner, Andrew Gibson, Arun Tailor, B. Kevin Park, Dean J. Naisbitt

P181 Identification of benzylpenicillin-hapten peptides responsible for naïve T-cell activation and immunization of allergic patients to penicillin

Marie Eliane Azoury, Lucia Fili, Rami Bechara, Noémie Scornet, Cathy Nhim, Richard Weaver, Nancy Claude, Delphine Joseph, Bernard Maillere, Paola Parronchi, Marc Pallardy

P182 Massive expansion of clonotypic and polycytotoxic CD8+ T cells in toxic epidermal necrolysis

Axel Patrice Villani, Aurore Rozières, Benoît Bensaïd, Mathilde Tardieu, Floriane Albert, Virginie Mutez, Tugba Baysal, Marc Pallardy, Janet Maryanski, Jean-François Nicolas, Osami Kanagawa, Marc Vocanson

P183 Pharmaco-immunological synapse of HLA-drug-TCR in SCAR

Shuen-Iu Hung

Poster walk 21: new in vitro methods, haptens, etc. (P184–P194)

P184 Amoxicillin-clavulanate forms distinct multiple haptenic structures on human serum albumin in patients

Xiaoli Meng, Arun Tailor, Caroline J. Harrison, Rosalind E. Jenkins, Paul Whitaker, Neil S. French, Dean J. Naisbitt, B. Kevin Park

P185 Dendrimeric antigens for studying the influence of penicillin determinants orientation on IgE recognition

Maria Isabel Montañez, Cristobalina Mayorga, Francisco Najera, Adriana Ariza, Tahia D. Fernandez, Maria Salas, Angela Martin-Serrano, Miguel Blanca, Ezequiel Perez-Inestrosa, Maria Jose Torres

P186 Dendrimeric antigens on solid supports: designed materials for IgE quantification

Yolanda Vida, Maria Isabel Montañez, Noemi Molina, Daniel Collado, Francisco Najera, Adriana Ariza, Maria Jose Torres, Cristobalina Mayorga, Ezequiel Perez-Inestrosa

P187 Development of a screening assay for drug hypersensitivity using naïve T cells from donors with seven different HLA class I risk alleles

Lee Faulkner, Sally Wood, Ana Alfirevic, Munir Pirmohamed, Dean J. Naisbitt, B. Kevin Park

P188 Different patterns of recognition of structures derived from amoxicillin by IgE antibodies from patients with immediate hypersensitivity reactions to betalactams

Adriana Ariza, Cristobalina Mayorga, María Isabel Montañez, María Salas, Inmaculada Doña, Ángela Martín-Serrano, Ezequiel Pérez-Inestrosa, Dolores Pérez-Sala, Miguel Blanca, Antonio E. Guzmán, María José Torres

P189 High-resolution typing of HLA polymorphism and T-cell receptor repertoire for severe adverse drug reactions based on the cost-effective next-generation sequencing approaches

Tai-Ming Ko, Yuan-Tsong Chen, Jer-Yuarn Wu

P190 Identification and fate of intracellular proteins haptenated by amoxicillin

Francisco J. Sánchez-Gómez, Juan M. González-Morena, Yolanda Vida, Ezequiel Pérez-Inestrosa, Miguel Blanca, María J. Torres, Dolores Pérez-Sala

P191 In vitro detection of terbinafine protein adducts

Arun Tailor, Toru Usui, Yanni Xue, Xiaoli Meng, Dean J. Naisbitt, B. Kevin Park

P192 MicroRNAs dysregulation in PBMCs from drug hypersensitivity patients during drug challenge in vitro

Alejandra Monroy Arreola, Jesus Agustin Badillo Corona, Silvia Mendez Flores, Judith Dominguez Cherit, Dean J. Naisbitt, Noe Valentin Duran Figueroa, Jose Luis Castrejon Flores

P193 NSAIDs-exacerbated cutaneous disease: high throughput gene expression profiling

José Antonio Cornejo-García, James Perkins, Natalia Blanca-López, Diana Pérez-Alzate, Raquel Jurado-Escobar, Inmaculada Doña, Gador Bogas, María J. Torres, Gabriela Canto, Miguel Blanca

P194 Utility of skin tests in non-immediate reactions to amoxicillin

Luis Mario Tubella Marti, Fernando Pineda De La Losa, Francisca Arribas Poves, Jaime Tubella Lopez, Teodora Lopez Santiago

## Poster Walk 11: Miscellaneous drug hypersensitivity 2 (P92–P94, P96–P101)

### P92 16 years of experience with proton pump inhibitors (PPIs)

#### Javier Dionicio Elera, Cosmin Boteanu, Maria Aranzazu Jimenez Blanco, Rosario Gonzalez-Mendiola, Irene Carrasco García, Antonio Alvarez, Jose Julio Laguna Martinez

##### Hospital de la Cruz Roja Madrid, MADRID, Spain

**Correspondence:** Javier Dionicio Elera

*Clinical and Translational Allergy* 2016, **6(Suppl 3)**:P92

**Background:** Proton Pump Inhibitors (PPIs) are commonly used for the treatment of acid-related disorders. They are generally well tolerated, with a low incidence of adverse effects (1 %). Omeprazole is the most common PPIs used and its intake has tripled from 2003 to 2012 in Spain. Although hypersensitivity reactions to PPIs are rare, they are being more reported in recent years maybe due to the growing use of these drugs.

The aim of this study was to review our experience with the use of PPIs in the last 16 years.

**Materials and methods:** We undertook a retrospective analysis of 10,858 patients who attended to the Drug Allergy Unit of Hospital de la Cruz Roja-Madrid, from 1999 to 2014.

We divided our patients in two groups according to the increase of intake of PPIs. Group 1 from 1999 to 2008 and Group 2 from 2009 to 2014.

We consider positive cases those who had Positive skin Prick test or positive drug provocation test or positive basophil activation test or suggestive history of PPIs allergy. We excluded patients who quit or refused the study.

**Results:** A total of 253 patients (2.3 %) were attended in the Allergy Unit with suspected history of PPIs allergy in the last 16 years. From those, 16 (6.3 %) were positive cases.

In group 1, we included 5563 patients, from those, 91 were suspected cases, and 4 (4.4 %) of them were positive.

In group 2, we included 5295 patients, from those 162 were suspected cases, and 12 (7.4 %) of them were positive.

**Conclusions:** In our experience, we found an increase in the number of reactions due to PPIs in the last years. Our results are according to the literature: Hypersensitivity reactions to PPIs are rare.

### P93 Allergy evaluation of quinolone induced adverse reactions

#### Jaume Martí Garrido, Carla Torán Barona, Carolina Perales Chorda, Ramón López Salgueiro, Miguel Díaz Palacios, Dolores Hernández Fernández De Rojas

##### IIS La Fe, Valencia, Spain

**Correspondence:** Jaume Martí Garrido

*Clinical and Translational Allergy* 2016, **6(Suppl 3)**:P93

**Background:** The increasing use of quinolone antibiotics is associated with an increase in hypersensitivity reactions. The objective of this study is to analyse the cases of suspected allergy to quinolones referred to an allergy department in a tertiary level centre during 2015.

**Materials and methods:** We performed a descriptive, retrospective study. Cases were identified from the medical record administrator programme by searching the terms “quinolone, norfloxacin, moxifloxacin, levofloxacin or ciprofloxacin”. We collected demographic and medical data, information of the adverse events, results of the allergy evaluation and the recommendations for the future use of quinolones.

**Results:** We analysed 60 cases (26 male/34 female) with an average age of 60.5 years (6–88). The most frequent clinical manifestations were cutaneous (70 %), 11 % digestive, 7 % respiratory, 7 % neurological, 4 % cardiovascular and 1 % renal. The most common symptom were pruritus (25 %) and erythema (24 %). The reaction was immediate (within minutes) in 37 % and delayed in 45 %. The type of reaction was not described in 18 % of cases. The involved quinolones were ciprofloxacin (40 %), levofloxacin (37 %), norfloxacin (8 %) and moxifloxacin (8 %). The most frequent route of administration was oral (66 %), followed by intravenous (30 %), topical (2 %) and otic (2 %). In two cases quinolones were directly banned after the adverse event due to the patient clinical condition or it was not considered necessary due to the lack of evidence on the reliability of the tests. Skin tests were performed in 40 % of cases, with positive results for ciprofloxacin 78 %, moxifloxacin 77 %, levofloxacin 57 % and none for norfloxacin. In a case of a generalized delayed reaction, patch tests were negative. In one case a fixed drug erythema due to norfloxacin was diagnosed without further check. In 10 cases of severe immediate reactions, BAT was performed 17 times with 4 positive results for ciprofloxacin and 3 for levofloxacin. In these cases quinolones were banned. All patients tolerated alternative quinolones to the one eliciting the adverse event, even when skin tests were positive.

**Conclusions:** Ciprofloxacin and levofloxacin were the most frequently involved quinolones. Skin symptoms were the most common clinical manifestations. Allergy evaluation included skin tests, BAT and controlled exposure in order to identify the responsible quinolones and search for safe alternatives. The sensitivity and specificity of these tests are still undetermined.

**Keywords:** Quinolone; Allergy

### P94 Bupropion-induced acute urticaria and angioedema, a case report

#### Emre Ali Acar^1^, Ayse Aktas^2^, Aylin Türel Ermertcan^3^, Peyker Temiz^4^

##### ^1^Celal Bayar University, Faculty of Medicine, Department of Internal Medicine, Manisa, Turkey; ^2^Celal Bayar University, Faculty of Medicine, Department of Allergy and Immunology, Manisa, Turkey; ^3^Celal Bayar University, Faculty of Medicine, Department of Dermatology, Manisa, Turkey; ^4^Celal Bayar University, Faculty of Medicine, Department of Pathology, Manisa, Turkey

**Correspondence:** Emre Ali Acar

*Clinical and Translational Allergy* 2016, **6(Suppl 3)**:P94

**Background:** Bupropion is a new-generation antidepressant, while its sustained release (SR) formulation is used in smoking cessation for a long time successfully. Urticaria, pruritus and rashes can be seen in 1–4 % of the patients and anaphylactoid reactions like angioedema and dyspnea can be seen in 0.1–0.3 % of the patients using bupropion. We wanted to report a case with urticaria and angioedema due to SR bupropion treatment.

**Fig. 1:**
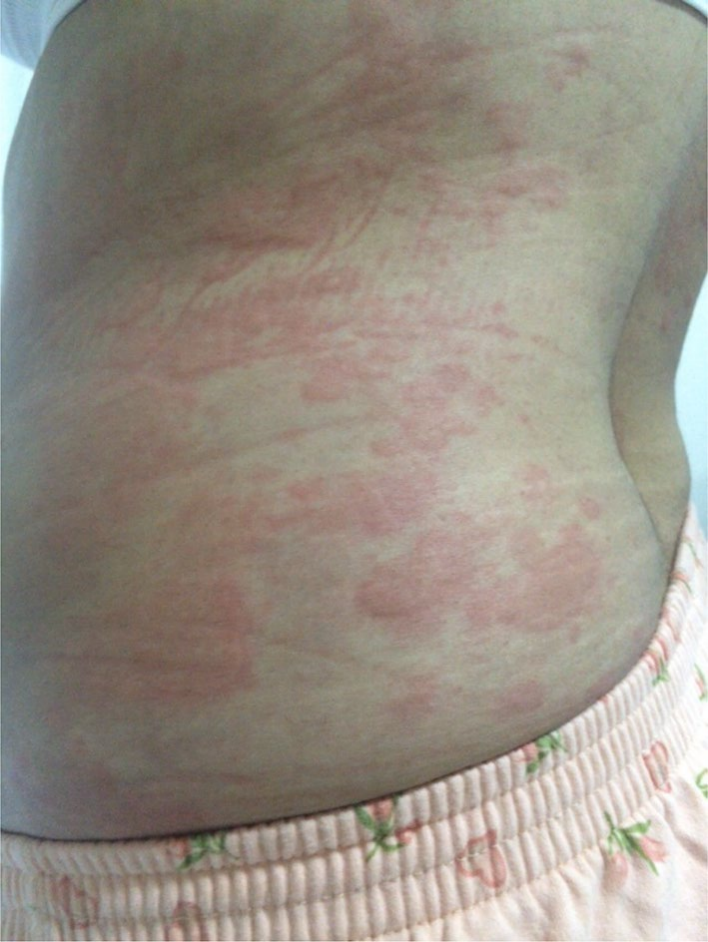
Urticarial lesions on trunk

**Report:** A-27-year-old woman without any disease history admitted to a hospital for smoking cessation. She had 15-pack-year of smoking history and she had started using 150 mg of SR bupropion. In first 3 days, she had used it once a day, then she started using twice a day. On tenth day of treatment, generalized rash with pruritus had been developed. In physical examination, she had generalized plaques and papules with edema and erythema on his neck, trunk, upper and lower extremites (Fig. 1). She had swelling on her lips as well. With suspicion of adverse drug reaction, skin biopsy was made. Other physical examination, blood and urinary laboratory findings were normal. With these clinical and histopathological findings, urticaria and angioedema diagnosis were made. She was hospitalized and bupropion treatment was stopped. Systemic corticosteroid and antihistaminic treatments were started. On third day of treatment, her skin lesions were regressed.

**How this report contributes to current knowledge:** Buproprion is a commonly used drug in smoking cessation clinics. Although it is rare, physicians should be cautious about urticaria, angioedema and anaphylaxis as adverse drug reactions of bupropion treatment.

**Consent:** Written informed consent was obtained from the patient for publication of this abstract and any accompanying images.

### P96 Delayed type hypersensitivity and study of cross-reactivity between proton-pump inhibitors

#### Chien-Yio Lin^1^, Chung-Yee Rosaline Hui^2^, Ya-Ching Chang^2^, Chih-Hsun Yang^2^, Wen-Hung Chung^2^

##### ^1^Department of Dermatology, Chang Gung Memorial Hospital, Linkou, Taiwan; ^2^Department of Dermatology, Drug Hypersensitivity Clinical and Research Center, Chang Gung Memorial Hospital, Linkou, Taiwan

**Correspondence:** Chien-Yio Lin

*Clinical and Translational Allergy* 2016, **6(Suppl 3)**:P96

**Background:** There are six PPIs available for clinical use, which could be set into two groups, omeprazole/esomeprazole/pantoprazole versus lansoprazole/dexlansoprazole/rabeprazole, based on the same basic skeleton, but differed by virtue of substitutions on both rings. The former three have changes in their benzimidazole ring, whereas the latter three have changes in their pyridine ring. Although proton-pump inhibitors (PPI) are relatively well tolerated, some patients may develop hypersensitivity reactions, which vary from mild symptoms to life-threatening severe cutaneous adverse reactions (SCARs), such as Stevens–Johnson syndrome (SJS), toxic epidermal necrolysis (TEN), acute generalized exanthematous pustulosis (AGEP), and drug rash with eosinophilia and systemic symptoms (DRESS). Clinicians often overlook the potential of PPI-related SCARs.

**Materials and methods:** We retrospectively analyzed patients of PPI-related cutaneous adverse reactions from Chang Gung Memorial Hospital health system during January 2003 to December 2015. We analyzed the causative PPIs, cutaneous manifestation, organ involvement, treatment, and complications. We also assessed the cross-hypersensitivity to other PPI after the hypersensitivity episode. The causality of the culprit drugs was further confirmed by in vitro lymphocyte activation test (LAT) and patch test in some cases.

**Results:** There were 48 cases of PPI-related cutaneous adverse reactions, with maculopapular eruption (MPE, n = 20), DRESS (n = 10), and SJS/TEN (n = 9), contributing the most. Esomeprazole was the most common causative PPI (n = 23, 47.9 %), and the liver was the most frequently involved internal organ (26.7 %). One patient (2.1 %) died of esomeprazole-induced TEN. Seven patients allergic to one group of PPI would able to tolerate the other different structured PPI group, whereas two patients showed cross-hypersensitivity when shifting to alternative PPI of the same group with similar structure. The drug causality and cross-reactivity were confirmed by lymphocyte activation test in 53.8 % patient of whom tested; besides, 2 of 5 patient with patch test performed showed corresponding result to the LAT.

**Fig. 2:**
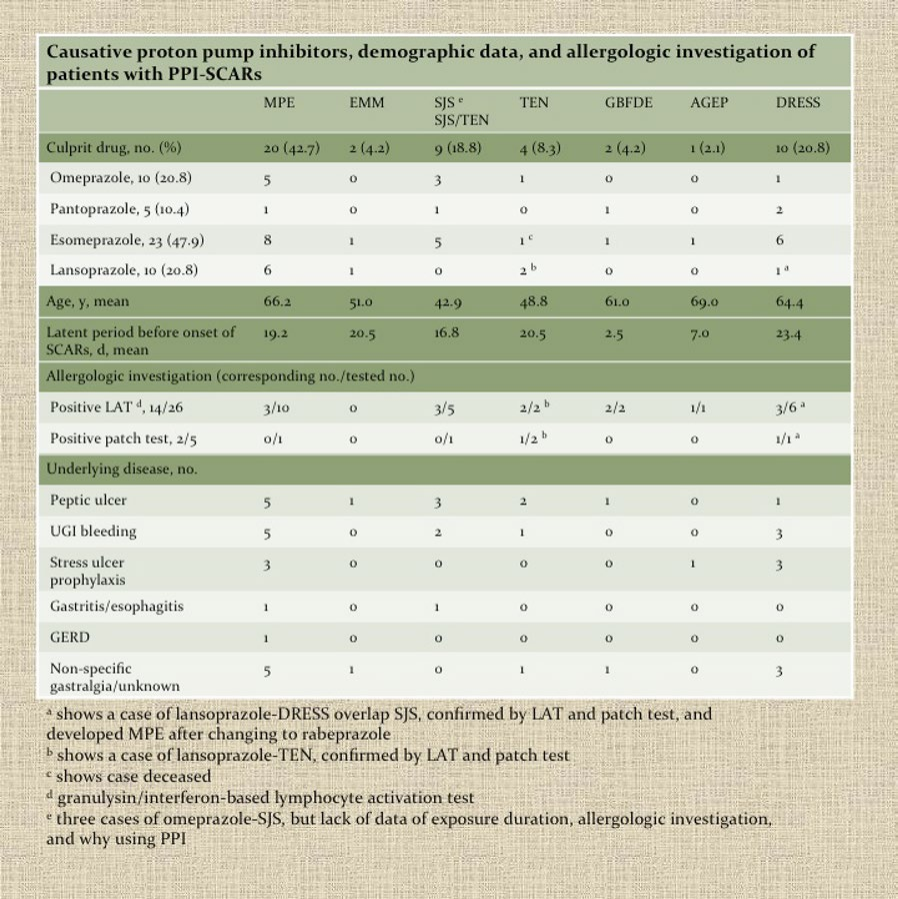
Causative proton pump inhibitors, demographic data, and allergologic investigation of patients with PPI-SCARs

**Conclusions:** The clinicians should be cautious of PPI-related SCARs. The potential of cross-hypersensitivity to similar structured PPI should be aware of, and shifting to different structured PPI could be considered when PPI is the treatment of choice (Fig. 2).

### P97 Diagnostic work-up in suspected hypersensitivity to proton-pump inhibitors: looking at cross-reactivity

#### Fabrícia Carolino^1^, Diana Silva^2^, Eunice Dias De Castro^1^, Josefina R. Cernadas^1^

##### ^1^Serviço de Imunoalergologia, Centro Hospitalar São João E.P.E., Porto, Portugal; ^2^Serviço de Imunoalergologia, Centro Hospitalar São João E.P.E.; Laboratório de Imunologia, Faculdade de Medicina, Universidade do Porto, Porto, Portugal

**Correspondence:** Fabrícia Carolino

*Clinical and Translational Allergy* 2016, **6(Suppl 3)**:P97

**Background:** Proton-pump inhibitors (PPI) are widely used in clinical practice and there are multiple reports of hypersensitivity reactions (HSR), most of them immediate, and cross-reactivity (CR) between PPI. Specificity of skin tests has to be determined and there are currently no specific recommendations from the ENDA group. **Aim:** We present a consecutive case series of patients with suspected PPI HSR.

**Materials and methods:** The present study is a case series of patients evaluated in our Drug Allergy Unit for suspected PPI HSR, in an 8-year period. CR in skin prick tests (SPT) and intradermal tests (IDT) was assessed using the commercially available parenteral PPI formulations (esomeprazole 20 mg/ml, omeprazole 4 mg/ml, pantoprazole 4 mg/ml) and crushed tablets in saline (lansoprazole 15 mg/ml, rabeprazole 10 mg/ml) for SPT. IDT were only performed with parenteral formulations (1/1000–1/1 dilutions), due to safety and accuracy reasons.

**Results:** In the studied period, 19 patients (11 female; mean ± SD age 50.7 ± 14.2 years) were assessed for the suspected HSR. PPI were mainly prescribed for dyspepsia/stomach ache (n = 11) or *Helicobacter pylori* eradication (n = 4). Implicated PPI were omeprazole (n = 13), esomeprazole (n = 4), pantoprazole (n = 2), lansoprazole (n = 2), with 2 patients reacting to different PPI (omeprazole/lansoprazole and omeprazole/pantoprazole). Eight patients had immediate reactions (6 anaphylaxis and 2 urticaria) and 3 of them had positive SPT (one had positive SPT to both omeprazole and pantoprazole). Seven patients (5 urticaria, 1 anaphylaxis and 1 MPE) had positive IDT to omeprazole only (undiluted) and 1 patient (anaphylaxis) had a positive IDT to pantoprazole alone (1/100). Cutaneous CR (this is, positive IDT for >1 PPI) was present in 3 patients, as described below:

- one patient with anaphylaxis to omeprazole and positive IDT to omeprazole (1/1000) plus esomeprazole (1/10);
- another patient with anaphylaxis to omeprazole and positive IDT to omeprazole (1/10) plus pantoprazole (1/1);
- and a patient with urticaria to esomeprazole and positive IDT to esomeprazole (1/10) plus pantoprazole (1/10).

Non-atopic controls were also tested for PPI.

**Conclusions:** Cross-reactivity in 4 patients can be explained by the similar chemical structure of PPI. Additional multicentre studies are needed to standardize procedures for skin tests with PPI. Oral challenges are also important to increase the diagnostic accuracy to this pharmacological group.

**Keywords:** Drug hypersensitivity; Proton-pump inhibitors; Skin tests; Cross-reactivity

### P98 Management of infusion-related hypersensitivity reactions to enzyme replacement therapy for lysosomal diseases

#### Luis Felipe Ensina^1^, Carolina Aranda^1^, Ines Camelo Nunes^1^, Alex Lacerda^1^, Ana Maria Martins^1^, Ekaterini Goudouris^2^, Marcia Ribeiro^2^, José Francisco Da Silva Franco^3^, Leandra Queiroz^4^, Dirceu Solé^1^

##### ^1^Federal University of São Paulo, São Paulo, Brazil; ^2^Federal University of Rio de Janeiro, Rio De Janeiro, Brazil; ^3^Pontificia Universidade Católica de Campinas, Campinas, Brazil; ^4^Private Practice, Goiania, Brazil

**Correspondence:** Luis Felipe Ensina

*Clinical and Translational Allergy* 2016, **6(Suppl 3)**:P98

**Background:** Enzyme replacement therapy (ERT) has been used in the treatment of lysosomal diseases (LD). ERT with human recombinant enzymes has shown to slow disease progression and improve the quality of life. Infusion-related reactions (IRR) to ERT can occur and be severe, including hypersensitivity reactions (HSRs) such as anaphylaxis. A diagnostic and treatment protocol was proposed to manage those reactions.

**Materials and methods:** Patients under ERT for Mucopolysaccharidosis (I, II and VI), Gaucher, Fabry and Pompe diseases under treatment in 7 centers from Brazil were assessed from January 2011 through December 2015. In the presence of suggestive signs or symptoms of an adverse reaction, ERT was stopped and skin tests for specific IgE assay were performed. In patients with symptoms of acute infection after infusion and with negative skin tests, ERT was maintained at the same infusion rate. In patients without a history of infection and with negative tests, ERT was maintained with an increased infusion rate. For those patients with positive ST that continued reacting after adjustments in the IR, a 3 bags 12-steps desensitization protocol was generated.

**Results:** Thirteen patients presented a suggestive HSR with positive skin tests, during treatment with laronidase (n = 3), galsulfase (n = 2), betagalsidase (n = 7), imiglucerase (n = 1). Urticaria was the most commom symptom observed (50 %), followed by fever (20 %), chills (10 %), cough (10 %) and anaphylaxis (10 %). Of the 911 desensitizations performed, 20 % induced mild reactions, but all patients received their full target dose. No severe, life-threatening HSRs or deaths occurred during the procedure.

**Conclusions:** Considering the importance of ERT in the treatment of LD, a standardized diagnostic protocol for IRR allows us to establish a correct diagnosis and propose an efficient treatment algorithm, which includes infusion rate modification and desensitization.

**Keywords:** Desensitization; Lysosomal diseases

### P99 Management of insulin allergy with continuous subcutaneous insulin infusion

#### Ceyda Tunakan Dalgiç, Aytül Zerrin Sin, Fatma Düsünür Günsen, Gökten Bulut, Fatma Ömür Ardeniz, Okan Gülbahar, Emine Nihal Mete Gökmen, Ali Kokuludag

##### Ege University Medical Faculty, Department of Allergy and Clinical Immunology, Izmir, Turkey

**Correspondence:** Ceyda Tunakan Dalgiç

*Clinical and Translational Allergy* 2016, **6(Suppl 3)**:P99

**Background:** Insulin allergy is uncommon, particularly in patients with Type 2 diabetes mellitus (DM). Management of the condition can be difficult. We report a patient with Type 2 DM and insulin allergy successfully managed with continuous subcutaneous insulin infusion (CSII).

**Report:** 35 years old woman diagnosed as type 2 diabetes mellitus used detemir insulin at first. On the third day of detemir, 12 h after the dose, a pruriginous papule occured at the site of injection area. Local reaction was continued. On the seventh day, detemir was changed with NPH. Local reaction occured at first dose of NPH. All of the local reactions were late onset. Therapy was changed to glargine. One hour after the first dose of glargine, local reaction and generalize pruritus were occurred. All insulin types were stopped and 4 weeks later, skin tests were performed. Prick tests were negative. Intradermal tests were positive with the dilutions of 1/100 detemir, 1/100 glargine, 1/1000 NPH, 1/1000 regular insulin. Glulisin, aspart and lispro were negative. Insulin specific IgE [194 Ku/l (0–87)] and anti-insulin antibody 47.8 % (reference <8.2) were high and specific Ig G4 was normal [35 mg/dl (0–125)]. The therapy started with glulisin and there wasn’t any reaction at first. Therefore, since blood glucose and serum glycated hemoglobin A1c levels (10 %) were still higher, as a long acting insulin, NPH had to be added together with antihistaminic. No reaction was observed with NPH. At the 20th day of glulisin, late onset local reactions were seen again. Glulisin was changed to aspart. Similar reactions were seen with those insulins. Finally, the only insulin which has never been used before was lispro. We suggested CSII pump with lispro. After this method, insulin hypersensitivity was successfully treated and glycemic control was achieved.

**How this report contributes to current knowledge:** Allergy to insulin analogues is rare and requires early diagnosis, leading to a major therapeutic challenge. In this case, local reactions continued although types of insulins and application areas were changed. Interestingly, blood glucose was increasing to uncontrolled levels always following by the cutaneous reactions, also. As we consider anti-insulin antibodies and late onset local reactions, insulin allergy is thought to be mediated by type 1 and type 2 hypersensitivity reactions in this case.

**Consent:** Written informed consent was obtained from the patient.

### P100 Off-label use of icatibant for management of serious angioedema associated with angiotensin inhibitors

#### Ana M. Montoro De Francisco, Talía Mª De Vicente Jiménez, Adriana M. Mendoza Parra, Angella M. Burgos Pimentel, Amelia García Luque

##### Hospital Central de la Defensa, IMIDEF, Madrid, Spain

**Correspondence:** Ana M. Montoro De Francisco

*Clinical and Translational Allergy* 2016, **6(Suppl 3)**:P100

**Background:** Angioedema is a serious, infrequent and well-known Adverse Drug Reaction (ADR) to antihypertensive drugs: Inhibitors of Angiotensin Converting Enzyme (ACEI), Angiotensin II Receptor Blocker (ARB) and Direct Inhibitor of Renin (DIR). ACEI/ARB/DIR are largely used worldwide, therefore, the morbidity and mortality from ADR could be considerable. No medications are currently approved for management of this ADR.

Icatibant, a bradykinin receptor type 2 antagonist, is a potential treatment for a serious bradykinin-mediated angioedema associated with angiotensin inhibitors.

**Materials and methods:** Design: A case series of 100 patients with angioedema associated with ACEI/ARB/IDR.

Scope: Allergy service, Hospital Central de la Defensa, Madrid.

Period: March 2009 to December 2015.

Main Variables assessed: demographic and clinical variables, treatment, and evolution.

The patients have given written informed consent for the publication research.

**Results:** One hundred patients with episodes of angioedema all of them associated with ACEI/ARB/DIR. In addition to angioedema, patients showed cough, conjunctivitis, pruritus and rhinitis.

Twenty had severe angioedema with airway compromise, speech impairment and hoarseness and poor response to antihistamines and corticosteroids which required hospitalization. They were treated with icatibant 30 mg subcutaneous injection improving in their symptoms after 20 min–3.3 h. All patients experienced complete resolution of angioedema and avoidance of intubation and tracheotomy.

Age 66.3 years (37–90), 12 females and 8 males. Ten patients had previously experienced serious angioedema attacks.

Eight drugs were involved: ACEI 15 (enalapril, captopril and lisinopril), ARBII 4 (valsartan, losartan, olemesartan and ibersartan) and DIR 1 (aliskiren). More frequently involved drug was enalapril (9 cases).

**Conclusions:** Angioedema associated to angiotensin inhibitors is a serious ADR, it is rare but not unusual due to the widespread use of this class of drugs. Icatibant used off-label helped to improve the acute angioedema attacks and avoidance of intubation and tracheotomy.

**Keywords:** Angioedema; Angiotensin inhibitors; Icatibant

### P101 Thiocolchicoside anaphylaxis: an unusual suspect?

#### Luis Amaral, Fabricia Carolino, Leonor Carneiro Leão, Eunice Castro, Josefina Cernadas

##### Serviço de Imunoalergologia, Centro Hospitalar de São João E.P.E., Porto, Portugal

**Correspondence:** Luis Amaral

*Clinical and Translational Allergy* 2016, **6(Suppl 3)**:P101

**Background:** Thiocolchicoside is a sulfureted semi-synthetic molecule derived from colchicoside and is used frequently as adjuvant treatment for acute muscle contractures in spinal pathology. Immediate hypersensitivity reactions are rarely reported and only two documented cases of immediate anaphylaxis are described in the literature. Moreover, the allergy diagnosis with validated skin tests is lacking.

**Materials and methods:** We describe a first case of a 56-year-old woman that reported generalized pruritus, a rash involving palms and soles, dyspnea and colicky abdominal pain, 5 min after intramuscular (IM) administration of thiocolchicoside. These symptoms quickly reversed with IM adrenaline and intravenous (IV) hydrocortisone. A few months later we received a second case of a 52-year-old man who experienced dizziness, malaise and hypotension, 40 min after IM administration of thiocolchicoside and diclofenac. This clinical scenario was inverted with volume reposition with saline, IV hydrocortisone and adrenaline wasn’t administered.

**Results:** In the first case the emergency lab results showed serum tryptase 47 μg/l. Skin Prick test (SPT) (2 mg/ml) was negative and 5 min after performing intradermal test (IDT) (2 mg/ml) the patient experienced throat tightness that was reversed with ebastine 20 mg. In the second case the SPT (2 mg/ml) was negative and IDT (2 mg/ml) was positive with a 7 mm wheal diameter. The skin tests and oral provocation with diclofenac were negative.

SPT and IDT with the same concentration were also negative in 6 patients with suspected immediate reaction to thiocolchicoside, confirmed by negative drug provocation test.

**Conclusions:** Thiocolchicoside is often administered as an adjuvant therapy with non-steroidal anti-inflammatory drugs and is probably underestimated as a cause of immediate hypersensitivity reactions. Since the skin tests are not yet validated, many times it can be forgotten on the diagnostic workup. To our knowledge, this is the third study reporting documented anaphylaxis to thiocolchicoside and the first to support this diagnosis with intradermal tests.

**Consent:** Written informed consent was obtained from the patient for publication of this abstract and any accompanying images.

## Poster Walk 12: Betalactam hypersensitivity (P102–P111)

### P102 A curious delayed reading: a case report of a β-lactam allergy in a child

#### Nicole Pinto, Joana Belo, João Marques, Pedro Carreiro-Martins, Paula Leiria-Pinto

##### Hospital de Dona Estefânia, Centro Hospitalar de Lisboa Central, Lisbon, Portugal

**Correspondence:** Nicole Pinto

*Clinical and Translational Allergy* 2016, **6(Suppl 3)**:P102

**Background:** β-lactam antibiotics are commonly prescribed drugs worldwide and the most frequent cause of adverse drug reaction mediated by immunological mechanisms. Nonimmediate reactions, specially maculopapular and urticarial exanthems, are common. Skin tests are used to evaluate drug hypersensitivity and delayed reading might be useful in the diagnosis.

**Report:** A 2.5 year old girl, with no relevant past medical history, was referred to our outpatient clinic for suspected drug allergy to β-lactams. The patient had been admitted to the hospital for acute mastoiditis. Upon admission, amoxicillin-clavulanic acid (Ax/C), which she had been taking for 4 days, was discontinued due to vomiting and she was started on triple IV antibiotherapy with ceftriaxone, vancomycin and metronidazole. On the 15th day of treatment, physical examination revealed a diffuse pruriginous maculopapular rash and fever, without palpable adenopathies. C-reactive protein was increased, eosinophil count was within normal range values and serologies for EBV and CMV were negative. An allergic reaction to ceftriaxone was suspected and the drug was discontinued by the 23rd day of treatment. Due to persistence of symptoms, the remaining 2 antibiotics were also discontinued 3 days later. Skin biopsy was suggestive of erythema multiforme and a course of systemic corticotherapy was started with resolution of symptoms. Skin prick tests and intradermal tests (IDT) with Ax/C, penicillin, cefuroxime and ceftriaxone were performed, using the maximal non-irritant dose, all of which were negative on immediate reading. 48 h later, both the prick and IDT were positive for Ax/C as well as the IDT for ceftriaxone.

**How this report contributes to current knowledge:** Few nonimmediate reactions to cephalosporin are confirmed, due to the fact that most are caused by infections. Nonetheless, allergic reactions can occur even in young children, and in our case, the culprit agents were confirmed by a positive skin prick test on delayed reading. Skin prick tests and IDT with delayed readings seem to be useful in the evaluation of these reactions.

**Consent:** Written informed consent was obtained from the patient for publication of this abstract and any accompanying images.

### P103 Betalactam-induced hypersensitivity: a 10-years’ experience

#### Amel Chaabane, Haifa Ben Romdhane, Nadia Ben Fredj, Zohra Chadly, Naceur A. Boughattas, Karim Aouam

##### Faculty of Medicine/University hospital/University of Monastir, Monastir, Tunisia

**Correspondence:** Karim Aouam

*Clinical and Translational Allergy* 2016, **6(Suppl 3)**:P103

**Background:** Betalactams hypersensitivity remains overestimated because of the lack of an objective diagnosis tool leading to unjustified therapeutic alternatives.

This study has been performed in order to analyze the epidemiological, clinical and chronological features of betalactams hypersensitivity, to evaluate the skin tests value, and to establish a practical approach exploring such drug hypersensitivity.

**Materials and methods:** We included all adverse effects suspected to be induced by betalactams and notified to the pharmacovigilance unit of Monastir during 11 years. The drug imputability was established according to Begaud et al. method. Skin tests were performed as recommended by ENDA.

**Results:** Betalactms hypersensitivity was diagnosed in 168 patients. Almost all reactions were cutaneous mainly maculo-papular rashes. The severity was estimated at 11.3 %. The delayed reactions occurred in 60.7 % of cases. All reactions resolved after drug withdrawal. We identified 19 positive rechallenges. Skin tests were performed in 386 cases and were positive in 26.2 % of them. Penicillins were implicated in 62.5 % of cases. Almost all of immediate reactions were induced by penicillins. The hypersensitivity was selective to one betalactam in nearly half of cases. Cross-reactivity was objectified among penicillins in one-third of cases, between cefotaxime and cefazolin in one case, between penicillins and cephalosporins in 19 % of cases, involving piperacillin-tazobactam in two cases and imipenem in one case.

**Conclusions:** Through the current study, we have demonstrated that the betalactam hypersensitivity reactions are mostly delayed and non severe. The diagnosis was confirmed using skin tests which were useful not only in identifying the culprit drug but also in assessing cross reactivity. These findings have to be improved by the drug provocation test which is still not performed in our centre.

**Keywords:** Betalactam; Skin tests; Diagnosis; Cross reactivity

### P104 Cefazolin hypersensitivity: towards optimized diagnosis

#### Astrid P. Uyttebroek^1^, Chris H. Bridts^1^, Antonino Romano^2^, Didier G. Ebo^1^, Vito Sabato^1^

##### ^1^University of Antwerp, Faculty of Medicine and Health Sciences, Department of Immunology, Allergology, Rheumatology and Antwerp University Hospital, Immunology, Allergology, Rheumatology, Antwerp, Belgium; ^2^Allergy Unit, Complesso Integrato Columbus, Rome, Italy; IRCCS Oasi Maria S.S., Troina, Italy

**Correspondence:** Astrid P. Uyttebroek

*Clinical and Translational Allergy* 2016, **6(Suppl 3)**:P104

**Background:** Correct diagnosis of cefazolin hypersensitivity is not straightforward, mainly because of the absence of in vitro tests and uncertainties associated with the optimal skin test concentrations. Cross-reactivity patterns involving cefazolin suggest that cefazolin hypersensitivity is an isolated hypersensitivity.

**Objectives:** As a first objective of this study, we sought to confirm whether the application of a higher than 2 mg/ml test concentration could increase skin test sensitivity, and add to the diagnosis of cefazolin hypersensitivity. A second part of our study aimed at investigating the cross-reactivity between cefazolin and other β-lactam antibiotics.

**Materials and methods:** 66 patients referred to our outpatients’ clinic for diagnostic evaluation after experiencing perioperative anaphylaxis, and exposed to cefazolin, underwent skin testing with cefazolin up to 20 mg/ml. Patients exhibiting a positive skin test with cefazolin had a panel of skin tests with other β-lactams and, if indicated, graded drug challenges in order to study cross-reactivity.

**Results:** Increasing the skin test concentration from 2 to 20 mg/ml identified an additional 7/19 (27 %) patients, who would otherwise have displayed negative skin testing. The concentration was proven to be non-irritating in 30 cefazolin exposed control individuals in which an alternative culprit for peri-operative anaphylaxis was identified. Graded challenge testing, following negative skin testing, displayed that all the patients tolerated alternative β-lactam antibiotics. Of them, 11 individuals also tolerated an alternative cephalosporin, suggesting that cefazolin hypersensitivity (generally) is a selective allergy.

**Conclusions:** Increasing cefazolin skin test concentration up to 20 mg/ml benefits the sensitivity of diagnosis. Furthermore, our data further confirm that cefazolin hypersensitivity seems to be a selective allergy with good tolerance to other β-lactam antibiotics.

**Keywords:** Drug hypersensitivity; Cefazolin; Diagnosis; Skin testing

### P105 Clavulanic acid allergy: two cases report

#### Anabela Lopes^1^, Joana Cosme^1^, Rita Aguiar^1^, Tatiana Lourenço^1^, Maria-João Paes^1^, Amélia Spínola-Santos^1^, Manuel Pereira-Barbosa^2^

##### ^1^Immunoallergy Department - Hospital de Santa Maria, Centro Hospitalar Lisboa Norte, Lisbon, Portugal; ^2^Immunoallergy Department - Hospital de Santa Maria, Centro Hospitalar Lisboa Norte; Faculdade de Medicina de Lisboa, Lisbon, Portugal

**Correspondence:** Anabela Lopes

*Clinical and Translational Allergy* 2016, **6(Suppl 3)**:P105

**Background:** Beta-lactams (BL) are frequently prescribed antibiotics. Allergic reactions to BL can be immediate (IgE reactions) and non-immediate reactions (T cell reactions). There are two major types of BL allergic patients: cross-reactive patients that are allergic to common BL ring and selective patients, allergic to side chains. In the last years, several cases of clavulanic allergic patients were described. Frequently, the reactions to clavulanic acid (CL) are immediate and, until now, there are only reports of selective reactions to CL.

**Materials and methods:** The authors report two cases of unusual CL allergic patients. The patients were submitted to skin tests (ST) with penicilloylpolylysine (PPL), minor determinant mixture (MDM), amoxicillin (AX), CL (Diater laboratory), benzylpenicillin, ampicillin, cefuroxime, ceftriaxone, cefipime, determination of specific IgE to BL and oral provocation tests to alternative and culprit drugs.

**Results:** 1st case: male, 45 years old, presented to our allergy clinic with maculopapular rash, 7 days after beginning treatment with amoxicillin-clavulanic acid (AX/CL) and ibuprofen for pharygintis. He had previously received AX/CL with good tolerance. Drug provocation with ibuprofen was performed with negative results. Specific IgE to BL were negative. ST with PPL, MDM, AX, benzylpenicillin and AX/CL were negative and the patient was performed an oral AX/CL drug provocation. On the 5th day after drug provocation, he developed a generalized maculopapular rash. ST with CL was performed with a positive late reaction in the intradermal test (20 mg/ml) and a new drug provocation only with AX was negative then.

2nd case: female, 40 years old described 2 previous allergic reactions: generalized urticaria 10 min after the intake of one AX tablet at 36 years and an episode of anaphylaxis 5 min after one AX/CL tablet at 39 years. Specific IgE to BL were negative. ST with PPL, MDM, AX and CL and benzylpenicillin revealed positive results with a positive immediate prick test to AX and a intradermal test to CL (20 mg/ml). ST and drug provocation with cefuroxime and ceftriaxone were negative.

**Conclusions:** The first case concerns a rare delayed reaction to CL. To the best of our knowledge, the second case describes the first case of positivity to both AX and CL.

**Keywords:** Clavulanic acid; Delayed reactions; Selective reactions; β-Lactams

**Consent:** Written informed consent was obtained from the patient for publication of this abstract and any accompanying images.

### P106 Diagnosis of betalactam allergy in an allergy department

#### Cíntia Rito Cruz, Rute Pereira Dos Reis, Elza Tomaz, Ana Paula Pires, Filipe Inácio

##### Serviço de Imunoalergologia, Hospital de São Bernardo, Setúbal, Portugal

**Correspondence:** Cíntia Rito Cruz

*Clinical and Translational Allergy* 2016, **6(Suppl 3)**:P106

**Background:** Betalactams (BL) are still the most frequent drugs implicated in allergic reactions. All BL currently available may cause these reactions. Immediate hypersensitivity (IH) reactions usually appear within 1 h of drug intake and are mediated by specific IgE (sIgE) antibodies. They can be evaluated by different methods: clinical history, skin tests (ST), in vitro quantification of sIgE and drug provocation tests (DPT). The determination of sIgE has safety advantages over ST and DPT, but has low sensitivity. ST should be performed with PPL, MDM, penicillin G, amoxicillin and ampicillin. If these are all negative, a DPT should be executed to ultimately confirm or exclude allergy.

**Materials and methods:** Retrospective study that included patients who had undergone ST with PPL and MDM for investigation of BL allergy in 2014 and 2015. Data were then collected regarding the results of ST (PPL, MDM, penicillin G, amoxicillin, ampicillin and a cephalosporin), sIgE (penicillin G, penicillin V, ampicillin, amoxicillin and cefaclor) and DPT (to amoxicillin and/or a cephalosporin).

**Results:** One hundred and one patients (24 % male), with a mean age of 42 years old. The suspected BL was: penicillin G (40), amoxicillin (37), cephalosporins (6), ampicillin (2) and unknown (16). Eleven percent of the patients had positive ST to PPL or MDM, with only 1 having positive sIgE. Five of these patients underwent ST with an alternative cephalosporin (cefuroxime): 2 were positive and 3 were negative. Two of these underwent DPT, that were negative. Out of 90 patients with negative ST to PPL and MDM, 55 were submitted to ST with ampicillin and/or penicillin G and/or amoxicillin, being positive in 7 % of the patients; they all had negative sIgE. ST with amoxicillin didn’t add any extra information. Out of 84 patients with negative ST, 36 proceeded to DPT, which were positive in 6 % of the cases. Twelve patients underwent ST with a cephalosporin, being positive in 2 (when the suspected BL was also a cephalosporin). IH to BL was confirmed in 19 % of the cases.

**Conclusions:** Of the 19 cases of confirmed IH to BL, only 1 had positive sIgE, corroborating the extremely low sensitivity of the method. Only 19 % of the suspected cases of BL IH were confirmed, emphasizing the importance to refer patients to an Allergy Centre for investigation. Some patients never concluded the BL allergy study, and some are still undergoing. We highlight the need for new less expensive and time consuming tests, which would also reduce the number of dropouts.

**Keywords:** Betalactam allergy; Immediate hipersensitivity

### P107 Diagnostic work-up of 410 patients with suspicion of betalactam antibiotic hypersensitivity

#### Filipe Benito-Garcia, Inês Mota, Magna Correia, Ângela Gaspar, Marta Chambel, Susana Piedade, Mário Morais-Almeida

##### CUF Descobertas Hospital, Lisboa, Portugal

**Correspondence:** Filipe Benito-Garcia

*Clinical and Translational Allergy* 2016, **6(Suppl 3)**:P107

**Background:** The aim of this study was to characterize the activity developed at our drug allergy outpatients’ center with patients (pts) referred with suspicion of hypersensitivity (HS) to betalactam antibiotics (BL).

**Materials and methods:** Retrospective analysis of clinical files, in vivo/in vitro test results and drug provocation tests (DPT) from January 2011 to December 2015. All pts were studied according to standardized diagnostic procedures of ENDA/EAACI: serum specific IgE (sIgE) (ImmunoCAP^®^, ThermoFisher) to penicillin G/V, amoxicillin and ampicillin; skin prick and intradermal tests (IDT) to PPL/MD (DAP^®^, Diater), penicillin G, amoxicillin and cefuroxime with immediate and delayed reading. Other penicillin derivatives/cephalosporins were tested if they were the culprit drug. DPT with culprit drug was performed if previous investigation was negative. In confirmed cases an alternative BL was tested.

**Results:** 410 pts with suspicion of HS to BL were included: mean age was 34.6 (SD ±18) years, 21 % had <18 years and 68 % were female. Penicillins/derivatives were the main culprit drugs (314 pts), mostly amoxicillin (229 pts), 149 in association with clavulanic acid. Cephalosporins were mentioned by 42 pts, namely cefazolin (15 pts). Mucocutaneous symptoms were the most frequent manifestations (81 %); anaphylaxis occurred in 54 pts, 18 with loss of consciousness. HS to BL was confirmed in 88 pts (21.5 %), all with a DPT negative to an alternative BL. HS was excluded in 289 pts (70.5 %); 33 pts are under study. Confirmation of HS was made by: sIgE in 10 pts; skin testing in 65 pts and DPT in 13 pts (amoxicillin-11, clavulanic acid-2). Skin tests were positive to: amoxicillin-33, penicillin-16, PPL-15, MD-3, flucloxacilin-1 cefazolin-5, cefuroxime-1. Delayed reading of IDT was positive in 7 pts: penicillin-3, amoxicillin-4. A systemic reaction during skin testing occurred in 13 pts, in 3 with anaphylaxis: one child during IDT with amoxicillin (2.5 mg/ml) and 2 adults during IDT with amoxicillin (25 mg/ml).

**Conclusions:** To investigate the suspicion of HS to BL is of outmost importance because in most of these pts allergological workup is negative and HS to BL is excluded. Although the majority of confirmed cases are IgE-mediated, in about one-fourth a non-IgE mediated mechanism seems to be involved (positive DPT or positive delayed reading of IDT). Systemic reactions during IDT and DPT also reinforce the need of referral these pts to specialized centers.

**Keywords:** Adults; β-Lactams; Drug allergy; Children; Skin tests

### P108 Immediate selective hypersensitivity reactions to clavulanic acid

#### Alla Nakonechna^1^, Yurij Antipkin^2^, Tetiana Umanets^2^, Fernando Pineda^3^, Francisca Arribas^3^, Volodymyr Lapshyn^2^

##### ^1^Royal Liverpool and Broadgreen University Hospitals NHS Trust, Liverpool, United Kingdom; ^2^Institute of Pediatrics, Obstetrics and Gynaecology, Kiev, Ukraine; ^3^Diater Laboratorios, Madrid, Spain

**Correspondence:** Alla Nakonechna

*Clinical and Translational Allergy* 2016, **6(Suppl 3)**:P108

**Background:** Clavulanic acid (CLV) is b-lactamases inhibitor with weak antibacterial activity and low immunogenic capacity widely used in combination with beta-lactam antibiotics. Despite there is a number of reports of allergy to CVL in the combination with amoxicillin (AX-CVL), it is important to distinguish this kind of hypersensitivity as of the clinical implications of an amoxicillin allergy diagnosis.

**Materials and methods:** We present here 23 patients (14 women and 9 men, aged 23–62) with immediate hypersensitivity reactions to amoxicillin-clavulanic acid. Clinical symptoms occured within 30–60 min after AX-CLV intake and included urticaria/angioedema, bronchospasm and anaphylaxis.

Skin prick and intradermal tests with: major determinant benzylpenicilloyl poly-l-lysine (PPL-0.04 mg/ml), minor determinant mixture (MDM-0.5 mg/ml), CLV (20 mg/ml) and amoxicillin (20 mg/ml), all provided by Diater (Madrid, Spain), along with benzylpenicillin (10,000 IU/ml; Normon SA), ampicillin and cefuroxime (20 mg/ml; GlaxoSmithKline Beecham) were performed. In those cases with a positive skin test to CLV the AX-CLV (20/4 mg/ml) (GlaxoSmithKline Beecham) was also tested. Specific IgE antibodies against penicillin V, penicillin G, amoxicillin and ampicillin were measured by ELISA. Drug provocation test (DPT) with amoxicillin was performed in selective CLV hypersensitivity patients group.

**Results:** Specific IgE antibodies against penicillin V, penicillin G, amoxicillin and ampicillin were <0.35 UI/ml in all patients.

Among 23 patients with immediate hypersensitivity reactions to AX-CLV only 2 patients (8.7 %) had a positive skin test to benzylpenicillin determinants and 14 patients (60.8 %) to amoxicillin.

Among 7 patients (30.4 %) who had a positive intradermal test to selective CLV component and good tolerance to amoxicillin in DPT only 4 patients (17.4 %) were detected by AX-CLV.

**Conclusions:** Our investigation show: Immediate selective hypersensitivity reactions to CLV found in around 30 % of patients with immediate allergic reactions to AX-CLV combination.

Although allergy to clavulanic acid is infrequent, this molecule should be used in diagnostic assessment when evaluating an immediate reaction to AX-CLV and conventional skin testing with benzylpenicillin and amoxicillin determinants are negative.

Testing with selective CLV component is more sensitive and reliable than detection by AX-CLV for proving allergy to clavulanic acid.

### P109 Prevalence and incidence of penicillin hypersensitivity reactions in Colombia

#### Pablo Andrés Miranda, Bautista De La Cruz Hoyos

##### Universidad Nacional de Colombia, Cartagena, Colombia

**Correspondence:** Pablo Andrés Miranda

*Clinical and Translational Allergy* 2016, **6(Suppl 3)**:P109

**Background:** Penicillin remains one of the most widely used antibiotics in the world. Hypersensitivity reactions are more common adverse effects to this antibiotic.

**Materials and methods:** Records with allergy status of penicillin (ICD-10 code Z880) and adverse effects to penicillin (ICD-10 code Y400) of Information System of Social Protection (SISPRO) between 2010 and 2014 were included. To determine the prevalence and incidence of AHR, population estimates from the National Statistics Department of Colombia (DANE) were used.

**Results:** 781 cases with allergy status of penicillin and adverse effects to penicillin (PHR) between 2010 and 2015 were identified. 148 cases were confirmed news diagnoses in the same period. On average 156 cases of PHR per year were estimated. The estimated annual prevalence PHR were 3.3 cases per million (2010 = 4.3; 2011 = 3.3; 2012 = 3.8; 2013 = 2.7 and 2014 = 2.7). The estimated annual incidence PHR were 0.4 cases per million (2010 = 0.53; 2011 = 0.43; 2012 = 0.60; 2013 = 0.55 and 2014 = 0.25). Most cases it presenting in adult aged 60 years or younger, in children aged 5 years or younger and between 19 and 26 years age in 2010–2011; and in adult aged 60 years or younger and between 19 and 26 years age in 2012–2014.

**Conclusions:** Both under-diagnosis and over-diagnosis PHR are common in the world. Population studies with confirmatory tests PHR in Colombia are required (Table 1).

**Table 1:** Prevalence and incidence PHR Colombia 2010–2014

	2010 % (n)	2011 % (n)	2012 % (n)	2013 % (n)	2014 % (n)
Prevalence PHR Colombia Cases per million					
1–5 años	*20.4 (12)*	*13.9 (17)*	3.5 (26)	6.9 (33)	2.9 (32)
6–9 años	3.0 (35)	1.4 (24)	2.1 (6)	0.5 (12)	3.0 (5)
10–14 años	4.1 (13)	1.5 (6)	2.6 (9)	1.5 (2)	1.8 (13)
15–18 años	5.4 (14)	3.2 (5)	4.1 (9)	2.3 (5)	3.3 (6)
19–26 años	*12.7 (24)*	*10.2 (14)*	*12.5 (18)*	*7.7 (10)*	*7.7 (14)*
27–44 años	6.1 (45)	4.8 (36)	4.9 (44)	2.8 (27)	2.8 (27)
45–59 años	1.2 (39)	1.5 (31)	3.0 (32)	1.6 (19)	1.3 (19)
>60 años	*28.5 (13)*	*21.3 (17)*	*24.5 (34)*	*17.0 (19)*	*17.2 (15)*
Total	*4.3 (195)*	*3.3 (150)*	*3.8 (178)*	*2.7 (127)*	*2.7 (131)*
Incidence PHR Colombia Cases per million					
1–5 años	0.47 (2)	0.47 (2)	1.17 (5)	0.93 (4)	0.47 (2)
6–9 años	0.58 (2)	0.58 (2)	1.46 (5)	1.17 (4)	0.59 (2)
10–14 años	1.36 (6)	0.91 (4)	0.23 (1)	0.92 (4)	0.00 (0)
15–18 años	1.70 (6)	1.13 (4)	0.28 (1)	1.14 (4)	0.00 (0)
19–26 años	0.16 (1)	0.31 (2)	0.46 (3)	0.15 (1)	0.30 (2)
27–44 años	0.09 (1)	0.18 (2)	0.26 (3)	0.09 (1)	0.17 (2)
45–59 años	0.44 (3)	0.28(2)	0.69 (5)	0.54 (4)	0.26 (2)
>60 años	0.67 (3)	0.43 (2)	1.04 (5)	0.81 (4)	0.39 (2)
Total	*0.53 (24)*	*0.43 (20)*	*0.60 (28)*	*0.55 (26)*	*0.25 (12)*

**Keywords:** Penicillin; Allergy; Hipersensitivity

### P110 Selective sensitization to amoxicilin and clavulanic acid

#### Jose Julio Laguna Martinez, Aranzazu Jimenez Blanco, Javier Dionicio Elera, Cosmin Boteanu, Rosario Gonzalez-Mendiola, Marta Del Pozo

##### Allergy Unit. Hospital Central Cruz Roja, Madrid, Spain

**Correspondence:** Jose Julio Laguna Martinez

*Clinical and Translational Allergy* 2016, **6(Suppl 3)**:P110

**Background:** Beta-lactam (BL) antibiotics are the drugs most frequently involved in IgE-mediated allergic reactions.

Differences in the pattern of consumption are related with the specific BL involved in the allergic reactions and vary among countries and over the time. Since specific recognition of the amoxicillin (AX) side chain was described, a significant increase in the number of selective immediate reactions to AX and more recently selective reactions to Clavulanic acid (CLV) have been reported.

Patients with allergic reactions after AX-CLV administration can react to either AX or CLV. No evidence of cross reactivity between CLV and other BL has been reported.

**Report:** We present an atopic 44 year-old man referred to our allergy unit due in 2008 he took amoxicillin 500 mg/day as treatment for respiratory infection, 30 min after the first tablet he developed pharyngeal pruritus, cutaneous erythema and flushing following generalised itching and erythema.

**Method:** Skin prick test (SPT) and intradermal test (IDT) were performed using classic penicillin determinants, amoxicillin, ampicillin and clavulanic acid determinants according ENDA protocol.

Total IgE and specific IgE (Thermo Fisher Scientific) with penicillin determinants (penicilloyl G, amoxicilloy, penicilloyl V and ampicilloyl) were performed.

Drug provocation Test (DPT) if skin and in vitro test were negatives.

As reaction was several years before, we performed second work up 1 month later.

**Results:** Total IgE 270 KU/l, Specific IgE with penicillin determinants were negative.

Skin tests show positive results only to CLV (20 mg/ml IDT).

DPT was positive to AX, 30 min after first dose (125 mg) patient developed pruritus, facial erythema and cutaneous rash in thorax and back.

DPT with BP following 3 days domiciliary intake was negative.

Second work up.

Skin tests show positive results only to CLV 20 mg/ml IDT.

DPT and with BP following 3 days domiciliary intake was negative again.

**How this report contributes to current knowledge**

Conclusions

We present a double selective sensitization to clavulanic acid and amoxicillin with tolerance to BP.

We confirm that tolerance to BP is a stable phenomenon performing a second BP administration 1 month later, according recently studies, where selective patients tolerate subsequent administrations of other BL.

Our exceptional case highlights the need to be aware of new patterns of beta-lactams sensitization.

We recommend using AX and CLV separately for skin test.

**Consent:** Written informed consent was obtained from the patient for publication of this abstract and any accompanying images.

### P111 Infliximab-specific T cells are detectable also in treated patients who have not developed anti-drug antibodies

#### Alessandra Vultaggio^1^, Francesca Nencini^2^, Sara Pratesi^2^, Andrea Matucci^1^, Enrico Maggi^2^

##### ^1^AOU careggi, Florence, Italy; ^2^Dept of Internal Medicine, Florence, Italy

**Correspondence:** Alessandra Vultaggio

*Clinical and Translational Allergy* 2016, **6(Suppl 3)**:P111

**Background:** Infliximab (IFX) carries potential risk of immunogenicity, with the expansion of memory T cells and the production of anti-drug antibodies (ADA). Little is known about the possibility that sensitization to IFX may occur also in treated ADA− patients. This study was aimed to evaluate whether IFX may be immunogenic in the latter group of patients.

**Materials and methods:** Seventy-one IFX-treated patients, including both ADA+ and ADA− patients were enrolled. Untreated patients and healthy donors were also selected as controls. Memory T cells was evaluated by the proliferation of drug-stimulated PBMC or co-cultures of IFX-pulsed DC plus autologous CD4+ T cells. Measurement of drug-induced cytokines production was performed in the culture supernatants by ELISA. Cytokines mRNA expression was also studied.

**Results:** T cell proliferation and cytokine production were mainly detectable in ADA+ patients, but cytokines were observed in about 25 % of non-proliferating cultures as well. IL-10 was the most frequently detectable cytokine. Additionally, the proportion of patients expressing IL-10 mRNA in IFX-stimulated PBMC was significantly higher in ADA− than in ADA+ patients. IL-10 mRNA levels resulted higher in ADA− than in ADA+ patients. Anti-IL-10 mAbs were able to significantly increase both IFX-driven proliferation and expression of T cell-related cytokines in PBMC.

**Conclusions:** Cell sensitization to IFX may be detectable in a proportion of ADA− treated patients. Proliferation assay combined with cytokines evaluation may work better than the single test in evaluating cell sensitization. In ADA− patients IFX-induced IL-10 may overcome the effects of other T cell-related cytokines.

## Poster Walk 13: Biologicals, local anesthetics, others (P112–P118)

### P112 A case report of allergic immediate systemic reaction to adalimumab and certolizumab

#### Ceyda Tunakan Dalgiç, Fatma Düsünür Günsen, Gökten Bulut, Fatma Ömür Ardeniz, Okan Gülbahar, Emine Nihal Mete Gökmen, Aytül Zerrin Sin, Ali Kokuludag

##### Ege University Medical Faculty, Department of Allergy and Clinical Immunology, Izmir, Turkey

**Correspondence:** Ceyda Tunakan Dalgiç

*Clinical and Translational Allergy* 2016, **6(Suppl 3)**:P112

**Background:** Adalimumab, recombinant fully humanized anti-tumor necrosis factor (anti-TNF) antibody, had been using for the patients with inflammatory artritis and bowel diseases. Adverse reactions to adalimumab are limited mainly to injection site, immediate systemic reactions are very rare. Generalise skin reactions to this antibody are around 1 %. Certolizumab is humanized from mouse, anti-TNF recombinant antibody with the same indications. Systemic adverse reactions to certolizumab is very rare, also.

**Report:** We report a 59-year-old woman with spondylartritisi (SpA) who was treated with adalimumab every 2 weeks for 4 years. At the 4th year, adalimumab was changed to prefilled ready to use form. 30 min after the first application of this form, local reaction and also vomiting, dispnea, laryngeal edema, hipotension with generalise itching happened. Than adalimumab was stopped. Therapy was changed to certolizumab pegol. 30 min after the first injection of certolizumab, she had nause, dizziness and visual disturbances without skin reactions. 15 days later she injected the second certolizumab dosage and the same reactions repeated. She was referred to allergy clinic. Skin prick test with 50 mg/ml of adalimumab was found negative. Intradermal test with (5 mg/ml) 1/10 dilusion was 10/45 mm and 1/100 dilusion was 8/40 mm (histamin intradermal: 10/50 mm). Both skin prick and intradermal tests with certolizumab pegol were negative. Depending on the uncertainty of her history about certolizumab, drug provocation test was administered with total therapy dose. No adverse or allergic reaction occured during provocation. The patient was continued certolizumab therapy seamlessly.

**How this report contributes to current knowledge:** Although adverse systemic reactions to adalimumab are rare, we describe a immediate systemic reaction to adalimumab; fully humanized recombinant biological. We thought the reaction was not due to the new form, because the contents are completely same. Allergic reaction after changing the form seems to be coinsidental. Alternative monoclonal antibodies should be searched for those patients.

**Consent:** Written informed consent was obtained from the patient for publication of this case report and any accompanying images.

### P113 Allergy to local anesthetics: negative predictive value of skin tests

#### Ivana Cegec, Danica Juricic Nahal, Viktorija Erdeljic Turk, Matea Radacic Aumiler, Ksenija Makar Ausperger, Iva Kraljickovic, Iveta Simic

##### University Hospital Zagreb, Zagreb, Croatia

**Correspondence:** Ivana Cegec

*Clinical and Translational Allergy* 2016, **6(Suppl 3)**:P113

**Background:** Although true allergy to local anesthetics (LA) is rare, patients often report unwanted reactions after the administration of LA, requiring allergy consultations. Diagnosis of hypersensitivity reaction to LA is made after skin tests followed by exposition tests. The aim of this study was to determine the negative predictive value (NPV) of skin tests to LA.

**Materials and methods:** A retrospective chart review was performed on patients undergoing local anesthetic skin testing. A total of 50 patients tested for hypersensitivity to LA in the period from January to August 2015 at the Division of Clinical pharmacology at the University Hospital Zagreb were enrolled in this study. All patients underwent skin tests (‘prick’ and intradermal tests with 1:100 dilution) followed by incremental subcutaneous challenge with undiluted LA. Skin tests were performed with lidocaine, articaine, bupivacaine or articaine + adrenaline, while exposition was performed using mainly lidocaine. Telephone follow-up visits were performed during December 2015. On the basis of the follow-up results, the NPV of skin tests was calculated.

**Results:** Skin tests were performed using two or more LA (lidocaine in 50 patients, articaine in 35 patients, bupivacaine in 27 patients, articaine + adrenaline in 11 patients). All skin tests were negative. Subcutaneous challenge was performed with lidocaine in 41 pts, articaine in 7 pts, bupivacaine in 1 pt, and articaine + adrenaline in 1 pt. All patients had negative subcutaneous challenge. In the follow-up period 20 pts received LA, 20 pts have not received LA while 10 pts were unavailable for follow-up. Of the patients who received LA after testing, 19 had no reactions during exposure while 1 pt experienced a severe hypertensive reaction with generalized exanthema. The NPV of skin tests to LA was 95 %. NPV for subcutaneous challenge tests was not calculated due to incomplete data on specific LAs used in subsequent exposures.

**Conclusions:** Skin tests to LA are safe and have an excellent negative predictive value which allows the selection of a local anesthetic that can be safely administered in the future.

**Keywords:** Local anesthetics; Skin test; Hypersensitivity; Negative predictive value

### P114 Cutaneous adverse reactions of molecular targeted agents: a retrospective analysis in 150 patients in our department

#### Yukie Yamaguchi, Tomoya Watanabe, Megumi Satoh, Tomohiko Tanegashima, Kayoko Oda, Hidefumi Wada, Michiko Aihara

##### Yokohama City University Graduate School of Medicine, Yokohama, Japan

**Correspondence:** Yukie Yamaguchi

*Clinical and Translational Allergy* 2016, **6(Suppl 3)**:P114

**Background:** Molecular targeted agents are widely used in various inflammatory and immune diseases and tumors. Differently from adverse reactions induced by conventional drugs, these drugs demonstrate various new types of adverse reactions. Some of events may be induced by cross-reactions related to the expression of the same antigen on different tissue. Immune imbalance caused by a depletion of specific antigen may be a trigger of unpredictable reactions. It is important to know the prevalence, clinical types, and severity of the side-effects in each drugs.

**Materials and methods:** We retrospectively analyzed patients who visited our department due to cutaneous adverse reactions caused by molecular targeted agents from 2010 to 2015. Types of drugs, clinical features of cutaneous adverse reactions, and subsequent outcome were evaluated.

**Results:** One hundred and fifty patients were evaluated in this study. High frequency agents were EGFR inhibitors (63 %), multikinase inhibitors (8 %), TNF-a inhibitors (6 %), and programmed death-1 (PD-1) inhibitors (5 %). Regarding clinical phenotypes, acneiform eruptions and perionychia were highly observed (68 %) mainly caused by EGFR inhibitors as the target antigen EGFR is also involved in skin homeostasis. Maculopapular drug eruption (10 %) and hand-foot syndrome (7 %) were observed in users of multikinases inhibitors and EGFR inhibitors. Of note, systemic drug eruption (erythroderma, erythema multiforme, maculopapular) was highly observed in patients treated with multikinase inhibitors. Psoriasis-like and palmoplantar pustulosis-like eruptions (5 %) were caused by TNF-a inhibitors and IL-6R inhibitors. Although the frequency was low, lupus-like reaction, vitiligo, and other unexpected reactions were caused by inhibitors of TNF-a or PD-1, supposedly those inhibitors lead to immune imbalance further causing autoimmunity and inflammatory reactions. As for the outcome, 85 % of patients continued molecular targeted agents, while 15 % of patients required discontinuation of the drug.

**Conclusions:** Predicteable cutaneous reaction, like acneiform eruptions by EGFR inhibitors, would be possible to manage without discontinuation of the drug. Some drugs, such as immune checkpoint inhibitors, may cause unexpected immune responses. Since adverse reactions of molecular targeted agents are variable depending on drugs. It is important to recognize the function of targeted antigen, and close monitoring is recommended.

**Keywords:** Molecular targeted agents; Biologics

### P115 Generalized paralysis induced by local lidocaine injection

#### Jaechun Jason Lee, Jay Chol Choi, Hwa Young Lee

##### Jeju National University School of Medicine, Jeju, South Korea

**Correspondence:** Jaechun Jason Lee

*Clinical and Translational Allergy* 2016, **6(Suppl 3)**:P115

**Background:** Local anesthetics, such as lidocaine, are widely used for numbing. Adverse drug reactions related to lidocaine are variable, unpredictable, and rarely reproducible, with the exception of some typical cases.

**Report:** A 42-year-old female who had shown a bizarre neurological reaction after lidocaine injection for dental procedures was referred for diagnosis and safe anesthetic alternatives. Within a few minutes after exposure to lidocaine, she was unable to move any extremity, or to speak, while sensory and high cranial nerve functions were preserved. She was alert and able to communicate with eye blinks. This reaction was repeatedly reproduced after intradermal injection of 2 % lidocaine, with complete recovery within 1 h without treatment. No cross-reactivity with mepivacaine and bupivacaine was observed.

**How this report contributes to current knowledge:** This is the first report of immediate and transient generalized paralysis related to lidocaine.

**Consent:** Written informed consent was obtained from the patient for publication of this abstract and any accompanying images.

### P116 Hypersensitivity to local anaesthetics: a 10 year review

#### Rosa-Anita Rodrigues Fernandes^1^, Emília Faria^1^, Joana Pita^1^, Nuno Sousa^2^, Carmelita Ribeiro^3^, Isabel Carrapatoso^1^, Ana Todo Bom^1^

##### ^1^Allergy and Clinical Immunology Department, Coimbra University Hospital Center, Coimbra, Portugal; ^2^Allergy and Clinical Immunology Consult, Leiria Hospital Center, Leiria, Portugal; ^3^Coimbra University Hospital Centre, Coimbra, Portugal

**Correspondence:** Rosa-Anita Rodrigues Fernandes

*Clinical and Translational Allergy* 2016, **6(Suppl 3)**:P116

**Background:** True allergy to local anaesthetics (LA) is uncommon. Allergic reactions account for <1 % of all adverse reactions. Immediate reactions are rare. The aim of this study was to describe the main characteristics of the population with confirmed LA allergy among patients referred to our outpatient clinic for suspected hypersensitivity (HS) to LA.

**Materials and methods:** Retrospective analysis of the medical files of patients with suggestive history of immediate HS reactions to LA, referred to our outpatient centre between January 2006 and December 2015. In order to confirm the diagnosis, we conducted skin prick tests and subcutaneous drug provocation test (DPT) with the undiluted commercial solution of the culprit or an alternative LA. DPT was performed in increasing doses, every 15 min, up to a cumulative dose of 3.6 ml. Sensitization to latex was excluded.

**Results:** A total of 65 patients with history of an adverse reaction to LA underwent DPT, 75 % female with a median age of 44 years (±17.4). Only 5 patients (7.7 %) had positive DPT (lidocaine in 2 patients, mepivacaine in 2 patients and ropivacaine in 1 patient). Of those patients, 60 % were female and the median age was 35 years (±20.6 years). Sixty percent of the patients had past history of multiple surgical interventions (with general anesthesia) and allergic diseases, asthma and rhinitis being the most frequent. Cutaneous symptoms (urticaria and angioedema) were the main manifestation, in 4 patients (66 %). There was no registry of severe adverse reactions. All reactions started within 2 h following LA application and regressed spontaneously without any treatment. Sensitization to both lidocaine and mepivacaine was determined in 2 patients. Comorbidities such as hypertension (2 patients), diabetes (1 patient) and thyroid disease (1 patient) were also seen. Eighty-three percent of these reactions occurred during dental procedures and 60 % of these after administration of lidocaine.

**Conclusions:** As stated in other studies, we find a low positivity rate to DPT. And our findings were similar to those found in the literature: adverse reactions to LA are more prevalent in the mid-age and with cutaneous symptoms being the most frequent manifestation of HS. Although rare, consequences of true allergy to local anaesthetics can be serious, considering a patient’s future management and therapy.

**Keywords:** Local anaesthetics; Hypersensitivity

### P117 Local anaesthetics: a rare culprit in hypersensitivity reactions

#### Ana Rodolfo, Eunice Dias-Castro, Josefina Cernadas

##### Centro Hospitalar de São João, Porto, Portugal

**Correspondence:** Ana Rodolfo

*Clinical and Translational Allergy* 2016, **6(Suppl 3)**:P117

**Background:** Local anaesthetics (LA) are widely used drugs with an exceptional benefit-risk profile. Adverse reactions are scarce. Hypersensitivity reactions are especially rare, accounting for <1 % of all adverse reactions. When a reaction occurs, an allergy work-up should be performed in order to exclude an allergic reaction or to identify an alternative drug.

**Materials and methods:** Retrospective analysis of data from patients referred to our drug allergy unit with suspected LA allergy in the past 5 years. The analysis was performed using SPSS, version 21.

**Results:** We studied 47 patients for suspected LA allergy (83 % females, with median age 49, and interquartile range 23). Six patients were atopic. Thirty-six percent of patients were referred by their family physician and 26 % by dentists.

Of the 47 patients, 38 % had a suspected reaction during a dental procedure, 14 % had positive patch tests with LA and 13 % had per-operative anaphylaxis. Considering the patients who had a suspected reaction, 74 % had immediate reactions. In 49 % of these cases, there was mucocutaneous involvement, 33 % had respiratory symptoms and 33 % vasovagal response. The LA used was known in 70 % of these cases, none of them were an ester. Lidocaine was the culprit in 20 cases, mepivacaine in 5, articaine in 5 and ropivacaine in 4.

Skin prick tests (SPT) and intradermal tests (ID) were performed with validated concentrations, without vasoconstrictor. Only 2 patients had positive tests: 1 positive SPT to mepivacaine and one positive ID to procaine; the other patient had a positive ID to ropivacaine. All 45 patients with negative skin tests were submitted to subcutaneous challenges that were negative.

None of the patients referred because of positive patch tests to LA had a positive study.

**Conclusions:** The majority of adverse reactions to LA may be attributed to non-immune mediated mechanisms, pharmacologic, psycho or neuro-vegetative reactions. When LA are used, the patients are usually under stress. Our experience is in agreement with literature since hypersensitivity was excluded in the majority of the patients with suspected LA reactions.

**Keywords:** Local anaesthetics; Local anaesthetics hypersensitivity

### P118 Stevens–Johnson syndrome in clinical practice: a variant of clinical course

#### Marina Voronova

##### State Hospital 52, Moscow, Russia

**Correspondence:** Marina Voronova

*Clinical and Translational Allergy* 2016, **6(Suppl 3)**:P118

**Background:** A 44 year-man was urgently admitted to our allergy clinic with complaints of pain in the area of scrotum, painfulness in oral cavity, rash on hands, eyelids and foot. Anamnesis excerption: Alcohol abuse for a long time. About 2 years ago, following one of patient’s drinking-bouts, episode with loss of consciousness emerged, qualified as secondary epilepsy; carbamazepine was administered. The patient described vesicular rash on lips and hands following the carbamazepine medication; he did not seek medical attention, though. The therapy was cancelled. Within 2 weeks the skin rash passed. The present episode had also been related with a prolonged period of alcohol ingestion, whereupon a detoxification treatment was administered, and, taking into account the possibility of episyndrome, carbamazepine was ordered, too. On the second day vesicular rash appeared on hands, thereupon in the scrotum area, on eyelids and on mucous tunic of mouth, forcing the patient to seek medical attention. Condition on admittance: of moderate gravity. Rash on hands with big serous fluid-filled vesicles; negative Nikolsky symptom. There were erosion with diapedetic bleeding and scrotal oedema in the area of scrotum and penis. Cheilitis, multiple aphthae on the internal surface of cheeks, on hard and soft palate and on tong were revealed, as well as haemorrhagic crustings on eyelids, moderate injection in scleral vessels. Physical state: with no specific findings. Systemic glucocorticoids and antihistamine preparations were administrated, as well as antibacterial, infusion therapy and topical treatment. On the second day a total relief of diapedetic bleeding in the scrotal area was achieved. To the end of the 10th day a total epithelization in the area of penis and scrotum occurred. Aphtae on hard and soft palate relieved totally within the first 3 days. Laboratory results have shown increasing levels in circulating thrombocytes according to the relief of diapedetic bleeding. Maximal levels were attained by the 6th day (667*109/l) with the following return to normal values. On admission C-reactive protein levels were elevated up to 136.65 mg/l, by the 7th day they returned to normal values.

No eosinophilia or leukocytosis had been revealed. The described case represents a favorable variant of clinical course of carbamazepine intolerance, with a quick epithelisation, and no injury in liver, kidneys or blood islands.

**Keywords:** Stevens–Johnson syndrome; Clinical case

**Consent:** Written informed consent was obtained from the patient for publication of this abstract and any accompanying images.

## Poster Walk 14: RCM (P119–P128)

### P119 13 cases of severe anaphylactic reactions due to radiocontrast media

#### Jaume Martí Garrido, Ramon Lopez Salgueiro, Diana Kury Valle, Verónica Pacheco Coronel, Carolina Perales Chordá, Dolores Hernandez Fernandez De Rojas

##### IIS La Fe, Valencia, Spain

**Correspondence:** Jaume Martí Garrido

*Clinical and Translational Allergy* 2016, **6(Suppl 3)**:P119

**Background:** The increasing use of radiocontrast media (RCM) is associated with an increase in adverse reactions with these preparations.

**Materials and methods:** We revised a total of 249 cases referred during 2012–2014 for allergic evaluation due to adverse with RCM. We collected information about the radiological procedures, adverse reactions and results of allergy study of patients who developed severe anaphylactic reactions.

**Results:** We analysed 5 men and 8 women with an average age of 63 years (37–86). In 69 % respiratory symptoms were reported, 62 % neurological, 54 % cutaneous, 31 % cardiovascular and 31 % digestive symptoms. 31 % complaint of subjective symptoms (general discomfort, heat or flushing). The reaction was immediate (within minutes) in 69 % of cases. One patient required admission to the intensive care unit and another had a repeated episode of anaphylaxis after readministration of RCM despite of the premedication. In 4 cases the reaction occurred during a computed tomography (CT), in 4 with intravenous urography (IVU), and 3 with magnetic resonance (MR). In 2 cases the radiological procedure was not specified. In 2 of the 13 patients (15 %), intradermal tests were positive with the RCM involved: iomeprol and dimeglumine gadopentetate. The basophil activation test (BAT) with the RCM was positive in 4 cases: 2 with iomeprol and iodixanol, 1 with iopamidol and 1 with gadobenate and gadopentate.

**Conclusions:** TC and IVU were the most frequently involved radiological procedures. Respiratory symptoms were the most common clinical manifestations. Skin tests and BAT helped to identify the agents responsible for the reactions. The sensitivity and specificity of these tests are still undetermined.

### P120 Anaphylactic shock after administration of iodinated contrast medium during cardiac catheterization

#### Roselle Catherine Yu Madamba, Marta Ferrer, Maria Jose Goikoetxea, Carmen D’Amelio, Amalia Bernad, Olga Vega, Gabriel Gastaminza

##### Clinica Universidad de Navarra, Pamplona, Spain

**Correspondence:** Amalia Bernad

*Clinical and Translational Allergy* 2016, **6(Suppl 3)**:P120

**Background:** Iodinated contrast media (ICM) are one of the most commonly used in the field of diagnostic medicine today. Though considered generally safe, they can cause hypersensitivity reactions that can either be immediate or non-immediate. Anaphylactic shock reactions to ICM are rare but serious and could be life threatening. Hence, urgent and timely treatment should be administered.

**Report:** We present a case of a 64 year old hypertensive patient with ischemic cardiopathy who presented with intense cephalic heat, tachycardia, hypoxemia and hypotension of 50/30 mmHg without any cutaneous lesions immediately after the administration of 35 g of Iomeprol contrast medium for cardiac catheterization. Epinephrine IM, Dexchlorpheneramine and corticosteroid IV and intravenous fluid therapy were administered which afforded the reversal of the hypotension. The tryptase at the time of the reaction and 2 h post reaction were both elevated, 85.70 and 88.70 μg/l respectively. Baseline tryptase was normal (6.86 μg/l). Skin prick test and basophil activation test to different contrast media were negative while intradermal test to Iomeprol showed a positive result and negative to Iohexol, Iodixanol and Meglumine. Thus, challenge test to Iodixanol reaching a total dose of 47 g as an alternative medium was done which he tolerated well. One month later, the patient underwent another cardiac catheterization. However, 10 min after the administration of 15 g of Iodixanol, he presented another episode of anaphylactic shock. He was given the same medications he had received previously which reversed the anaphylactic shock. Tryptase level was positive (30.20 μg/l). Due to the episode of anaphylactic shock, the contemplated cardiac catheterization was discontinued. Skin tests the next day resulted positive to Iohexol, Iodixanol and Iomeprol.

**How this report contributes to current knowledge:** Despite that the patient tolerated well the challenge test with Iodixanol and skin test was negative, possible hypersensitivity reactions can still occur. Although challenge test is considered the gold standard in drug allergy, it can also provoke sensitisation to the drug or increase the sensitivity that could not be detected prior due to cross reaction. As of the present, the pathophysiological mechanism of these allergic reactions still needs to be exemplified.

**Consent:** Written informed consent was obtained from the patient for publication of this abstract and any accompanying images.

### P121 Anaphylactic shock and cardiac arrest induced by gadolinium-based contrast agents

#### Beatriz Pola Bibián, Marina Lluncor Salazar, Gemma Vilà Nadal, Ana María Fiandor Roman, Javier Dominguez Ortega, Miguel Gonzalez Muñoz, Santiago Quirce Gancedo, Maria Rosario Cabañas Moreno

##### La Paz Hospital, Madrid, Spain

**Correspondence:** Beatriz Pola Bibián

*Clinical and Translational Allergy* 2016, **6(Suppl 3)**:P121

**Background:** Gadobenate Dimeglumine and Gadoteric Acid are two gadolinium-based contrast agents that enhance the contrast images obtained by magnetic resonance imaging. Allergic reactions to them are rare.

**Materials and methods:** We report two cases of IgE-mediated anaphylactic shock, induced by these gadolinium-based contrast agents and documented by positive allergy assessment. The first case is a 26-year-old woman with no previous drug allergy who developed dizziness, generalised erythrodermia and throat tightness, when a nuclear magnetic resonance with MultiHance (Gadobenate Dimeglumine) was being performed. On physical examination, she had hypotension, tachycardia, tachypnea, 69 % of basal oxygen saturation and generalized pulmonary hypoventilation. She recovered after treatment with corticosteroids and adrenaline. Nine months later the patient was referred to the allergy unit. The second case is a 51-year-old woman with a history of high blood pressure treated with betablockers, rhinoconjunctivitis and asthma due to sensitization to house dust mites and dog dander. Immediately after the intravenous infusion of Dotarem (Gadoteric Acid) she developed dizziness, loss of consciousness and cardiac arrest. On physical examination, she had universal rash without wheals, 90 % of basal oxygen saturation and generalized wheezing. CPR was started and 1 mg of Adrenaline was administrated. She recovered after 15 min and was admitted to the ICU. Two months later she was referred to the allergy unit.

**Results:** Case 1: Basal serum tryptase was normal. Skin prick tests (SPTs) were positive with Gadobenate Dimeglumine and negative with other paramagnetic contrasts agents and latex. The patient tolerated an MRI with Gadobutrol 2 years later. Case 2: Basal serum tryptase was normal. Basophil activation test (BAT) was positive with Gadoteric Acid, and negative with other paramagnetic and iodinated contrast agents.

**Conclusions:** We report two cases of an anaphylactic shock, one with cardiac arrest, immediately after the intravenous infusion of a paramagnetic contrast agent. Positive SPTs to Gadobenate Dimeglumine and positive BAT to Gadoteric Acid suggest an IgE-mediated mechanism. Hypersensitivity reactions with paramagnetic contrasts are very unusual. There are few cases with positive SPTs or BAT reported in literature. We should take into account that SPTs and BAT can be helpful to identify the responsible agent and the alternative contrasts that could be administered safely to the patient in the future.

**Keywords:** Paramagnetic contrast agents; Anaphylaxis

**Consent:** Written informed consent was obtained from the patient for publication of this abstract and any accompanying images.

### P122 Anaphylaxis to gadobenate and cross-reactivity to other gadolinium-based contrast agents in two patients

#### Kathrin Scherer Hofmeier

##### Allergy Unit, Department of Dermatology, University Hospital Basel, Basel, Switzerland

**Correspondence:** Kathrin Scherer Hofmeier

*Clinical and Translational Allergy* 2016, **6(Suppl 3)**:P122

**Background:** The frequency of adverse reactions to gadolinium-based contrast media is estimated to be 0.07–2.4 %. True allergic hypersensitivity reactions are very rare, presenting usually as urticarial exanthems of minor severity. The reported incidence is 0.004–0.007 %. Severe life-threatening reactions were extremely rare (0.001–0.01 %). Prior allergic reactions to gadolinium contrast media (GCM) are considered risk factors for new sensitizations to other GCM. We report on two female patients with severe immediate type reactions to Gadobenate, with cross reactivity to other GCM.

**Materials and methods:** Patient 1 (48 years): An MRI with Gadobenate was performed for diagnosis of pancreatitis. Immediately after application of the GCM she developed generalized itch and erythema, dyspnoea and cardiac arrest after 10 min. Mechanical and drug resuscitation was successfully performed. No definite prior exposure.

Patient 2 (74 years): MRI with Gadobenate was performed for cardiac diagnostics. Within few minutes after application of the drug she developed a generalized sensitization of head, erythema, dysphagia, dyspnoea and swelling of the tongue. Prior exposure to an unknown GCM.

**Results:** Pricktests were negative for Gadoterate, Gadobutrol, Gadoxetate and Gadopentetat in both patients. Gadobenate was postive in patient 2. Intradermaltests were positive for Gadobenate and Gadobutrol in both patients, and Gadoxetate, Gadoterat and Gadopentetat in patient 1. Mastcell tryptase and Basophil activation tests were normal/negative in both patients.

**Conclusions:** We report on two cases of rare severe hypersensitivity reactions to Gadobenate with skin test cross-reactivity to Gadobutrol in both patients, and further GCM in one patient. Gadopentetat, a linear non-ionic GCM, was skin test negative in both patients, whereas the linear ionic culprit Gadobenate was cross-reactive with the cyclic Gadobutrol in both patients, and cross-reactive to other linear ionic and cyclic GCM in patient 1.

**Keywords:** Anaphylaxis; Gadolinium; Gadobenate

**Consent:** Written informed consent was obtained from the patient for publication of this abstract and any accompanying images.

### P123 Anaphylaxis to glatiramer acetate in a patient with multiple sclerosis

#### Fabrícia Carolino^1^, Vladyslava Barzylovych^2^, Josefina R. Cernadas^1^

##### ^1^Serviço de Imunoalergologia, Centro Hospitalar São João E.P.E., Porto, Portugal; ^2^Serviço de Imunoalergologia, Centro Hospitalar São João E.P.E., Porto, Portugal; Drug Allergy Diagnostics Centre, Institute of Paediatrics, Obstetrics and Gynaecology of NAMS, Kiev, Ukraine

**Correspondence:** Fabrícia Carolino

*Clinical and Translational Allergy* 2016, **6(Suppl 3)**:P123

**Background:** Glatiramer acetate (GA) or Copaxone^®^ (marketed by Teva Pharmaceuticals Europe B. V.) is an injectable immunomodulatory treatment approved for relapsing-remitting multiple sclerosis (MS) in preventing relapses and slowing the disability progression. Rare cases of hypersensitivity reactions to this drug have been published in the literature. The authors describe a case of systemic symptoms associated with GA and the diagnostic workup of the patient.

**Report:** The authors report the case of a 34 year-old woman diagnosed with MS in 2008, initially treated with interferon-β1a (Avonex^®^). As relapses were frequent despite treatment, this was changed in January/2013 to natalizumab (Tysabri^®^), which is a monoclonal antibody (α4-integrin antagonist) with similar therapeutic indications to those described for GA. After 12 months of treatment, natalizumab was stopped because the patient developed persistent anti-drug antibodies. Considering that the patient was planning a pregnancy a new treatment (with GA) was started in March/2015. Four months later, the patient presented a generalized skin-burning sensation, dyspnoea and palpitations, 1 min after GA administration with no additional signs or symptoms. At this point, the patient was referred to our Drug Allergy Department for assessment. Skin tests were performed with GA plus the inactive ingredient of Copaxone^®^ with allergenic potential, mannitol; the dilutions were prepared with saline solution. For skin prick tests we used GA 20 mg/ml in the 1/100, 1/10 and 1/1 dilutions, and mannitol 100 mg/ml (undiluted). Intradermal tests were performed with 1/1,000,000–1/10 dilution of GA (20 mg/ml), with a strong positive result at 1/10 concentration. Intradermal test to mannitol (1/10 dilution) was negative.

**How this report contributes to current knowledge:** We also tested three (2 atopic and 1 non-atopic) controls for GA at 1/10 dilution with negative results. These results point to an underlying immunological mechanism in this case. A desensitization approach with GA might be considered for this patient.

**Consent:** Written informed consent was obtained from the patient for publication of this abstract and any accompanying images.

### P124 Delayed hypersensitivity reaction to radiocontrast media

#### Fabrícia Carolino^1^, Diana Silva^2^, Leonor Leão^1^, Josefina R. Cernadas^1^

##### ^1^Serviço de Imunoalergologia, Centro Hospitalar São João E.P.E., Porto, Portugal; ^2^Serviço de Imunoalergologia, Centro Hospitalar São João E.P.E.; Laboratório de Imunologia, Faculdade de Medicina, Universidade do Porto, Porto, Portugal

**Correspondence:** Fabrícia Carolino

*Clinical and Translational Allergy* 2016, **6(Suppl 3)**:P124

**Background:** Iodinated contrast media (ICM) may associate with different types of adverse events including allergic and non-allergic hypersensitivity reactions as defined by the European Academy of Allergy and Clinical Immunology. Immune-mediated reactions are either immediate or non-immediate reactions that become apparent later than 1 h (and up to several days) after exposure and these are less frequently reported.

**Report:** The authors report the case of a 46 year-old woman, with history of Hodgkin’s lymphoma, evaluated in our Drug Allergy Unit for two suspected hypersensitivity reactions to ICM. In 2014, the patient underwent a contrasted CT scan with iomeprol (Iomeron^®^) and 72 h later developed a generalized pruritic micropapular exanthema that resolved completely in a week (with antihistamine and corticosteroid medication). She had been previously exposed to ICM with tolerance. In 2015, a new high-resolution CT scan was performed using the same ICM (iomeprol), and again a diffuse pruritic exanthema developed (6 h later) but this time with facial angioedema and fever (39 °C); the symptoms resolved in 4 days under medication (antihistamine and corticosteroid), leaving residual purplish lesions that eventually disappeared (after weeks). Skin prick tests with idopovidone (Betadine^®^), and undiluted ioversol (Optiray^®^), iobitridol (Xenetix^®^), iopromide (Ultravist^®^) and iomeprol (Iomeron^®^) were negative. Intradermal tests were performed with 1/100 and 1/10 dilutions of ioversol (Optiray^®^), iobitridol (Xenetix^®^), iopromide (Ultravist^®^) and iomeprol (Iomeron^®^); immediate reading was negative for all tested ICM but late reading (at 48 h) was positive to ioversol (Optiray^®^) and iomeprol (Iomeron^®^) in both dilutions.

**How this report contributes to current knowledge:** The results confirmed a delayed hypersensitivity to ICM, with cutaneous cross-reactivity between ioversol and iomeprol.

**Consent:** Written informed consent was obtained from the patient for publication of this abstract and any accompanying images.

### P125 Drug reaction with eosinophilia and systemic symptoms induced by iodixanol

#### Gemma Vilà-Nadal^1^, Beatriz Pola^1^, Marina Lluncor^1^, Ana Fiandor^2^, Teresa Bellón^3^, Javier Domínguez^2^, Santiago Quirce^2^

##### ^1^Allergy Department. La Paz Hospital Institute for Health Research (IdiPAZ), Madrid, Spain; ^2^Allergy Department. La Paz Hospital Institute for Health Research (IdiPAZ), Consorcio Piel en RED, Madrid, Spain; ^3^Immunology Department. La Paz Hospital Institute for Health Research (IdiPAZ), Consorcio Piel en RED, Madrid, Spain

**Correspondence:** Gemma Vilà-Nadal

*Clinical and Translational Allergy* 2016, **6(Suppl 3)**:P125

**Background:** We describe a case of a Drug Reaction with Eosinophilia and Systemic Symptoms (DRESS) following a coronary catheterization with iodixanol. Late reactions to iodinated contrast media are frequent. We describe the tests used for the diagnosis in this case.

**Report**

**Method:** We report a case of a 74-year-old man admitted to the coronary unit with cardiac arrest. A cardiac catheterization was performed without complications. Three days later he received another cardiac catheterization and within 24 h he presented a generalized maculopapular exanthema with pruritus and facial angioedema. Over the next few days the body temperature rose up to 38.5 °C. Blood tests showed eosinophilia (1200/mcL), total white blood cell count 19000/mcL, neutrophils 16,700/mcL, renal impairment (creatinine 1.89 mg/dl, previously being normal). The contrast used for catheterization was iodixanol. The patient also received ticagrelor, carvedilol, enalapril, furosemide, fondaparinux and lorazepam. Assessment of causality was established using the Spanish Pharmacovigilance System Algorithm. Results were (+6) “probable” for iodixanol and (+4) “possible” for carvedilol and ticagrelor. A multidisciplinary group composed by a dermatologist; a pharmacologist and an allergist evaluated the patient. This case was included in the Piel en RED registry. Six months after the reaction the patient was referred to the allergy unit and tests were performed. The allergy study included epicutaneous tests and the lymphocyte transformation test (LTT) according to Pichler et al. An stimulation index (SI) was calculated.

**Results**: The diagnosis of a “probable” DRESS was established according to the scoring system described by Kardaun et al. Our patient received a score of 4. A skin biopsy showed spongiotic dermatitis with eosinophils. The patient had a positive LTT to iodixanol; the SI was over 8 in two concentrations. LTT showed also positive results for other iodinated contrast media. We also tested carvedilol and ticagrelor with a lightly positive result. Epicutaneous test showed remarkable positivity to iodixanol beginning at the 48 h reading. The test was negative to carvedilol, iobitridol and iomeprol.

**How this report contributes to current knowledge:** LTT and patch test were useful to identify this agent as responsible for the reaction. DRESS requires a multidisciplinary approach and an allergy study is essential to determine the etiology of the disease.

**Consent:** Written informed consent was obtained from the patient for publication of this abstract and any accompanying images.

### P126 Electronic consultation support system for radiocontrast media hypersensitivity changes clinician’s behavior

#### Min-Suk Yang^1^, Sun-Sin Kim^2^, Sae-Hoon Kim^3^, Hye-Ryun Kang^4^, Heung-Woo Park^4^, Sang-Heon Cho^5^, Kyung-Up Min^5^, Yoon-Seok Chang^3^

##### ^1^Seoul National University Borame Hospital, Seoul, South Korea; ^2^Seoul National University Hosptial Healthcare System Gangnam Center, Seoul, South Korea; ^3^Seoul National University Bundang Hospital, Seongnam, South Korea; ^4^Seoul National University Hosptial, Seoul, South Korea; ^5^Seoul National University College of Medicine, Seoul, South Korea

**Correspondence:** Yoon-Seok Chang

*Clinical and Translational Allergy* 2016, **6(Suppl 3)**:P126

**Background:** Patients with a previous history of radiocontrast media (RCM) hypersensitivity could be overlooked, resulting in repeated reactions. To avoid this, ‘consultation support system for RCM hypersensitivity’ has been embedded to Seoul National University Bundang Hospital (SNUBH) since Dec 2011. We analyzed the influence of this system on doctors’ practice pattern.

**Materials and methods:** A retrospective study was conducted with the patients with previous RCM reactions (Dec 1st 2010–Nov 30th 2012). Control period was between Dec 2010 and Nov 2011 and intervention period was Dec 2011 and Nov 2012. Primary outcome was composite outcome of premedication and consultation. Premedication was defined as the preventive medication prescribed by the doctor who ordered RCM enhanced CT (CT) at the same time. Secondary outcomes were the recurrence rate after the consultation support system and ther rate of premedication using systemic steroid for those with previous history of moderate to severe RCM reactions.

**Results:** In the control period, 189 clinicians prescribed 913 CT scans and 225 clinicians did 1.153 examinations in the intervention period. The odds of achieving composite outcome increased significantly after the consultation support system (OR 1.54, 95 % CI 1.15–2.05). Clinicians in both medical (OR 1.48, 95 % CI 1.06–2.07) and surgical (OR 2.07, CI 1.24–3.46) part showed significant changes in their behavior, but emergency department did not (OR 1.07, 95 % CI 0.41–2.78). Professors (OR 1.47, 95 % CI 1.06–2.04) and trainees (OR 1.97, 95 % CI 1.22–3.18) showed significant changes in their behavior to the patients with previous RCM reactions. The rate of premedication using systemic steroid for the patients with previous history of moderate to severe RCM reactions did not changed significantly (OR 1.68, 95 % CI 0.70–0.404). The results of analyses with 86 clinicians who ordered CT scans during both control and intervention periods were not altered.

**Conclusions:** The consultation support system for those with previous RCM hypersensitivity reactions changed doctor’s practice patterns and decreased recurrent RCM hypersensitivity reactions as well.

**Keywords:** Drug hypersensitivity; Radiocontrast media; Clinical decision supporting system

### P127 Hypersensitivity reactions to iodinated contrast media: skin testing and follow-up

#### Danica Juricic Nahal, Ivana Cegec, Viktorija Erdeljic Turk, Iva Kraljickovic, Matea Radacic Aumiler, Ksenija Makar Ausperger, Iveta Simic

##### University Hospital Zagreb, Zagreb, Croatia

**Correspondence:** Danica Juricic Nahal

*Clinical and Translational Allergy* 2016, **6(Suppl 3)**:P127

**Background:** Iodinated contrast media (ICM) are frequently used to enhance radiological procedures. Hypersensitivity reactions to ICM can be classified as immediate or nonimmediate. Skin tests have been performed in the diagnosis of both types of reactions to ICM. The sensitivity of skin tests has not been adequately validated.

**Materials and methods:** A retrospective chart review was performed on patients (pts) undergoing ICM skin testing. A total of 45 pts tested for ICM allergy at the Division of Clinical Pharmacology at the University Hospital Zagreb between July 2014 and October 2015 were enrolled. All pts underwent skin tests (prick test and intradermal test with immediate and delayed reading) to iohexole and/or iodixanole. Pts were contacted during December 2015 and questioned about exposure to ICM after testing. Eligible pts (n = 40) were classified into three groups: nonimmediate or immediate hypersensitivity reaction to ICM, and previously unexposed group.

**Results:** The nonimmediate group comprised 15 pts with hystory of exanthema (53.3 %), urticaria (20 %), angioedema (13.3 %) and pruritus (6.7 %) to ICM. One pt had positive skin tests to iodixanole and negative to iohexole. All other performed tests were negative. In the follow-up period 4 pts were exposed to ICM without adverse reactions; 10 pts were not exposed to ICM; 1 pt was unavailable for follow up.

The immediate group comprised 11 pts with hystory of gastrointestinal symptoms (27.3 %), urticaria (27.3 %), angioedema (18.2 %), general symptoms (18.2 %) and flush (9.1 %) to ICM. One pt tested positive to iohexole. All other performed tests were negative. In the follow-up period 7 pts were exposed to ICM; 6 pts experienced no adverse reactions; 4 pts were not exposed to ICM and 1 pt developed angioedema.

The previously unexposed group comprised 14 pts. All skin tests were negative. The reason for referral to ICM testing was a hystory of serious allergic reaction to different allergens. In the follow-up period 5 pts were exposed to ICM without adverse reactions; 4 pts were not exposed to ICM; 5 pts were not available for follow up.

**Conclusions:** Overall, 16 pts were exposed to ICM in the follow-up period and of those, 1 pt developed an immediate reaction. The calculated negative predictive value in this study is thus 93.8 %.

This study adds further knowledge to the field of ICM hypersensitivity. Studies including follow-up data on sebsequent ICM exposure are needed in order to adequately validate ICM skin tests.

**Keywords:** Iodinated contrast media; Hypersensitivity; Skin test; Follow-up

### P128 Would iodine allergy exist?

#### Clémence Delahaye, Jenny Flabbee, Julie Waton, Olivia Bauvin, Annick Barbaud

##### Dermatology and Allergy department, Brabois hospital, University hospital of Nancy, Lorraine University, 54500 Vandoeuvre-Lès-Nancy, France

**Correspondence:** Annick Barbaud

*Clinical and Translational Allergy* 2016, **6(Suppl 3)**:P128

**Background:** Allergies to povidone iodine (PVI) are related to povidone (PV), those due to radio contrast media (RCM) to cyclic structures and those with seafood related to animal protein such as tropomyosin. The iodine allergy is a mythical entity, but nevertheless!

**Materials and methods:** A 39 year old diabetic man, with end stage renal disease undergoing peritoneal dialysis, was referred for immediate hypersensitivity (IH) reactions. Betadine dermique^®^ containing povidone iodine triggered an immediate contact pruritic rash disappearing in 30 min (mns). Angioedema and urticaria occurred a few mns after ingestion of shrimps, mussels, oysters, whelks, trout and salmon. Awaiting kidney transplant, having never received RCM, a predictive assessment of RCM IH was required. Patch and prick tests (pt), IDT, specific IgE and RCM reBackground were performed under hospital supervision.

**Results:** While all patch tests were negative, pt were positive for pure Betadine dermique^®^ and iodized alcohol at 1 % in water but negative for Lugol 2 % (potassium iodide) and PV. Pt to a native oyster was positive but negative for crab, mussel and shrimp. IgEs were negative for salmon, mussel, oyster and shrimp. Pt and IDR were negative for iobitridol, ioxaglate and iodixanol in pure form (use concentration), but the intravenous administration of 1 ml iobitridol induced a generalized pruritus, a trunk and face erythema with ear edema, occurring after 30 mns and resolution in 60 mns after treatment with intravenous methylprednisolone and antihistamines.

**Conclusions:** This exceptional case of multiple reactivities to iodine products and oyster suggests that for this patient iodine itself could be the common allergen. The positive pt to oyster is probably not related to a tropomyosin sensitization. Unlike previous published cases of allergy to povidone iodine, in this case the IH does not appear due to PV. While IDT was negative, iobitridol induced an immediate reaction. We emphasize (1) in case of PVI IH, the need to do a pt to PVI and PV before concluding to the lack of iodine sensitization and thus allow RCM; (2) if negative IDT with RCM, we must continue investigations by an intravenous Background test with a limited volume of RCM and (3) even if it is exceptional, an iodine allergy may exist. Investigations are continued with in vitro tests.

**Keywords:** Allergy; Iodine; Multiples reactivity

**Consent:** Written informed consent was obtained from the patient for publication of this abstract and any accompanying images.

## Poster Walk 15: MPE/type 4 (P129–P137)

### P129 Delayed hypersensitivity cutaneous reactions: a case/control study from a tunisian database

#### Karim Aouam, Najah Ben Fadhel, Zohra Chadly, Nadia Ben Fredj, Naceur A. Boughattas, Amel Chaabane

##### Faculty of Medicine/University hospital/University of Monastir, Monastir, Tunisia

**Correspondence:** Karim Aouam

*Clinical and Translational Allergy* 2016, **6(Suppl 3)**:P129

**Background:** Drug hypersensitivity reactions represent a heterogeneous clinical entity with diverse pathogenesis and result in a considerable burden of morbidity and mortality. Diagnostic procedures rely on clinical history, skin testing and in some cases, provocation tests. Drug imputability is still difficult to establish due to the weakness of sensitivity of some skin tests and the impossibility to perform provocation test in case of severe reactions. The aim of our study is to evaluate delayed-type cutaneous allergic reactions associated with drug use.

**Materials and methods:** The data were obtained from a Tunisian pharmacovigilance database of adverse drug reactions (ADRs). Analyzed reports were retrieved from the pharmacovigilance unit of Monastir (Tunisia) database collected from 2004 to 2015. The association between drugs and skin reactions was assessed using the case/non-case method, calculating the adverse reaction reporting odds ratio (ROR) and their 95 % confidence intervals as a measure of disproportionality. The “cases” were defined as reports of type III and IV skin allergic reactions (according to gelle and Coombs classifications). The “non-cases” were all other reports.

**Results:** Overall 1800 reports of adverse reactions related to drug use were analyzed; of which 1523 (84 %) were judged as type III and IV skin allergic reactions (cases) and the remaining were considered non-cases. Drug classes associated with a significant increase of ROR were anticonvulsive agents [ROR = 2.2, 95 % CI (1.4–3.3), p < 10^−3^] and antibacterial drugs [ROR = 1.6, 95 % CI (1.3–2), p < 10^−3^]. Among antibacterial agents, betalactams were associated with a significant ROR [1.5; 95 % CI (1.2–1.8), p < 10^−3^]. Regarding betalactams, only oxacilline and the third generation cephalosporins were associated with a significant risk [ROR = 1.9; 95 % CI (1.1–3.2), p = 0.01] and [ROR = 1.81; 95 % CI (1.3–2.4), p < 10^−3^], respectively, while only carbamazepine [ROR = 3; 95 % CI (1.6–5.7), p < 10^−3^] and phenobarbital [ROR = 2.3; 95 % CI (1.1–5.2), p = 0.03] have shown a significant ROR values among anticonvulsive agents.

**Conclusions:** Results highlight the frequency of association of delayed hypersensitivity skin reactions with betalactams, carbamazepine and phenobarbital. Given the widespread use of these drugs, awareness should be raised among patients and prescribers about these risks.

**Keywords:** Case/noncase; Drug allergy; Delayed reactions; Epidemiology

### P130 Delayed hypersensitivity reactions to cephalosporins: a review of seven cases

#### Joana Cosme^1^, Anabela Lopes^1^, Amélia Spínola-Santos^1^, Manuel Pereira-Barbosa^2^

##### ^1^Immunoallergy Department - Hospital de Santa Maria, Centro Hospitalar Lisboa Norte, Lisbon, Portugal, Lisboa, Portugal; ^2^Immunoallergy Department - Hospital de Santa Maria, Centro Hospitalar Lisboa Norte, Lisbon, Portugal; Faculdade de Medicina de Lisboa, Lisboa, Portugal

**Correspondence:** Joana Cosme

*Clinical and Translational Allergy* 2016, **6(Suppl 3)**:P130

**Background:** Delayed hypersensitivity reactions to cephalosporins are poorly described reactions that occur more than 1 h after drug administration. The mechanisms involved seem to be heterogeneous and not totally characterized.

**Objectives:** To characterize the delayed hypersensitivity to cephalosporins and determine the clinical profile of these patients and the cross reactivity with other antibiotics.

**Materials and methods:** Retrospective review from the patients with delayed confirmed hypersensitivity reactions to cephalosporins between 2013 and 2014. The patients were submitted to skin tests with penicilloylpolylysine (PPL), minor determinant mixture (MDM), benzylpenicillin, ampicillin, amoxicillin, cefuroxime, ceftriaxone, cefipime and other antibiotics that were eventually involved. Results for specific IgE to β-lactams and oral provocation challenge tests (OPT) with the alternative and/or culprit β-lactms were also reviewed.

**Results:** Within the 7 patients that were reviewed 71 % were female, with a median age of 56 (32.5–66) years and 43 % had personal history of atopy. The clinical manifestations reported by these patients were delayed urticaria in 57 % of the cases and 43 % described delayed rash. In all cases the symptoms appeared more than 24 h after the beginning of the antibiotic. In 6 (85.7 %) the episodes occurred after cephalosporins intake. One patient had an adverse event with amoxicillin and the sensitization to cephalosporins was only found after investigation. All had negative specific IgE for β-lactams. Four (57.1 %) had sensitization only to cephalosporins. The cephalosporins involved and the delayed results of the IDT and OPT are summarized on Table 1. All the suspected cephalosporins were confirmed: 6 (85.7 %) on the basis delayed IDT, in 1 (14.3 %) cephalosporins’ sensitization was determined after OPT. Regarding cross reactivity with other β-lactams 3 (42.9 %) patients had reactivity to aminopenicillins.

**Fig. 3:**
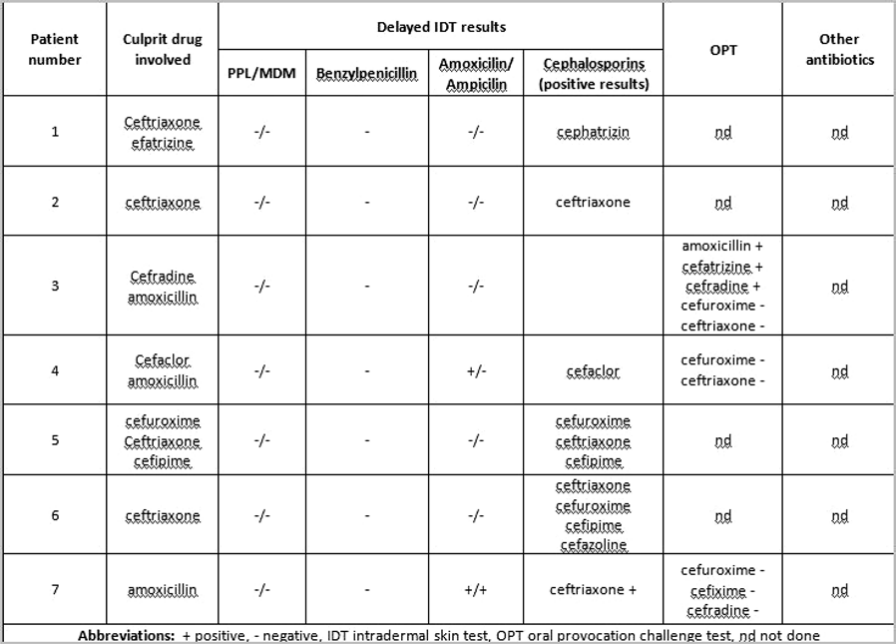
Delayed reactions to cephalosporins: patients’ skin tests and oral provocation tests results

**Conclusions:** Delayed reactions to cephalosporins are associated with delayed clinical manifestations and all had only skin involvement. The reactivity with other β-lactams, within the patients with delayed cephalosporins hypersensitivity, was heterogeneous (Fig. 3).

**Keywords:** β-Lactams; Cephalosporins; Cross-reactivity; Delayed hypersensitivity reactions

### P131 Diclofenac induced allergic contact dermatitis: case series of four patients

#### Sandra Jerkovic Gulin^1^, Anca Chiriac^2^

##### ^1^Department of Dermatology and Venereology, General Hospital Sibenik, Sibenik, Croatia; ^2^Dermatology Department, Nicolina Medical Centre, Apollonia University, „P.Poni“ Research Institute of Macromolecular Chemistry, Iasi, Romania

**Correspondence:** Sandra Jerkovic Gulin

*Clinical and Translational Allergy* 2016, **6(Suppl 3)**:P131

**Background:** Allergic contact dermatitis is an immune-mediated antigen-specific skin reaction to an allergenic chemical that corresponds to a delayed-type hypersensitivity response (type IV reaction). Allergic contact dermatitis should be suspected when skin lesions are localized to the site of previous applications of the culprit drug. The gold standard for diagnosis is patch testing; identification and removal of any potential causal agents is crucial.

Diclofenac cream/gel contains propylene glycol, diclofenac, dimethyl sulfoxide, ethanol and glycerin. It is a widely used non-steroidal anti-inflammatory drug, known to cause especially photoalergic contact reactions.

**Report:** We present four cases of diclofenac induced allergic contact dermatitis, diagnosed based on clinical grounds: intensly itchy eczematous lesions on the sites of drug application, after several days of treatment. No allergic history, no other drug intake were reported by the patients. The application of topical diclofenac was strictly avoided in all cases, potent topical steroids proved to be effective in all cases within two weeeks of therapy.

Patch tests were performed in all cases with European standard batery and with patients’ own cream or gel 3 weeks after completion of local steroid therapy. Reading was performed at 96 h and proved to be positive only to diclofenac. No sun exposure was allowed during the testing, any other treatments were forbidden.

**How this report contributes to current knowledge:** Patients and physicians must be aware of the risk of cutaneous sensitization induced by topical diclofenac, a drug extensively used also as self medication.

**Consent:** Written informed consent was obtained from the patient for publication of this abstract and any accompanying images.

### P132 Late-onset maculopapular rash to irbesartan

#### Bárbara Kong Cardoso, Elza Tomaz, Regina Viseu, Filipe Inácio

##### Hospital de S.Bernardo, Centro Hospitalar de Setúbal, Setúbal, Portugal

**Correspondence:** Bárbara Kong Cardoso

*Clinical and Translational Allergy* 2016, **6(Suppl 3)**:P132

**Background:** Hypertension is one of the most common worldwide diseases. Angiotensin II receptor blocker are one of antihypertensive drugs most prescribed. Some well-known adverse effects of irbesartan are dizziness, urticaria and angioedema. Irbesartan cutaneous non-immediate reactions have been reported previously in a limited number of case reports.

**Report:** Sixty years old female patient referred to our clinic with a 3 week history of an itchy erythematous maculopapular eruption affecting the torso (thorax and abdomen) and proximal part of upper and lower limbs which resolved with hyperpigmentation. The patient reported since then similar but short-lasting lesions that she related to atorvastatin intake.

The patient current medications were: atorvastatin, irbesartan, chlordiazepoxide, levothyroxine, estradiol patch, olanzapine and paroxetine. All of them but irbesartan and paroxetine had been taken for several years. Irbesartan was the latest drug introduced, approximately 2 months before the exanthematous rash beginning and paroxetine was only introduced after the symptoms appearance. There was no history of any infectious disease. Previously performed histopathologic examination of the lesions showed lymphocytic infiltrate and eosinophils in the dermis, compatible to drug reaction.

Irbesartan was changed to diltiazem in patient therapy. Patch tests (PT) to irbesartan, candesartan and atorvastatin (5 % in petrolatum) were performed and a lymphocyte transformation test (LTT) to irbesartan was executed.

**Results:** PT to irbesartan was positive at 48 and 96 h. LTT (irbesartan 100 µg/ml) showed 6.3 stimulation index.

The patient did not refer new lesions after stopping irbesartan and was diagnosed as non-immediate drug reaction due to irbesartan based in clinical, histopathologic and analytic features.

Three months later candesartan was introduced in patient therapy, without skin reaction after 3 months.

**How this report contributes to current knowledge:** There are few reports about non immediate cutaneous side effects due to irbesartan. PT and LTT proved useful in the diagnosis. In spite of the similarity of chemical structure, candesartan may be tried in patients allergic to irbesartan.

**Consent:** Written informed consent was obtained from the patient for publication of this abstract and any accompanying images.

### P133 Nonimmediate hypersensitivity reactions to betalactams: a retrospective analysis

#### Ana Moreira, Susana Cadinha, Ana Castro Neves, Patricia Barreira, Daniela Malheiro, J. P. Moreira Da Silva

##### Centro Hospitalar Vila Nova Gaia e Espinho, Vila Nova Gaia, Portugal

**Correspondence:** Ana Moreira

*Clinical and Translational Allergy* 2016, **6(Suppl 3)**:P133

**Background:** Betalactams (BL) are the most widely prescribed antibiotics and the most frequent cause of drug allergic reactions. Benzylpenicillin, the major responsible for BL drug allergic reactions, has been progressively replaced by amoxicillin and to a lesser extent by cephalosporins or other BL. Our aim was to characterize a series of subjects with suspected nonimmediate hypersensitivity (NIH) to natural penicillins and aminopenicillins/clavulanic acid (CA) and evaluate the usefulness of different diagnostic procedures in the study of these reactions.

**Materials and methods:** A total of 391 patients with suspected betalactam hypersensitivity (BH) were referred to our department from 2009 to 2015 and those with suspected NIH to natural penicillins and aminopenicillins/CA were evaluated. Demographic data, atopy, allergic diseases, comorbidities, clinical manifestations and diagnostic procedures were assessed. BH was confirmed by positive skin tests (ST), drug provocation test (DPT) or long term-challenge (LTC) and considered probable based on a suggestive history and/or positive lymphocytic transformation test (LTT). ST were performed with PPL, MDM, Amoxicillin, Amoxicilin/CA, Ampicillin and Penicillin G; DPT was performed with Amoxicillin, Amoxicilin/CA and Penicillin.

**Results:** 169 (43 %) patients had suspected NIH to natural penicillins and aminopenicillins/CA: 67 % female; median age 37 years (1–81); 34 % atopic; 16 % had rhinitis/rhinoconjunctivitis, 14 % asthma/rhinitis and 8 % chronic urticaria/angioedema. Cutaneous reactions were reported by 84 % of patients. Skin prick tests were negative in all patients tested (129). Intradermal tests were performed in 110 patients and 11 had positive late-reading. Patch tests were positive in 7 out of 111 patients. LTT was positive in 5 out of 11 patients. DPT performed in 120 patients was positive in 10. LTC performed in 86 patients was positive in 8. BH was confirmed in 29 patients (11 by ST, 10 by DPT and 8 by LTC), considered probable in 7 patients, excluded in 103 and inconclusive in 30.

**Conclusions:** As previously reported in the literature, our study suggests that BH usually presents with cutaneous symptoms and affects mainly adults. BH was established in 17 % of the patients, 38 % by ST, 34 % by DPT and 28 % by LTC. According to the ENDA guidelines, DTP is the gold-standard for definitive diagnosis of BH. Nevertheless, 8 patients would not have been diagnosed if LTC was not included in the diagnostic work-up.

**Keywords:** Betalactams; Hypersensitivity; Nonimmediate reactions

### P134 Occupational airborne contact dermatitis to omeprazole

#### Ružica Jurakic-Toncic^1^, Suzana Ljubojevic^1^, Petra Turcic^2^

##### ^1^Department of Dermatology and Venereology, University Hospital Center Zagreb, School of Medicine University of Zagreb, Zagreb, Croatia; ^2^Department of Pharmacology, Faculty of Pharmacy and Biochemistry, University of Zagreb, Zagreb, Croatia

**Correspondence:** Ružica Jurakic-Toncic

*Clinical and Translational Allergy* 2016, **6(Suppl 3)**:P134

**Background:** Omeprazole is a proton pump inhibitor for the treatment of gastric acid-related disorders. It is administered orally.

**Materials and methods:** We present 52-years-old chemist who worked in a pharmaceutical company for 5 years and she was exposed to omeprazole during the manufacturing process. When working in the laboratory, she was wearing protective latex free gloves. Whenever they were manufacturing omeprazole she had eczema with scaling on the eyelids face and neck, later hands were also affected. When she was on sick leave or when she was on vacation she had completely regression of skin dermatitis. The patient was treated with topical corticosteroids, which resulted with temporally regression of skin symptoms.

**Results:** We preformed lymphocyte transformation test (LTT) which was negative. Patch test to baseline series was negative, but patch test to omeprazole (0.1 and 0.5 % in saline solution) was positive at day 2 (+) and day 3 (++).

**Conclusions:** Omeprazole constitute a high-sensitizing chemical. Although direct contact with the skin is not always present, distribution of dust containing omeprazole through the air and deposition on exposed areas may result in an airborne pattern of contact dermatitis. Our case confirms the risk of sensitization to omeprazole from occupational exposure.

**Keywords:** Omeprasole; Proton pump inhibitor; Eczema

**Consent:** Written informed consent was obtained from the patient for publication of this abstract and any accompanying images.

### P135 Ornidazole-induced fixed drug eruption confirmed by positive patch test on a residual pigmented lesion

#### Liesbeth Gilissen, Sara Huygens, An Goossens

##### University Hospitals Leuven, Leuven, Belgium

**Correspondence:** Liesbeth Gilissen

*Clinical and Translational Allergy* 2016, **6(Suppl 3)**:P135

**Background:** Ornidazole is a 5-nitroimidazole derivative commonly prescribed for the treatment of diarrhea caused by anaerobic bacteria. Previous cases of fixed drug eruptions (FDE) from ornidazole were reported in the literature (1–6), however, in only one of them the diagnosis has been confirmed by a positive patch test (5); their patient had also shown a FDE following oral intake of fluconazole (5). In another case (6) cross-reactivity occurred following oral intake with secnidazole.

A 40-year-old male patient presented with two pigmented lesions, one on the upper arm, and one with a central erosion on the glans penis. These had occurred since the intake of ornidazole (Tiberal^®^, Laboratoires SERB, Paris, France) and budesonide (Entocort^®^, AstraZeneca, Brussels, Belgium), 2 weeks previously. The provisional diagnosis of FDE was put forward.

**Materials and methods:** Five weeks later, patch tests with ornidazole (tablet crushed and diluted 30 % in petrolatum) and budesonide (0.01 % in petrolatum) were performed on a residual pigmented lesion on the upper arm, and also on a non-lesional skin site as a control, using vander Bend Chambers^®^ (vander Bend, Brielle, The Netherlands).

**Results:** Only the patch test with ornidazole on the residual pigmented lesion showed a positive reaction at day 4, while the control on the non-lesional skin remained negative. Discontinuation of ornidazole resulted in clearance of the lesions.

**Conclusions:** In the unlikely event of a fixed drug eruption, clear identification of the culprit may be difficult, in particular when multiple medications are administered. Patch testing in a previously affected lesion may identify the causative agent, as in the present case.

**Keywords:** Fixed drug eruption; Ornidazole; Patch testing

**Fig. 4:**
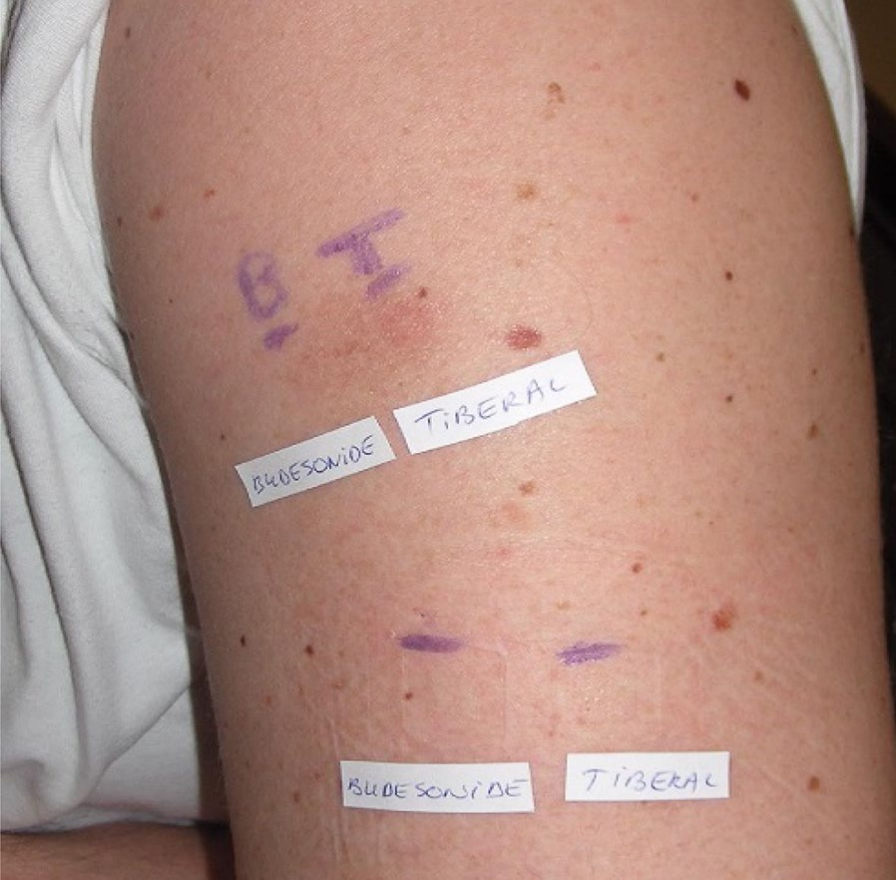
Positive patch test to ornidazole on previously affected skin site

**Consent:** Written informed consent was obtained from the patient for publication of this abstract and any accompanying images (Fig. 4).

**References**

1. Gupta R. Fixed drug eruption due to ornidazole. Indian J Dermatol. 2014;59(6):635.
2. Marya C, et al. Mucosal fixed drug eruption in a patient treated with ornidazole. J Dermatol. 2012;6(1):21–24.
3. Gupta S, et al. Fixed drug eruption caused by ornidazole. Contact Dermat. 2005;53:300–301.
4. Gupta S, et al. Multiple fixed drug eruption caused by ornidazole. Dermatitis 2010;21:330–333.
5. Bavbek S, et al. Fixed drug eruption caused by ornidazole and fluconazole but not isoconazole, itraconazole, ketoconazole and metronidazole. J Dermatol. 2013;40(2):134–135.
6. Sanmukhani J, et al. Fixed drug eruption with ornidazole having cross-sensitivity to secnidazole but not to other nitro-imidazole compounds: a case report. Br J Clin Pharmacol. 2010,69:703–704.

### P136 Repeated delayed reaction induced by amoxicillin and amoxicillin clavulanate

#### Inmaculada Andreu^1^, Ramon Lopez-Salgueiro^1^, Alicia Martinez Romero^2^, Pau Gomez Cabezas^2^

##### ^1^IIS La Fe, Valencia, Spain; ^2^CIPF, Valencia, Spain

**Correspondence:** Inmaculada Andreu

*Clinical and Translational Allergy* 2016, **6(Suppl 3)**:P136

**Background:** There are no techniques validated for the diagnosis of drug induced delayed allergic reactions. In vivo tests are inconvenient and in vitro tests are neither sensitive nor validated.

We describe a case of recurrent delayed reactions related to therapy with amoxicillin and amoxicillin clavunalate by in vivo and in vitro tests.

**Materials and methods:** The patient developed repeated episodes of generalized skin rash, pruritus, malaise, chest pain and dysphagia after 5–7 days of therapy with amoxicillin clavunalate or amoxicillin. Blood tests showed an elevation in CRP, GGT, fibrinogen and monocytosis during the acute phase. The reactions resolved with corticosteroids and antihistamines.

Intradermal and patch tests were performed with amoxicillin and amoxicillin-clavunalate. Total IgE and specific IgE and IgG to amoxicillin were measured by ImmunoCAP^®^. Cell activation analysis by the culprit drugs and clavulanate was performed by flow cytometry. Basophil Activation Test (BASOTEST^®^) was considered positive if activation ≥5 % or SI (stimulation index) ≥2. Mononuclear cell activation was evaluated by the expression of CD69 after drug exposure for 48 h. Antibodies anti-CMV, Herpes, Epstein-Barr and parvovirus were analyzed.

**Results:** Intradermal and patch tests with amoxicillin and amoxicillin clavunate acid were negative. Total IgE: 198 kUI/l. Specific IgE for amoxicillin and penicillin were negative (0.07 kUI/l). Specific IgG was 3.42 mgA/l. Basophil activation test was positive for amoxicillin (1/160: 10.87 %; SI 16.73–1/40: 48.55 %; SI 35.69) and amoxicillin clavunalate (1/160: 22.76 %; SI 16.73–1/40: 74.68 %; SI 54.91), but negative for clavunalate (100 μg/ml: 3.55 %; SI 1.26–200 μg/ml: 3.98 %; SI 1.41). A threefold increase occurred when the mononuclear cells were treated with amoxicillin, 2.5-fold increase with clavulanate and tenfold increase when the samples were treated with amoxicilline clavunalate. Serologies showed positive IgG for Epstein-Barr, herpes and parvovirus.

**Conclusions:** Delayed adverse reactions to amoxicillin and amoxicillin clavulanate may be diagnosed by alternative tests such as basophil and lymphocyte activation tests when conventional in vivo and vitro tests show negative results. We hypothesize that specific IgG may induce anaphylactic reactions through basophil activation. Although the role of T cell activation is not clear, we observed a synergic effect of both drugs.

**Consent:** Written informed consent was obtained from the patient for publication of this abstract and any accompanying images.

### P137 Systemic photosensitivity from fenofibrate in a patient photo-sensitized to ketoprofen

#### Liesbeth Gilissen, An Goossens

##### University Hospitals Leuven, Leuven, Belgium

**Correspondence:** Liesbeth Gilissen

*Clinical and Translational Allergy* 2016, **6(Suppl 3)**:P137

**Background:** Fenofibrate is widely used in the treatment of hypercholesterolemia and hypertriglyceridemia. Cutaneous side effects such as pruritus, rash, urticarial lesions, but also cases of photosensitivity have been previously described (1–3).

A 47-year old female patient presented with a severe vesicular eruption involving the sun-exposed body areas, except under the wristwatch. The face was only slightly affected to which she had applied a sunscreen. She had been taken several medications, i.e., fenofibrate (Lipanthyl^®^, Mylan EPD, Wavre, Belgium), metformine (Metformax^®^, Menarini, Zaventem, Belgium), spironolactone, and allopurinol (Zyloric^®^, Laboratoires SMB, Brussels, Belgium).

**Materials and methods:** Patch tests with the European baseline series and photo-patch tests with the European photo-patch test series were performed, using IQ Ultra^®^ Chambers (Chemotechnique Diagnostics, Vellinge, Sweden).

**Results:** A strong positive photo-patch test to ketoprofen (D2 −, D4 +++, D8 +++) and also a positive patch test to benzophenone-3 (D2 +, D4 ++) were obtained.

**Conclusions:** Photo-allergic contact dermatitis from ketoprofen often gives rise to simultaneous reactions to other nonsteroidal anti-inflammatory drugs (NSAIDs), sunscreens, and fragrance components (4), as well as to fenofibrate (5), which, administered systemically, may induce photosensitivity. The benzophenone moiety is responsible for the photosensitization reaction (3.5).

**Keywords:** Fenofibrate; Patch testing; photo-allergic contact dermatitis; Systemic contact dermatitis

**Fig. 5:**
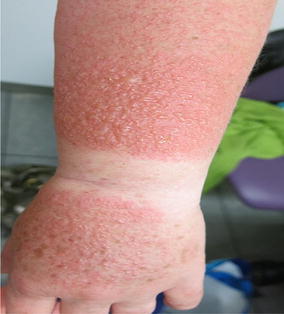
Systemic photosensitivity from fenofibrate (arm, wrist) following ketoprofen photo-sensitization

**Consent:** Written informed consent was obtained from the patient for publication of this abstract and any accompanying images (Fig. 5).

**References**

1. Gardeazabal J, Gonzalez M, Izu R, Gil N, Aguirre A, Diaz-Perez J. Phenofibrate-induced lichenoid photodermatitis. Photodermatol Photoimmunol Photomed. 1993;9(4):156–158.
2. Barbaud A, Schmutz J, Trechot P, et al. Photoallergie au fenofibrate (Lipanthyl^®^): à propos de 3 cas. Lett GERDA 1992;9:6–10.
3. Serrano G, Fortea J, Latasa J, Millan F, Janes C, Bosca F, Migual A. Photosensitivity induced by fibric acid derivatives and its relation to photocontact dermatitis to ketoprofen. J Am Acad Dermatol. 1992;27(2):204–208.
4. Matthieu L, Meuleman L, Van Hecke E, Blondeel A, Dezfoulian B, Constandt L, Goossens A. Contact and photocontact allergy to ketoprofen. The Belgian experience. Contact Dermat. 2004;50(4):238–241.
5. Leroy D, Dompmartin A, Szczurko C, Michel M, Louvet S. Photodermatitis from ketoprofen with cross-reactivity to fenofibrate and benzophenones. Photodermatol Photoimmunol Photomed. 1997;13(3):93–97.

## Poster Walk 16: HLA genetics (P138–P146)

### P138 A copy number variation in ALOX5 and PTGER1 is associated with nonsteroidal anti-inflammatory drugs induced urticaria and/or angioedema

#### Pedro Ayuso Parejo^1^, Maria Del Carmen Plaza-Serón^1^, Inmaculada Doña^2^, Natalia Blanca López^1^, Carlos Flores^3^, Luisa Galindo^2^, Ana Molina^4^, James Richard Perkins^4^, Jose Antonio Cornejo-García^4^, José Augusto García-Agúndez^5^, Elena García-Martín^6^, Paloma Campo^2^, María Gabriela Canto^1^, Miguel Blanca^2^

##### ^1^Allergy service, Infanta Leonor Hospital, Madrid, Spain, Madrid, Spain; ^2^Allergy Unit, IBIMA, Regional University Hospital of Malaga, UMA, Malaga, Spain, Málaga, Spain; ^3^Research Unit and Centro de Investigación Biomédica en Red de Enfermedades Respiratorias de Enfermedades Respiratorias, Instituto de Salud Carlos III, Madrid, Spain, Madrid, Spain; ^4^Research Laboratory, IBIMA, Malaga Regional University Hospital, UMA, Malaga, Spain, Málaga, Spain; ^5^Department of Pharmacology, University of Extremadura, Cáceres, Spain, Cáceres, Spain; ^6^Department of Biochemistry and Molecular Biology, University of Extremadura, Cáceres, Spain., Caceres, Spain

**Correspondence:** James Richard Perkins

*Clinical and Translational Allergy* 2016, **6(Suppl 3)**:P138

**Background:** Cross-intolerance to nonsteroidal anti-inflammatory drugs (NSAIDs) is a class of hypersensitivity reaction in which NSAIDs-induced urticaria and/or angioedema (NIUA) is the most frequent entity. It is thought to involve dysregulation of the arachidonic acid (AA) pathway. However, this mechanism has not been confirmed for NIUA. In this work we assessed copy number variations (CNVs) in 8 of the main genes involved in the AA pathway and their possible genetic association with NIUA.

**Materials and methods:** CNVs in *ALOX5, LTC4S, PTGS1, PTGS2,PTGER1,PTGER2,PTGER3* and *PTGER4* were analyzed using TaqMan copy number assays. Genotyping was carried out by real time quantitative PCR. Individual genotypes were assigned using the CopyCaller™ Software. Statistical analysis was carried out using GraphPad prism 5, PLINK, EPIDAT and R version 3.1.2.

**Results:** 151 cases and 139 controls were analyzed during the discovery phase and 148 cases and 140 controls were used for replication. CNVs in open reading frames were found for *ALOX5, PTGER1, PTGER3* and *PTGER4.* Statistically significant changes in CNVs between NIUA and controls were found for *ALOX5* (*p*_c_ = 0.017) and *PTGER1* (*p*_c_ = 1.22E−04). Moreover, we described that there was not a correlation between the presence of a deletion in *ALOX5* with the presence of a deletion in *PTGER1* (R^2^ = 0.274).

**Conclusions:** This study represents the first analysis showing an association between CNVs in exonic regions of *ALOX5* and *PTGER1* and NIUA. This suggests an important role of CNVs in this pathology that should be further explored in future studies.

**Keywords:** NSAIDs hypersensitivity; Genetic association study; Cyclooxygenases; Prostaglandin E receptors; Arachidonate 5-lypoxygenase

### P139 Association of galectin-3 (LGALS3) single nucleotide polymorphisms with non-steroidal anti-inflammatory drugs-induced urticaria/angioedema

#### José Antonio Cornejo-Garcia^1^, Inmaculada Doña^2^, Rosa María Guéant-Rodríguez^3^, Natalia Blanca-López^4^, María Carmen Plaza-Serón^5^, Raquel Jurado-Escobar^5^, Esther Barrionuevo^2^, María Salas^2^, María Luisa Galindo^2^, Gabriela Canto^4^, Miguel Blanca^2^, Jean-Louis Guéant^3^

##### ^1^Research Laboratory and Allergy Unit, IBIMA, Regional University Hospital of Malaga, UMA, Malaga, Spain; ^2^Allergy Unit, IBIMA, Regional University Hospital of Malaga, UMA, Malaga, Spain; ^3^University of Lorraine and University Hospital Center (CHU) of Nancy, INSERM U-954, Faculty of Medicine, Vandoeuvre-Les-Nancy, France; ^4^Allergy Service, Infanta Leonor University Hospital, Madrid, Spain; ^5^Research Laboratory, IBIMA, Regional University Hospital of Malaga, UMA, Malaga, Spain

**Correspondence:** José Antonio Cornejo-Garcia

*Clinical and Translational Allergy* 2016, **6(Suppl 3)**:P139

**Background:** NSAIDs are the first cause of drug hypersensitivity reactions (DHRs), being those mediated by non-specific immunological mechanisms (cross-intolerance, CI) the most frequent. Skin is the most commonly affected organ and NSAIDs-induced urticaria/angioedema (NIUA) the most frequent clinical entity. Galectin-3 plays an important role in the biological responses of skin cells, and it is being regarded as a novel therapeutic target for a variety of skin disorders. We analyzed the association of several nonsynonymous single nucleotide polymorphisms (SNPs) in *LGALS3* with CI in a large group of patients with different clinical entities, including NIUA, NSAIDs-exacerbated respiratory disease and a mixed pattern of response that includes both skin and respiratory involvement.

**Materials and methods:** The population studied was obtained from two Allergy Services integrated into the Spanish network RIRAAF. Cases included had to develop more than two episodes of CI after the intake of two or more NSAIDs from different chemical groups. We studied several SNPs in *LGALS3* using TaqMan^®^ probes. s both skin and respiratory involvement.

**Results:** A total of 504 subjects with CI to NSAIDs were included and 271 age and sex-matched healthy controls. Statistically significant associations were found between NIUA and *LGALS3* rs11125 (p < 0.001) and rs4644 (p = 0.032).

**Conclusions:** Our results suggest a role for SNPs in *LGALS3* in NIUA, the most important clinical entity induced by HRDs. The association of such genetic variants with NIUA provides us with new clues for understanding their underlying mechanisms. Further studies are required to analyze the potential role of other genetic variants in galectins and related genes in HRDs to NSAIDs.

### P140 Detection of T cell responses to ticlopidine using peripheral blood mononuclear cells from HLA-A*33:03+ healthy donors

#### Toru Usui, Arun Tailor, Lee Faulkner, John Farrell, Ana Alfirevic, B. Kevin Park, Dean J. Naisbitt

##### University of Liverpool, Liverpool, United Kingdom

**Correspondence:** Toru Usui

*Clinical and Translational Allergy* 2016, **6(Suppl 3)**:P140

**Background:** Ticlopidine is an anti-platelet drug used in the treatment of atherothrombosis and is known to cause idiosyncratic drug induced liver injury (DILI). The recent identification of human leukocyte antigen (HLA)-A*33:03 as a susceptibility factor and the delayed nature of the liver injury are both indicative of an immune pathogenesis. However, the role of the adaptive immune system in ticlopidine-induced DILI has not yet been defined.

The aim of this study was to investigate whether drug-specific T cell responses could be detected in healthy volunteers who expressed HLA-A*33:03. Any T cell responses would then be fully characterized for drug specificity, HLA restriction and the mechanism(s) of antigen presentation

**Materials and methods:** Peripheral blood mononuclear cells were isolated from HLA-typed healthy volunteers who did or did not express the risk allele: HLA-A*33:03. Subsequently, naïve T-cells were separated and co-cultured with autologous monocyte-derived dendritic cells (DCs) in the presence of the ticlopidine for a period of 8 days, to expand the number of drug-responsive T-cells. T-cells were then harvested and incubated with freshly prepared dendritic cells and drug to test their antigen specificity using readouts for cell proliferation and cytokine secretion. T-cell clones were also generated following priming and drug-specific T cell clones analysed for cytokine secretion and HLA restriction.

**Results:** Using the DC priming assay all four HLA-A*33:03 positive donors showed ticlopidine-specific proliferation and IFNγ secretion. However, no ticlopidine-specific responses were detected in HLA-A*33:03 negative donors. Around one thousand CD8 positive clones were generated from three HLA-A*33:03 positive donors, but ticlopidine specific clones were only obtained from one donor. These CD8 positive clones showed ticlopidine-specific IFNγ secretion. This response was restricted by HLA-A*33:03 and was not dependent on the presence of antigen presenting cells.

**Conclusions:** In conclusion, ticlopidine-specific T-cells were detected in healthy volunteers expressing the risk allele HLA-A*33:03 using the DC priming assay.

### P141 Epistasis approaches to identify novel genes potentially involved in NSAIDs hypersensitivity

#### James Richard Perkins^1^, Jose Antonio Cornejo García^1^, Oswaldo Trelles^2^, Inmaculada Doña^3^, Esther Barrionuevo^3^, María Salas^3^, María Auxiliadora Guerrero^3^, Miguel Blanca^3^, Alex Upton^2^

##### ^1^Research Laboratory, IBIMA, Regional University Hospital of Malaga, UMA, Malaga, Spain; ^2^Department of Computer Architecture, University of Malaga, Malaga, Spain; ^3^Allergy Unit, IBIMA, Regional University Hospital of Malaga, UMA, Malaga, Spain

**Correspondence:** James Richard Perkins

*Clinical and Translational Allergy* 2016, **6(Suppl 3)**:P141

**Background:** A handful of genome wide association studies (GWASs) have been performed to detect genetic variation associated with NSAID hypersensitivity, by comparing the frequencies of hundreds of thousands of single nucleotide polymorphisms (SNPs) between NSAIDs hypersensitive subjects and control individuals. This is done on a SNP-by-SNP basis, meaning that each variant is considered independently. However, the effect of a single SNP on phenotype is influenced by other factors, including the individual’s genetic background. An alternative approach to analyse this data is the use of epistasis methods, which look for associations between pairs of SNPs and a phenotype that are stronger than the sum of the individual SNP-phenotype associations, suggesting an interaction between the SNPs.

**Materials and methods:** We analysed data from an NSAIDs hypersensitivity GWAS using an epistasis detection method, MBMDR. The resulting pairs of interacting SNPs were used to build networks, both at the SNP level and at the gene level, by mapping SNPs to their closest protein coding gene (within 500 kb). These networks were analysed to identify SNPs and genes with a potential role in NSAIDs hypersensitivity using graph metrics. This approach identifies important hub nodes based on the strength and number of connections they have, which can be interpreted as the number of distinct pair-wise interactions they are involved in.

**Results:** Our approach identified a number of genes with potential roles in NSAIDs hypersensitivity, including genes potentially involved in asthma (*KCNB2*, *PPP1R3C and CSMD1*), rhinitis (*SCN11A*) and immune system functioning (*TSPAN33*). It also found a number of genes with a potential role in lipid related processes, such as *SGSM2* and *BUB3*. The gene *CGNL1, whose expression has been shown to change following* aspirin intake, was also identified. Additionally, pathway analysis of the networks found enrichment for ALK1 and TGF-beta signalling.

**Conclusions:** The study presented here shows how weighted epistatic analysis approaches can complement traditional SNP-by-SNP analyses. Additional studies are necessary to replicate these findings, and they should be applied to other NSAIDs hypersensitivity pathologies. Moreover, the mechanisms by which the SNPs and genes identified here affect NSAIDs hypersensitivity must be investigated further.

**Keywords:** NSAIDs-hypersensitivity; GWAS; Genetics; Epistasis; Systems biology

### P142 Genetic predisposition of cold medicine related SJS/TEN with severe ocular complications

#### Mayumi Ueta^1^, Hiromi Sawai^2^, Chie Sotozono^3^, Katushi Tokunaga^2^, Shigeru Kinoshita^1^

##### ^1^Department of Frontier Medical Science and Technology for Ophthalmology, Kyoto Prefectural University of Medicine, Kyoto, Japan; ^2^Department of Human Genetics, Graduate School of Medicine, The University of Tokyo, Tokyo, Japan; ^3^Department of Ophthalmology, Kyoto Prefectural University of Medicine, Kyoto, Japan

**Correspondence:** Mayumi Ueta

*Clinical and Translational Allergy* 2016, **6(Suppl 3)**:P142

**Background:** Stevens–Johnson syndrome (SJS) is an acute inflammatory vesiculobullous reaction of the skin and mucosa such as the ocular surface, oral cavity, and genitals. In patients with extensive skin detachment and a poor prognosis, the condition is called toxic epidermal necrolysis (TEN). Severe ocular complications (SOC) appear in about 40 % of SJS/TEN patients diagnosed by dermatologists. Among SJS- and TEN patients, especially those with SJS/TEN with SOC, cold medicines (CM) including multi-ingredient cold medications and non-steroidal anti-inflammatory drugs were the main causative drugs. We reported that in the Japanese, CM-SJS/TEN with SOC was strongly associated with *HLA*-A*02:06 and significantly associated with *HLA*-B*44:03; in Indian and Brazilian caucasian populations it was associated with *HLA*-B*44:03, and in Koreans with *HLA*-A*02:06. In our 1st genome-wide association study, we analyzed our SJS/TEN with SOC patients using the Affymetrix GeneChip Mapping 500 K array set, and found an association between prostaglandin E receptor 3 (*PTGER3*) and SJS/TEN with SOC, and subsequently found that this association was stronger in patients with CM-SJS/TEN with SOC than in patients with all SJS/TEN with SOC. In this study, we performed the 2nd genome-wide association study using the Japanese CM-SJS/TEN with SOC.

**Materials and methods:** We performed a genome-wide association study of Japanese 117 CM-SJS/TEN with SOC patients and 691 controls using the Affymetrix AXIOM Genome-Wide ASI 1 array. For the examination of 17 SNPs in regions near *TSHZ2*, we genotyped 101 of the patients and 200 of the 691 controls.

**Results:** Manhattan plots showed that the HLA-A region was most strongly associated with the susceptibility for CM-SJS/TEN with SOC. Outside of the *HLA* region, there were 60 SNPs with a value of p < 10^−3^ in either allele frequency, the dominant-, or the recessive model. Among the 11 SNPs of 8 genes with p < 10^−5^, *IKZF1* manifested particularly low p values [rs897693 (CC + CT vs TT), OR 5.0, p = 2.1 × 10^−8^]. Among the 11 SNPs of 8 genes whose value in our second genome-wide association study was p < 10^−5^, *TSHZ2* also had especially low p values [rs4809905 (AA + AG vs GG), OR 0.3, p = 1.5 × 10^−7^]. Furthermore, we have examined 17 SNPs in regions near *TSHZ2* using 101 CM-SJS/TEN patients and 200 controls, and found that 8 SNPs exhibited a significant genome-wide association with CM-SJS/TEN with SOC.

**Conclusions:***TSHZ2* is one of the susceptibility gene for CM-SJS/TEN with SOC in the Japanese.

**Keywords:** Stevens–Johnson syndrome (SJS); Toxic epidermal necrolysis (TEN); Severe ocular complications (SOC); Cold medicines (CM); Genetic predisposition

### P143 HLA-B*13:01 and dapsone induced hypersensitivity in Thai population

#### Chonlaphat Chonlaphat Sukasem^1^, Patompong Satapornpong^2^, Therdpong Tempark^3^, Pawinee Rerknimitr^4^, Kulprapat Pairayayutakul^5^, Jettanong Klaewsongkram^6^

##### ^1^Ramathibodi hospital, Mahidol university, Bangkok, Thailand; ^2^Division of Pharmacogenomics and Personalized Medicine, Department of Pathology, Faculty of Medicine Ramathibodi Hospital, Mahidol University, Bangkok, Thailand; ^3^Division of Pediatric Dermatology, Department of Pediatrics, Faculty of Medicine, Chulalongkorn University, Bangkok, Thailand; ^4^Division of Dermatology, Department of Medicine, Faculty of Medicine, Chulalongkorn University, Bangkok, Thailand; ^5^6Raj Pracha Samasai Institute, Department of disease control, ministry of public health, Nonthaburi, Thailand; ^6^Division of Allergy and Clinical Immunology, Department of Medicine, Faculty of Medicine, Allergy and Clinical Immunology Research Group, Chulalongkorn University, Bangkok, Thailand

**Correspondence:** Chonlaphat Chonlaphat Sukasem

*Clinical and Translational Allergy* 2016, **6(Suppl 3)**:P143

**Background:** Dapsone is regarded as the treatment of choice for infections, various dermatological diseases and inflammatory diseases. Drug hypersensitivity syndrome is considered as a severe cutaneous adverse drug reaction which is commonly precipitated by dapsone. A previous publication showed that the *HLA*-B*13:01 allele is a strong marker for dapsone-induced hypersensitivity syndrome of leprosy patients in China. Although dapsone-induced hypersensitivity reactions is common, however there are no data describing whether *HLA*-B*13:01 could be used as a genetic marker for prediction of dapsone-induced hypersensitivity reactions in Thai.

**Objective:** The aim of this study was to investigate the predisposition of dapsone-induced DHS, conferred by *HLA*-B*13:01 in a Thai population.

**Materials and methods:** A total of 22 patients, included 11 patients in dapsone-induced hypersensitivity syndrome, 11 patients in dapsone-tolerant control group (dapsone treatment for more than 6 months but without any episode of dapsone-induced hypersensitivity syndrome) and 986 healthy Thai population group. HLA-B genotype were determined by two-stage sequence-specific oligonucleotide probe system (SSOP). This study was approved by the Ethics Committee of Ramathibodi hospital.

**Results:** The results presented *HLA*-B*13:01 allele in patients of dapsone-induced hypersensitivity syndrome, dapsone tolerant controls and healthy Thai population were 63.64 (7/11), 18.18 (2/11) and 13.59 % (134/986) respectively. The *HLA*-B*13:01 allele were not significantly different between DIHS cases and dapsone tolerant controls (p value 0.0805, OR 7.88, 95 % CI 1.11–56.13), but there were significant different between DIHS cases and healthy Thai population (p value 0.0002, OR 11.13, 95 % CI 3.21–38.52).

**Conclusions**

**Discussion:** Although the samples size of DIHS cases and dapsone tolerant controls in this research were limited, there were found the *HLA*-B*13:01 allele could predisposition toward to dapsone-induced hypersensitivity in Thailand.

**Keywords:** HLA-B*13:01; Dapsone; Thai; Dapsone induced hypersensitivity reactions; DISH

### P144 HLA-B*15:02 alleles and lamotrigine-induced cutaneous adverse drug reactions in Thai

#### Chonlaphat Sukasem^1^, N. Koomdee^1^, T. Jantararoungtong^1^, S. Santon^1^, A. Puangpetch^1^, U. Intusoma^2^, W. Tassaneeyakul^3^, V. Theeramoke^4^

##### ^1^Division of Pharmacogenomics and Personalized Medicine, Department of Pathology, Faculty of Medicine Ramathibodi Hospital, Mahidol University, Bangkok, Thailand; ^2^Department of Pediatrics, Faculty of Medicine, Prince of Songkla University, Songkla, Thailand; ^3^Department of Pharmacology, Faculty of Medicine, Khon Kaen University, Khon Kaen, Thailand; ^4^Manarom Hospital, Bangkok, Thailand

**Correspondence:** Chonlaphat Sukasem

*Clinical and Translational Allergy* 2016, **6(Suppl 3)**:P144

**Background:** Lamotrigine (LTG) is one of the antiepileptic drugs (AEDs) commonly used in the treatment of epilepsy for partial seizures and psychiatric patients in clinical practice. Several HLA alleles have been associated with Lamotrigene-induced cutaneous adverse drug reactions (cADRs) in different populations, however, this has not been investigated in Thai.

**Objective:** The aims of this study were to determine the possible associations of lamotrigine-induced cADRs with the *HLA*-*B* alleles in Thai patients.

**Materials and methods:** A total of 645 patients, including 16 patients with lamotrigine-induced cADRs, vary from maculopapular exanthema (MPE) to Stevens–Johnson syndrome/toxic epidermal necrolysis (SJS/TEN), 50 lamotrigine-tolerant controls and 580 healthy controls were included in this study. *HLA*-*B* genotyping was performed. This case–control study was approved by the Ethics Committee of Ramathibodi Hospital.

**Results:** The differences in the starting dosage of LTG among the SJS/TEN, MPE, and LTG-tolerant control groups were not statistically significant. *HLA*-B*15:02 allele was present in 33.3 % (1/3; LTG-induced SJS/TEN group), 38.5 % (5/13; LTG-induced MPE group) of Thai patients but only 12.0 % (6/50) of lamotrigine-tolerant controls and 14.8 % of 580 healthy controls. There was significant difference in the frequency of subjects with the *HLA*-B*1502 allele between SJS/TEN and LTG-tolerant groups (p < 0.05, OR 3.559, 95 % CI 1.71–7.40), and healthy controls (p = 0.003, OR 2.75, 95 % CI 1.38–5.47). Significant difference between the MPE group and LTG-tolerant group (p < 0.05, OR 4.613, 95 % CI 2.23–9.51), and healthy controls (p < 0.05, OR 3.565, 95 % CI 1.80–7.03).

**Conclusions:** In conclusion, in this study, we found a statistically significant association between the HLA-B*1502 allele and LTG-induced cADRs in Thai population. Therefore, an application of HLA-B*1502 as a screening test before prescribing LTG will help to prevent LTG-induced cADRs in Thailand (Tables 2, 3, 4).

**Table 2:** Clinical characteristics of LTG-induced cADRs group and LTG-tolerant group

	LTG-induced cADRs group (n = 16)	LTG-tolerant group (n = 50)	p value
Gender (male: female)	6:10	12:38	0.340
	1:2 (SJS/TEN)		1.000
	5:8 (MPE)		0.353
Mean age (years)	35.5 ± 21.7	38.2 ± 19.0	0.646
	52.3 ± 24.0 (SJS/TEN)		0.415
	32.0 ± 20.9 (MPE)		0.353
Starting dosage of LTG (mg/day)	51.6 ± 26.6	77.6 ± 62.0	0.109
	41.7 ± 14.4 (SJS/TEN)		0.325
	53.8 ± 28.6 (MPE)		0.186

No significant differences were observed in gender, mean age, and mean starting dosage of LTG between cases and LTG-tolerant controls

**Table 3:** Comparison of *HLA*-B*15:02 frequency among groups

Group1 (frequency of *HLA*-B*15:02)	Group2 (frequency of *HLA*-B*15:02)	p value	OR^a^	95 % CI^b^
LTG-induced SJS/TEN	LTG-tolerant group	0.0004	3.559	1.71–7.40
LTG-induced SJS/TEN	Healthy control	0.003	2.750	1.38–5.47
LTG-induced SJS/TEN	LTG-induced MPE	1.000	0.800	0.06–11.29
LTG-induced MPE	LTG-tolerant group	<0.05	4.613	2.23–9.51
LTG-induced MPE	Healthy control	<0.05	3.565	1.81–7.03

^a^OR: odds ratio

^b^CI: confidence interval

**Table 4:** The association of individual HLA-B allele with lamotrigine induced cutaneous adverse drug reactions (cADR)

HLA-B allele	Lamotrigine induced cADR (n = 16)	Lamotrigine tolerant (n = 50)	Healthy control (n = 580)	cADR cases versus lamotrigine tolerant control		cADR cases versus healthy control	
				OR (95 % CI)	p value	OR (95 % CI)	p value
1301	2 (12.5)	6 (12)	81 (14)	1.083 (0.468–2.505)	0.852	0.907 (0.403–2.043)	0.814
1502	6 (37.5)	6 (12)	86 (14.8)	4.423 (2.142–9.134)	0.000026	3.418 (1.730–6.751)	0.000273
1513	1 (6.3)	0 (0)	10 (1.7)	2.064 (1.785–2.386)	0.029	3.128 (0.616–15.886)	0.279
1535	1 (6.3)	1 (2)	12 (2.1)	3.128 (0.616–15.886)	0.279	3.128 (0.616–15.886)	0.279
3901	1 (6.3)	1 (2)	11 (1.9)	3.128 (0.616–15.886)	0.279	3.128 (0.616–15.886)	0.279
4001	3 (18.8)	13 (24)	109 (18.8)	0.743 (0.377–1.464)	0.389	1.000 (0.493–2.027)	1.000
4403	4 (25)	2 (4)	48 (8.3)	8.000 (2.669–23.982)	0.000025	3.833 (1.634–8.991)	0.001
4601	5 (31.3)	11 (22)	101 (17.4)	1.539 (0.844–3.006)	0.149	2.194 (1.120–4.296)	0.020
5101	2 (12.5)	2 (4)	43 (7.4)	3.545 (1.114–11.280)	0.024	1.963 (0.749–5.146)	0.164
5201	1 (6.3)	1 (2)	16 (2.8)	3.128 (0.616–15.886)	0.279	2.064 (0.502–8.493)	0.498
5801	2 (12.5)	5 (10)	116 (20)	1.330 (0.554–3.190)	0.523	0.591 (0.276–1.265)	0.173

*OR* odds ratio, *CI* confidence interval

**Keywords:** Lamotrigine; HLA-B*1502; Cutaneous adver sereactions; Stevens–Johnson syndrome; Toxic epidermal necrolysis

### P145 HLA-B*38:01 and HLA-A*24:02 allele frequencies in Spanish patients with lamotrigine-induced SCARs

#### Teresa Bellón^1^, Elena Ramirez^2^, Alberto Manuel Borobia^2^, Hoi Tong^3^, Jose Luis Castañer^4^, Francisco José De Abajo^5^

##### ^1^IdiPAZ, Madrid, Spain; ^2^Hospital Universitario La Paz, IdiPAZ, Madrid, Spain; ^3^Hospital Universitario La Paz, Madrid, Spain; ^4^Hospital Universitario Ramón y Cajal, Madrid, Spain; ^5^Hospital Universitario Príncipe de Asturias, Alcalá De Henares, Madrid, Spain

**Correspondence:** Teresa Bellón

*Clinical and Translational Allergy* 2016, **6(Suppl 3)**:P145

**Background:** Some HLA-I alleles are risk factors for the development of severe cutaneous adverse drug reactions (SCARs). HLA-B1502 is strongly associated with the development of Stevens Johnson syndrome/toxic epidermal necrolysis (SJS/TEN) to carbamazepine (CBZ) in Southeast Asian populations where the allele is prevalent. On the other hand, HLA-A3101 has been found to be a risk factor for (CBZ)-induced drug reaction with eosinophilia and systemic symptoms (DRESS) in European and Asian patients. HLA-A3101 has been weakly associated to CBZ-induced SJS/TEN in Europeans, and no association has been found with other aromatic antiepileptic drugs (AEDs). No HLA-I alleles have been definitely associated with lamotrigine (LTG)- or phenytoine (PHE)-induced SCARs in Europeans

**Materials and methods:** To explore the association of HLA-I allele frequencies with SCARs to AEDs in our population, 12 patients with DRESS and 14 cases with SJS/TEN included in the Spanish registry PIEL*enREd* were studied. Six cases were related to LTG (3 SJS/TEN; 3 DRESS), 6 were related to CBZ (2 SJS/TEN; 4 DRESS), and 14 cases were induced by PHE (9 SJS/TEN; 5 DRESS). DNA was prepared from total blood and four digits HLA typing was performed. The frequencies of HLA-I alleles in patients with LTG-induced SCARs were compared with frequencies in patients within the other groups, and with previously published HLA-I frequencies in 253 Spanish individuals (Balas et al. Tissue Antigens, 2010).

**Results:** HLA-B3801 allele was present in all three cases of LTG-induced SJS/TEN suggesting a strong association (OR 37.92; 95 % CI 5.4–269.53; p < 0.0001). Only 2 out of 9 SJS/TEN cases induced by PHE were carriers of HLA-B3801 (OR 4.74; 95 % CI 0.67–25.12; p = 0.09). None of the 12 DRESS cases analyzed were carriers of HLA-B3801. On the other hand, the 3 LTG-induced DRESS patients were carriers of HLA-A2402 showing a significant association as compared to the control sample (OR 5.25; 95 % CI 1.0–24.75; p = 0.04). HLA-A2402 was found in 3 out of 5 cases of PHE-induced DRESS (OR 4.5; 95 % CI 0.88–20.27; p = 0.054) and was absent in all CBZ cases and in SJS/TEN cases to PHE.

**Conclusions:** Our results suggest that HLA-B3801 may be a risk factor for LTG-induced SJS/TEN, in agreement with previous data (Lonjou et al. Pharmacogenetics and Genomics 2008). On the other hand HLA-A2402 might be related with LTG-induced DRESS or exantematic reactions, as recently published in the Norwegian population (Shirzadi et al. Epilepsy Research 2016).

### P146 Overrepresentation of a class II HLA haplotype in severe hypersensitivity type I reactions to carboplatin

#### Violeta Régnier Galvao^1^, Rebecca Pavlos^2^, Elizabeth Mckinnon^2^, Kristina Williams^3^, Alicia Beeghly-Fadiel^4^, Alec Redwood^2^, Elizabeth Phillips^3^, Mariana Castells^1^

##### ^1^Brigham and Women`s Hospital, Division of Rheumatology, Immunology and Allergy, Harvard Medical School, Boston, United States; ^2^Institute for Immunology and Infectious diseases, Murdoch University, Perth, Australia; ^3^Vanderbilt University Medical Center, Nashville, United States; ^4^Vanderbilt Epidemiology Center, Institute for Medicine and Public Health; Vanderbilt University Medical Center, Vanderbilt-Ingram Cancer Center, Nashville, United States

**Correspondence:** Mariana Castells

*Clinical and Translational Allergy* 2016, **6(Suppl 3)**:P146

**Background:** HLA class I and class II genotyping has identified patients at risk for both T-cell mediated and IgE/mast cell mediated severe drug hypersensitivity (HSR). Class II HLA-DRA variants have been associated with immediate allergic reactions to beta-lactams and the HLA-DRB1*07:01 allele has been associated with IgE-mediated reactions to asparagine. Up to 27 % of women exposed to 6 or more cycles of carboplatin present with immediate HSR and 50 % of these reactions are anaphylactic. BRCA1/2 mutations have been found to be more common among patients who develop immediate reactions to carboplatin and these reactions, including anaphylaxis, have been successfully addressed by rapid drug desensitization (RDD). We sought to evaluate the HLA profile of patients who presented an initial carboplatin-induced severe hypersensitivity reaction including anaphylaxis, had a positive skin test and were treated with the BWH rapid drug desensitization 3–4 bags protocol.

**Materials and methods:** HLA ABC DR DQ DP typing was conducted for a cohort of Caucasian ovarian cancer patients (n = 11) with a history of grade 2 or 3 type I HSR to carboplatin undergoing RDD and a control group of Caucasian ovarian cancer patients treated with carboplatin, who tolerated 8 or more cycles (n = 12). Allele carriage amongst cases and controls was compared using Fisher’s exact test. The *Haploblocks* program was used to generate cobygram visuals of HLA co-carriage.

**Fig. 6:**
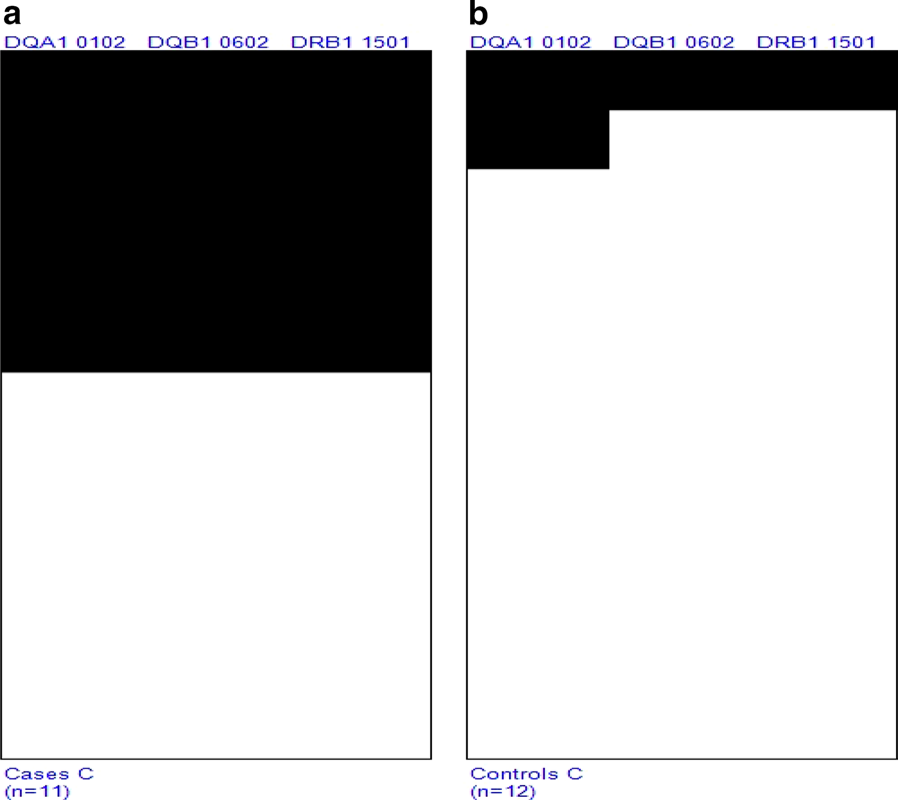
Overrepresentation of DQA1*01:02-DQB1*06:02-DRB1*15:01 haplotype in the allergic group

**Results:** Among allergic patients, 36 % (4/11) had grade 2 and 64 % (7/11) grade 3 initial HSR. All successfully tolerated the RDD and were able to complete their treatment plans. We found that the HLA-DRB1*15:01 allele was more prevalent among allergic patients (5/11, 45 %) than among the control group (1/12, 8.3 %) (p = 0.06). Cobygram analysis suggests that the HLA class II association may extend to the DQA1*01:02-DQB1*06:02-DRB1*15:01 haplotype (Fig. 6).

**Conclusions:** In this Caucasian cohort of ovarian cancer patients, a specific HLA class II allele, DRB1*15:01, was suggested to be associated with immediate hypersensitivity to carboplatin. Further carboplatin HSR cases and carboplatin tolerant controls are currently been accrued to verify the validity of this association and assess its extension to the DQA1*01:02-DQB1*06:02-DRB1*15:01 haplotype.

**Keywords:** HLA genotyping; Rapid drug desensitization; Carboplatin; IgE-mediated reaction; Drug allergy

## Poster Walk 17: In vivo diagnosis + sIgE (P147–P154)

### P147 Absence of specific Ig-e against beta-lactams 9 months after an allergic reaction to amoxicillin-clavulanic acid

#### Elisa Boni, Marina Russello, Marina Mauro

##### Hospital Sant’Anna, Como, Italy

**Correspondence:** Elisa Boni

*Clinical and Translational Allergy* 2016, **6(Suppl 3)**:P147

**Background:** In case of immediate reactions to beta-lactams, allergological work up consists of detection of specific Immunoglobulin-E (Ig-E) against beta-lactams *inserum* and skin tests, i.e. prick test and intradermal tests with major and minor antigenic determinants of penicillin, amoxicillin, ampicillin and benzilpenicillin. Levels of specific Ig-E decrease after the reaction and they are no more detectable after some years. It is recommended to perform in vivo and in vitro tests after 3 weeks and no later than 6–12 months after the reaction. Sensitivity of tests then decreases and false negative result may occur. In case of remote history of immediate reaction to beta-lactam, if tests are negative, a provocation test with the culprit drug is necessary and should be followed by repetition of skin tests.

**Report:** We report the case of a woman aged 61 years who experienced a systemic reaction (urticaria, vomit, diarrhea, fatigue) 15 min after intake of amoxicillin/clavulanic acid. Four months later, she underwent a visit in our Allergy Unit and serological and skin tests were scheduled.

Serological specific Ig-E against penicilloyl G, penicilloyl V, ampicillin, amoxicillin, cefaclor as well as skin tests gave negative result. They were performed respectively 5 and 9 months after the reaction. A drug provocation test (DPT) with amoxicillin was then performed and was considered positive for the occurrence of generalized pruritus 20 min after the intake of the last dose (cumulative dose 875 mg). Skin tests were repeated with positive results to both amoxicillin and ampicillin, at the concentration of 1 and 20 mg/ml, respectively.

**How this report contributes to current knowledge:** Specific Ig-E levels against beta-lactams decrease after immediate-type reactions. Guidelines recommend to perform allergological work up within 6–12 months. Our case shows how this period might be too long and false negative tests can occur even if performed within one year. DPT should always follow negative skin tests to obtain a correct diagnosis.

**Consent:** Written informed consent was obtained from the patient for publication of this abstract and any accompanying images.

### P148 Drug provocation tests in suspected opioid allergy

#### Kok Loong Ue, Krzysztof Rutkowski

##### Department of Allergy, Guy’s and St Thomas’ NHS Foundation Trust, London, United Kingdom

**Correspondence:** Kok Loong Ue

*Clinical and Translational Allergy* 2016, **6(Suppl 3)**:P148

**Background:** There are no good epidemiological data regarding opioid allergy (OA). Many side effects can occur with this group of drugs. These are often misinterpreted as an IgE-mediated OA. As a consequence, many patients receive an unsubstantiated diagnosis of OA and avoid opioid unnecessarily. Diagnosis of OA can be challenging as skin tests (ST) and opioid-specific IgE have a poor predictive value. Drug provocation tests (DPT) remain the gold standard for the diagnosis of OA. However, there are almost no studies of opioid DPT and no validated opioid DPT protocols.

**Materials and methods:** 30 suspected cases of OA (12 morphine, 18 codeine) were studied using detailed history and oral DPT. Opioid ST and specific IgE were not performed. Standardised local protocols were used. For morphine: 2, 3, and 5 mg morphine sulphate oral solution (10 mg/5 ml) was administered at 30 min interval with 120 min monitoring. For codeine: 10, 20 and 30 mg codeine phosphate syrup (25 mg/5 ml) was administered at 30 min interval with 60 min of monitoring. All DPT were performed on a specialist allergy unit.

**Results:** 25 females and 5 males (median age of 46.5) experienced various symptoms reported as OA. Opioids were prescribed mainly for analgesia. 20 patients (67 %) had cutaneous symptoms: angioedema: 5, urticaria: 4, angioedema and urticaria: 2, unspecified rash: 5, angioedema, unspecified rash and pruritus: 3, angioedema and unspecified rash: one. 11 patients (37 %) experienced symptoms suggestive of anaphylaxis: dyspnoea, tachycardia, hypotension, dizziness and collapse. 2 of these (18 %) experienced cutaneous symptoms too. Other non-specific symptoms included nausea: 2, paraesthesia: 2, tremor: 1, anxiety: 1. DPT were negative in 23 patients (77 %). 6 patients (20 %) had symptoms of intolerance: pruritus, nausea, dizziness, tremor to morphine (2) and codeine (4). Only 3 % of DPT were positive: 1 patient experienced angioedema with codeine.

**Conclusions:** DPT-confirmed OA appears to be very rare (3 %). Most symptoms misinterpreted as OA could be due to a dose-dependent mast cell degranulation, genetic polymorphism of CYP2D6 gene or the underlying acute illness. Morphine and codeine DPT are a safe and reliable diagnostic tool. Our ongoing multi-centre study will endeavour to validate this approach in a larger cohort of suspected OA patients (including other opioids) and produce standardised DPT protocols to improve the diagnosis of OA in Europe and worldwide.

**Keywords:** Anaphylaxis; Codeine; Drug provocation test; Morphine; Opiod allergy

### P149 Improvement to the specific IgE cut-off in the assess of β-lactamic allergy

#### Victor Soriano Gomis, Jorge Frances Ferre, Angel Esteban Rodriguez, Vicente Cantó Reig, Javier Fernandez Sanchez

##### Universitary General Hospital of Alicante, Alicante, Spain

**Correspondence:** Victor Soriano Gomis

*Clinical and Translational Allergy* 2016, **6(Suppl 3)**:P149

**Background:** Specific IgE against β-lactams by CAP FEIA system (Thermo Scientific^®^) used to use the cut-off of 0.35 kU/l, but now we can get a detection limit of the assay of 0.10 kU/l. We pretend to evaluate if this change of cut-off from 0.35 to 0.10 kU/l improves the sensitivity without compromise its specificity.

**Materials and methods:** 74 patients older than 18 years evaluated in the allergy outpatient clinic since January 2011 to April 2014 were studied. They were classified as allergic by history and skin tests positive to β-lactamic antibiotics. Specific IgE against penicilloyl G, penicilloyl V, amoxicilloyl and ampicilloyl were analyzed by CAP FEIA system (Thermo Scientific^®^). With these IgE values and positive or negative diagnosis of allergy, sensitivity and specificity of both cut-offs (0.35 and 0.10 kU/l), the ROC curves were obtained. In addition, the AUC value (with their confidence interval) was calculated. The SPSS (v19.0) and “Analyse-it”-Excel were used.

**Results:** Tables 5, 6, 7.

**Table 5:** ROC results

Specific IgE	AUC value	CI 95 %
Penicilloyl G	0.780	0.66–0.90
Penicilloyl V	0.748	0.638–0.858
Ampicilloyl	0.783	0.674–0.892
Amoxicilloyl	0.844	0.758–0.930

**Table 6:** Sensitivity and specificity with the 0.10 kU/l cut-off

Specific IgE	Sensitivity (%)	Specificity (%)
Penicilloyl G	55.6	85.7
Penicilloyl V	47.1	80.0
Ampicilloyl	74.2	69.0
Amoxicilloyl	74.2	74.4

**Table 7:** Sensitivity and specificity with the 0.35 kU/l cut-off

Specific IgE	Sensitivity (%)	Specificity (%)
Penicilloyl G	33.3	94.6
Penicilloyl V	23.5	97.5
Ampicilloyl	35.5	97.6
Amoxicilloyl	45.2	93.0

**Conclusions:** The cut-off of 0.10 kU/l may be more appropriate to use in the clinical practice because it improves the correct classification of b-lactams allergy patients. The amoxicilloyl obtains the best performance with this new cut-off.

### P150 Initial false negative specific IgE to gelatin in a patient with gelatin-induced anaphylaxis

#### Christine Breynaert^1^, Erna Van Hoeyveld^2^, Rik Schrijvers^1^

##### ^1^Laboratory of clinical immunology, department of microbiology and immunology, KU Leuven, Leuven, Belgium; ^2^Laboratory medicine, Immunology, University Hospitals, KU Leuven, Belgium, Leuven, Belgium

**Correspondence:** Rik Schrijvers

*Clinical and Translational Allergy* 2016, **6(Suppl 3)**:P150

**Background:** The time of determination of specific IgE (sIgE) to potential culprit drugs, as an aid in the diagnosis of peri-operative anaphylaxis, is not well defined for all drugs.

**Report:** A 62-year old man experienced an anaphylaxis within 15 min after receiving a gelatin-containing plasma expander during general anesthesia for hip surgery after a high velocity trauma. Decontamination was performed with chlorhexidine, and propofol, lidocaine, sufentanil, rocuronium, dexamethasone, was adminstered 2h15 and cephazolin 1h45 prior to the event. The gelatin infusion was stopped and treatment with epinephrine, norepinephrine, promethazine, hydrocortisone, and saline fluid expansion installed. Serum tryptase was transiently increased (95.7 ng/dl 1h30 after the onset of the reaction vs 6.0 ng/dl > 24 h after the event). ImmunoCAP sIgE (Thermofisher—Phadia, Sweden) to gelatin, galactose-alpha-1,3-galactose, ethylene oxide, latex, and chlorhexidine was negative on the sample obtained 1h30 after the event, and independently confirmed. However, repeat sampling 16 days after the event with a serial dilution of the gelatin-containing plasma expander, mimicking the timing of the initial testing, demonstrated the inhibition of sIgE determination with a 50 % inhibitory concentration (IC50) of 0.02 %. No serum before the anaphylaxis was available to confirm a pre-existing sensitization. Skin testing 4 weeks after the event was positive for the 4 % gelatin-containing plasma expander (3 and 5 mm wheal diameter after skin prick testing at a 1:10 and 1:1 dilution respectively) and negative for chlorhexidine, latex, and cephazoline.

**How this report contributes to current knowledge:** A rare case of IgE-mediated gelatin allergy is reported. We hypothesized that the initial sIgE for gelatin was false negative due to competition of infused gelatin with the gelatin-immunoCAP assay rather than a boostin phenomenon had taken place, suggesting careful interpretation of sIgE determinations very early after the event, at least in the case of gelatin-sIgE.

**Consent:** Written informed consent was obtained from the patient for publication of this abstract and any accompanying images.

### P151 Inmediate reactions to beta-lactam antibiotics: pattern of skin test response over the time

#### Jose Julio Laguna Martinez^1^, Rosario Gonzalez Mendiola^1^, Javier Dionicio Elera^1^, Cosmin Boteanu^1^, Aranzazu Jimenez Blanco^1^, Marta Del Pozo^1^, Raquel Fuentes Irigoyen^2^

##### ^1^Allergy Unit. Hospital Central de la Cruz Roja, Madrid, Spain; ^2^Pharmacy Service. Hospital Central de la Cruz Roja, Madrid, Spain

**Correspondence:** Jose Julio Laguna Martinez

*Clinical and Translational Allergy* 2016, **6(Suppl 3)**:P151

**Background:** Beta-lactam (BL) antibiotics are the drugs most frequently involved in IgE-mediated allergic reactions. Skin testing is the most widely used diagnostic method to evaluate IgE mediated reactions to BL. Skin test respond to penicillin determinants vary in different populations over time related with pattern of consumption. But precise studies The aim of our work is to study the variation of the skin response over long time in a well-defined series of allergic to BL.

**Materials and methods:** All patients submitted to our centre, in 10 year period (1999–2009) with a compatible history of beta-lactam allergy underwent allergy work-up according to the ENDA protocols for immediate reactions.

Skin tests (prick and intradermal) with the classical penicillin reagents [Allergopharma Reinbeck, Germany (1999–2005)] and from 2005 (Diater reagents DAP, Madrid, Spain): penicilloyl polylysine (PPL), minor determinants mixture (MDM: benzylpenicillin and sodium benzylpenicilloate) and benzylpenicillin (penicillin G) and Semi-synthetic penicillins (amoxicillin, amoxicilin/clavulanic acid and ampicillin) were also systematically tested, as well as any other suspected beta-lactam. In the case of negative results, we perform provocation tests with the suspected drug. Considering the temporal variation for immediate reactions in terms of skin test positivity, Patients were classified according to the year they referred the reaction, in four periods (>1980, 1980–1990, 1991–2000 and >2001–2009)

**Results:** From a total of 2716 patients initially evaluaed, 296 were finale confirmed as inmediate reactions to Betalactamic. A total of 247 were diagnosed by skin testing, this represents 83 % of total (247/296).

Considering the temporal variation for immediate reactions in terms of skin test positivity, the analysis of the AX determinants showed that there was a gradual increase over the years that varied from 38.5 to 81.2 %. Statistical analysis confirm a strong signification p < 0.0001. In parallel a decreased of antigenic determinants of penicillin (including PPL, MDM and Benzylpenicillin) from 30.8 to 12.7 % also statistic significance p < 0.007.

**Conclusions:** Our study confirm that the variation of beta-lactam pattern consumption modify the skin test response in a long series over 10 years.

Strong statistical significance of amoxicillin skin tests rising p < 0.001 correlates with the progressive increase in amoxicillin consumption since 2001.

**Keywords:** β-Lactams; Amoxicillin; Skin test; Allergy; Inmediate reaction

### P152 New fluorescent dendrimeric antigens for the evaluation of dendritic cell maturation as a test to detect allergy reactions to amoxicillin

#### Daniel Collado^1^, Yolanda Vida^1^, Francisco Najera^1^, Ezequiel Perez-Inestrosa^1^, Pablo Mesa-Antunez^1^, Cristobalina Mayorga^2^, María José Torres^2^, Miguel Blanca^2^

##### ^1^University of Malaga, Malaga, Spain; ^2^Allergy Service, Carlos Haya, Malaga, Spain

**Correspondence:** Daniel Collado

*Clinical and Translational Allergy* 2016, **6(Suppl 3)**:P152

**Background:** ln order to emulate the recognition process working in vivo, much effort have been made to prepare hapten(drug)-carrier(protein) conjugates attempting to work like the antigen responsible for the allergic drug reaction.

**Materials and methods:** In our efforts to exploit the dendrimer properties in the interaction with the immunological system, we have prepared a series of Dendrimeric:Antigens (DeAn), to study the dendritic cell (DC) maturation as a test to detect allergy reactions to amoxicillin.

Recently our research group developed a new kind of dendrimer, called BAPAD, that we have used in this work to obtain the dendrimeric moiety of the target molecule. To this avail we synthesized a generation two BAPAD dendrimer using cystamine as core. Then, the free amine groups on the surface of the dendrimer were functionalized with an amoxiciloyl group (AXO). The fluorescent DeAn has been fully characterized by NMR and MS techniques, and their fluorescent properties well established in aqueous biological media using confocal microscopy. The fluorescent dendron (De) without the haptenic moieties at the periphery has been also obtained and fully characterized as a control assay. The fluorescent DeAn and De was used in dissolution and supported in solid surface (cellulose disk). The solid conjugates were immunologically evaluated by RAST inhibition using sera from 7 patients allergic to amoxicillin. DC from four amoxicillin allergic patients have been incubated with fluorescent DeAn and De and studied with a flow cytometer to determine whether or not the cells were able to uptake both compounds.

**Results:** Flow cytometry and confocal microscopy show that these dendrimeric structures interact with DC and are internalized by them to the cellular cytoplasm. The maturation of DC was tracked by by flow cytometry. In all cases, low maturation induced by DeAn in allergic patients is observed. This effect can be due to two factors: (1) that the concentration of compound that enters the DC is low and (2) that the hapten density presented in DeAn is low. The RAST inhibition studies of solid supported DeAn showed a higher RAST inhibitions values for allergic patients compare with negatives controls. This value was also compared with solid surfaces conjugated with PLL-AXO showing similar results.

**Conclusions:** These dendrimeric structures assayed in RAST inhibition studies letting us check that inhibition occurred, so recognition existed between IgE of patients allergic to amoxicillin and DeAn structures.

**Keywords:** Confocal microscopy; Dendritic cell; Maturation

### P153 Positive skin test or positive specific IgE to penicillin does not predict penicillin allergy

#### Line K. Tannert^1^, Charlotte G. Mortz^2^, Per Stahl Skov^3^, Carsten Bindslev-Jensen^2^

##### ^1^Odense Research Center for Anaphylaxis, Odense C, Denmark; ^2^Department of Dermatology and Allergy Center, Odense C, Denmark; ^3^Odense Research Center for Anaphylaxis, Odense C/Copenhagen, Denmark

**Correspondence:** Line K. Tannert

*Clinical and Translational Allergy* 2016, **6(Suppl 3)**:P153

**Background:** Diagnosis of penicillin allergy is based on case history, skin testing (ST, prick and intracutaneous tests) and measurement of specific IgE (s-IgE) to a penicillin and challenge with penicillin. If ST or s-IgE is positive, the patient is classified as allergic to penicillin according to the European Network of Drug Allergy guidelines and challenge is omitted. Therefore, the true sensitivity and specificity of ST and s-IgE are presently not known.

The aim of this study was to investigate the clinical relevance of a positive skin test and s-IgE to penicillin.

**Materials and methods:** Forty-four patients with a positive ST and/or s-IgE were included; ST with penicillin was done and s-IgE was measured in all 44 patients at the time point designated **T**_**0**_. Challenge with the culprit penicillin was performed immediately hereafter although abstained in patients with recent anaphylaxis to penicillin (n = 8), systemic reactions to ST (n = 3) or development of delayed positive ST (n = 8). These were classified as allergic to penicillin; thus, 25 patients were challenged.

18 of the patients had been evaluated previously (**T**_**−1**_) and reproducibility of the results were compared to **T**_**0**_ and again 4 weeks post challenge (**T**_**1**_).

**Results:** Nine of the 25 challenged patients were positive. There was a significantly increased probability of being allergic to penicillin if both skin test and s-IgE were positive at **T**_**0**_ (p = 0.007). Positive ST or s-IgE alone did not predict penicillin allergy (p = 0.313/p = 0.051).

Among the 18 patients repeatedly tested, only 12/26 (46.2 %) of positive skin tests at **T**_**−1**_ were reproducibly positive at **T**_**0**_, and only one further ST became positive at **T**_**1**_. For s-IgE, 14/24 (58.3 %) of positive measurements were still positive at **T**_**0**_ and seven further became positive at **T**_**1**_, although s-IgE levels at **T**_**0**_ and **T**_**1**_ did not differ significantly (p = 0.599).

**Conclusions:** In this study, the best predictor for a positive penicillin challenge was history combined with both positive ST and s-IgE. There was a relatively low reproducibility of a previously positive ST and s-IgE.

### P154 Significance of skin testing and in vitro-analysis of neuromuscular blocking agents in diagnosis of perioperative drug hypersensitivity: evaluation of a negative control population

#### Wolfgang Pfützner, Hannah Dörnbach, Johanna Visse, Michele Rauber, Christian Möbs

##### Department of Dermatology and Allergology, Philipps University Marburg, Marburg, Germany

**Correspondence:** Wolfgang Pfützner

*Clinical and Translational Allergy* 2016, **6(Suppl 3)**:P154

**Background:** Perioperative drug hypersensitivity (PDH) presents a major problem resulting in unexpected, often severe anaphylactic reactions. Diagnosis of anaphylaxis during anaesthesia mainly relies on skin testing, since assays for the detection of IgE-antibodies are not available and provocation tests are not feasible for most of the involved drugs. Neuromuscular blocking agents (NMBAs) are recognized as the most prevalent elicitors of PDH, however, skin tests with NMBAs might result in false-positive results due to unspecific, non-IgE-mediated release of histamine.

**Materials and methods:** To further investigate this subject, we evaluated the potential of NMBAs eliciting positive results in individuals with known NMBA-tolerance in both skin prick (SPT) and intracutaneous tests (ICT) and compared these with the reactivity in basophil activation tests (BAT). Eleven individuals [5 females, 6 males; 25–60 years old, median (m) = 50] without known allergy to NMBAs, but tolerant to a recently perioperative applied NMBA were included in this study. The NMBAs Suxamethonium, Rocuronium, Cis-Atracurium and Mivacurium were tested by both titrated SPT and ICT, and NMBAs eliciting a positive skin reaction were further analyzed by BAT with the individuals’ serum.

**Results:** SPT yielded positive results in 0/11 participants for Suxamethonium, Rocuronium and Cis-Atracurium and in 2/11 for Mivacurium. ICT was positive in 2/11 for Suxamethonium, 9/11 for Rocuronium, 8/11 for Cis-Atracurium and in 11/11 for Mivacurium. BAT analysis confirmed the positive skin tests in only one individual, while it was negative for the NMBAs in all others.

**Conclusions:** These findings show that skin test results with NMBAs have to be carefully interpreted regarding their clinical relevance and the implementation of more reliable test systems would be helpful for diagnosis of PDH.

**Keywords:** Perioperative anaphylaxis; Anaesthesia; Neuromuscular blocking agents; Skin test; Basophil activation test

## Poster Walk 18: In vitro/ex vivo (P155–P158, P160–P164)

### P155 Diagnostic value of the lymphocyte toxicity assay (LTA) and the in vitro platelet toxicity assay (IPTA) for β-lactam allergy

#### Abdelbaset A. Elzagallaai, Lindsey Chow, Awatif M. Abuzgaia, Michael J. Rieder

##### Western University, London, Canada

**Correspondence:** Abdelbaset A. Elzagallaai

*Clinical and Translational Allergy* 2016, **6(Suppl 3)**:P155

**Background:** β-lactam antibiotics (BLAs) are the drugs most associated with immune-mediated hypersensitivity reactions (drug allergy). They can elicit all types of allergic reactions i.e., types I, II, III and IV. Although the immediate IgE-mediated reaction has been well studied, the pathophysiology of non-immediate hypersensitivity reactions is not well understood. Cross-reactions among BLAs with different side chains are variable. The diagnosis and prediction of BLAs-induced allergic reactions is challenging and based mostly on clinical history. The lymphocyte toxicity assay (LTA) and the in vitro platelet toxicity assay (*i*PTA) are in vitro tests with a potantial value for diagnosis and prediction of drug allergy. Their value in diagnosis of reactions to BLAs, however, is still unknown. This work is an attempt to evaluate the performance of in vitro toxicity testing for diagnosis of different types of allergy to BLAs and to investigate the their different underlying pathophysiology.

**Materials and methods:** One hundred and seventy individuals (85 drug allergy-suspected patients and 85 healthy volunteers) were included in this study. Patients were identified from clinical records and included using a rigorous internationally recognized inclusion criteria based on their clinical presentation and available diagnostic investigations (i.e., skin testing and RAST). The LTA and *i*PTA tests were performed after resolution of the reaction symptoms. Data was expressed as percentage of cell death compared to control (vihicle without the drug) after incubation with the suspected drug in presence of rat microsomes (MICs).

**Results:** Patients were grouped according to their exhibited symptoms into 3 groups constituted of 8 patients type I, 28 patients type III and 49 patients type IV. Patients with type IV reactions exhibited higher degrees of cell death than the other 2 groups (p < 0.05) with type I patients exhibiting the lowest degree of cell death among all groups.

**Conclusions:** Using the in vitro toxicity testing (LTA and *i*PTA) we were able to identify patients susceptible to develop allergy to BLAs. In addition, cells isolated from patients with different types of reactions exhibit variable degrees of cell death, which indicate possible distinct pathophysiological mechanisms. The LTA and *i*PTA can be a useful tool to both diagnose drug allergy to BLAs and investigate its underlying pathophysiology (Table 8).

**Table 8:** Diagnostic value of the lymphocyte toxicity assay (LTA) and the in vitro platelet toxicity assay (IPTA) for ß-Lactam allergy

Characteristic	Value
Sex [female/male; n (%)]	53/31 (63.1/36.9)
Age [mean; year (range)]	24 (1–88)
Type of reaction [n (%)]	
Type I	8 (9.4)
Type III	28 (32.9)
Type IV	49 (57.6)
Total	85 (100)

**Keywords:** Drug allergy; Drug hypersensitivity; In vitro diagnosis

### P156 Enzyme linked immunospot assay used in the diagnosis of severe cutaneous adverse reactions to antimicrobials

#### Alec Redwood^1^, Jason Trubiano^2^, Rebecca Pavlos^1^, Emily Woolnough^2^, Kaija Stautins^1^, Christina Cheng^2^, Elizabeth Phillips^3^

##### ^1^Institute of Immunology and Infectious Diseases, Murdoch University, Perth, Australia; ^2^Department of Infectious Diseases, Alfred Health, Melbourne, Australia; ^3^Vanderbilt University Medical Center, Nashville, United States

**Correspondence:** Alec Redwood

*Clinical and Translational Allergy* 2016, **6(Suppl 3)**:P156

**Background:** The ability to define drug causality in cases of Severe Cutaneous Adverse Reactions (SCAR) where multiple antimicrobials are implicated remains problematic. We describe a case of toxic epidermal necrolysis (TEN) in the setting of multiple implicated antimicrobials and the utility of T-cell enzyme linked immunospot assay (ELISpot) to define antimicrobial causality.

**Report:** A 20-year old man was admitted to a tertiary referral trauma centre for management of wound sepsis and femoral stump osteomyelitis in the setting of recent below-knee amputation following a high-speed motorbike accident. Whilst receiving escalating antimicrobial treatment, for bacteraemia and fevers >38.3 °C, he developed a blistering rash involving >30 % of body surface area (BSA) associated with a positive Nikolsky sign, consistent with TEN. Multiple antimicrobials were administered prior to onset of TEN, four of which—vancomycin, meropenem, linezolid and teicoplanin—were temporally associated with the onset of TEN.

**How this report contributes to current knowledge:** We sought to use cellular assays, IFN-γ ELISpot and flow cytometry, to identify the causative agent of TEN in a patient receiving multiple antibiotics. Following informed consent, patient whole blood was collected on day 4 post onset of TEN. PBMCs were extracted and cryopreserved. HLA ABC DR DQ DP typing was performed and the PBMCs were used for ex vivo ELISpot testing. PBMCs were also incubated with candidate drugs for 18–20 h at 37 °C in 5 % CO_2_ and assessed by flow cytometry for upregulation of the T cell activation marker CD137.

Patient HLA was HLA-A*01:01 and 02:02, HLA-B*37:01 and 41:01, HLA-C*10:01 and 17:01, HLA-DPB1*10:01, HLA-DQA1*01:01 and 02:01, HLA-DQB1*02:02 and 05:01, HLA-DRB1*07:01 and 10:01. Positive ELISpot responses were reproducibly produced only to the glycopeptide antibiotic teicoplanin, with up to 300 SFU/million cells. ELISpot data were confirmed by flow cytometry analysis, where only teicoplanin induced up-regulation of the activation marker CD137 (0.3 % of total CD8+ T-cells). All other antibiotics including vancomycin and meropenem were negative by ELISpot and flow cytometry.

We believe this to be the first reported use of T-cell ELISpot to assign isolated teicoplanin causality to TEN.

**Consent:** Written informed consent was obtained from the patient for publication of this abstract and any accompanying images.

### P157 Evaluation of in vitro diagnostic methods for identifying the culprit drugs in drug hypersensitivity

#### Kenichi Kato^1^, Hiroaki Azukizawa^2^, Takaaki Hanafusa^3^, Ichiro Katayama^1^

##### ^1^Department of Dermatology, Osaka University, Osaka, Japan; ^2^Department of Dermatology, Nara Medical University, Nara, Japan; ^3^Department of Dermatology, Tokyo Medical and Dental University, Tokyo, Japan

**Correspondence:** Kenichi Kato

*Clinical and Translational Allergy* 2016, **6(Suppl 3)**:P157

**Background:** The drug-induced lymphocyte stimulation test (DLST), or lymphocyte transformation test (LTT), is used to identify the culprit drug in cases of cutaneous adverse drug reactions (cADR). While DLST is widely used as in vitro diagnostic tool, its sensitivity and specificity are unsatisfactory. Determination of antigen-specific IFN-γ production by enzyme-linked immunospot assay (conventional IFNγ-ELISpot) is well-established diagnostic method for tuberculosis infection, and recent reports suggested that drug-induced conventional IFNγ-ELISpot is useful for identifying the culprit drug of cADR cases. The aim of this study was to establish a novel diagnostic method for identifying the culprit drug in cADR patients through the efficient detection of the drug-specific IFN-γ production by IFNγ-ELISpot.

**Materials and methods:** Ten cases of cADR caused by clinically convincing culprit drugs were enrolled in this study. Peripheral blood mononuclear cells (PBMCs) from all 10 patients were used for both DLST and drug-induced conventional IFNγ-ELISpot. In addition, drug-induced IFNγ-ELISpot was also performed by using PBMCs which were non-specifically stimulated with monoclonal antibodies for 7 days before exposing culprit drugs (modified IFNγ-ELISpot) in all cases.

**Results:** Drug-induced IFN-γ production was detected by modified IFNγ-ELISpot in 5 patients of which DLST and conventional IFNγ-ELISpot were both negative. Moreover, IFN-γ secretion was observed by modified IFNγ-ELISpot in all 4 patients of which DLST were positive.

**Conclusions:** Modified IFNγ-ELISpot using expanded PBMCs is more sensitive than conventional IFNγ-ELISpot for detecting drug-induced IFN-γ production. Therefore, this novel IFNγ-ELISpot could be a useful in vitro tool for identifying culprit drugs in cADR cases.

### P158 Ex-vivo expanded skin-infiltrating T cells from severe drug eruptions are reactive with causative drugs: a possible novel method for determination of causative drugs

#### Toshiharu Fujiyama^1^, Hideo Hashizume^2^, Takatsune Umayahara^1^, Taisuke Ito^1^, Yoshiki Tokura^1^

##### ^1^Hamamatsu University School of Medicine, Hamamatsu, Japan; ^2^Shimada City Municipal Hospital, Shimada, Japan

**Correspondence:** Toshiharu Fujiyama

*Clinical and Translational Allergy* 2016, **6(Suppl 3)**:P158

**Background:** Stevens–Johnson syndrome (SJS), toxic epidermal necrolysis (TEN) and drug-induced hypersensitivity syndrome/drug reaction with eosinophilia and systemic symptoms (DIHS/DRESS) are life-threatening adverse drug reactions. For prevention of relapse, it is mandatory to determine the causative drug. Patch test and lymphocyte stimulation test (LST; also known as DLST in Japan) are frequently used for this purpose, but their positivity ratios are not sufficiently high. We sought to explore a novel method using skin-infiltrating T cells for determination of causative drugs because it is considered that antigen-specific T cells infiltrate in skin lesions of the severe drug eruptions and participate in its pathogenesis.

**Materials and methods:** We expanded skin-infiltrating T cells from 4-mm biopsied lesional skin samples of severe drug eruption using anti-CD3/CD28 antibodies and IL-2. More than 10^7^ T cells/specimen were obtained by 2-week cultivation. To investigate their cytokine production by in vitro stimulation with causative drugs, the expanded T cells were co-cultured with drugs peripheral blood mononuclear cells (PBMCs) from the same patient, and IFN-g production was assessed by ELISpot assay. Moreover, to see the drug-induced T-cell proliferation, the expanded T cells were labeled with CFSE and cultured with drugs and X-ray-irradiated PBMCs for a week, and the proliferation was assessed by flow cytometry. The cytokine profile of the cells which proliferated in response to drugs was also assessed by flow cytometry.

**Results:** The ELISpot assay showed a significantly high number of T cells produced IFN-g by drug stimulation as compared to no addition control. The CFSE assay revealed that both CD8+ and CD4+ T cells proliferated in response to causative drugs in SJS/TEN and DIHS/DRESS. Notably, the majority of CD8+ T cells proliferating to causative drugs expressed IFN-g in SJS/TEN.

**Conclusions:** Our study suggests that the use of ex vivo expanded skin-infiltrating T cells can yield a novel method for determination of causative drugs in the severe drug eruptions.

**Keywords:** T cells; IFN-γ

### P160 In vitro release of IL-2, IL-5 and IL-13 in diagnosis of patients with delayed-type nickel hypersensitivity

#### Mira Silar^1^, Mihaela Zidarn^1^, Helena Rupnik^2^, Peter Korosec^1^

##### ^1^University Clinic for Respiratory and Allergic Diseases, Golnik, Slovenia; ^2^ARSDERMA, Ljubljana, Slovenia

**Correspondence:** Mira Silar

*Clinical and Translational Allergy* 2016, **6(Suppl 3)**:P160

**Background:** T cells play a major role in delayed-type of hypersensitivity reactions. Their reactivity can be assessed by lymphocyte transformation test or upregulation of cell surface activation markers, and those tests are of limited practicability or diagnostic sensitivity. Some previous report suggested that in vitro secretion of cytokines by peripheral blood mononuclear cells (PBMC) could be a promising tool for improved detection of T-cell sensitization. For that reason we wanted to test this methodology in patients with well-defined delayed-type nickel hypersensitivity.

**Materials and methods:** PBMC of 10 nickel hypersensitive patients and 9 healthy controls were incubated for 48 h with two different concentration of NiSO_4_ × 6H_2_O (0.5–5 μg/ml). IL-2, IL-5, IL-13 and IFN-γ concentrations were measured in supernatants with multiplex flow cytometry CBA Flex Array.

**Results:** We showed a significantly increased secretion of IL-2 (median 182 vs 3 ng/ml), IL-5 (9 vs 0 ng/ml), IL-13 (36 vs 0.5 ng/ml) in response to NiSO_4_ × 6H_2_O in patients nickel hypersensitivity when compared with healthy controls. The response in the patients was concentration dependent. No difference was evident for IFN-γ. The ROC curve analysis demonstrated the highest AUC of 0.99 for IL-2 and IL-13, followed by AUC of 0.91 for IL-5.

**Conclusions:** The measurement of IL-2 and IL-13 appearing to be a superb approach to identify antigen reactive T cells in the peripheral blood of patients with the hypersensitivity reactions. This cytokine approach could be now tested on the clinical important issue of the diagnosis of the delayed-type hypersensitivity reactions to drugs.

### P161 Single cell analysis of drug responsive T cells; identification of candidate drug reactive T cell receptors in abacavir and carbamazepine hypersensitivity

#### Alec James Redwood^1^, Kaija Strautins^1^, Katie White^2^, Abha Chopra^1^, Katherine Konvinse^2^, Shay Leary^1^, Rebecca Pavlos^1^, Simon Mallal^2^, Elizabeth Phillips^2^

##### ^1^Institute for Immunology and Infectious Diseases, Murdoch University, Perth, Australia; ^2^Vanderbilt University Medical Center, Nashville, United States

**Correspondence:** Alec James Redwood

*Clinical and Translational Allergy* 2016, **6(Suppl 3)**:P161

**Background:** T cell mediated drug hypersensitivity requires three essential components, a target drug, a scaffold for the drug (MHC class I or class II) and a pathogenic T cell receptor (TCR). For several drugs including, abacavir and carbamazepine, two components of this trimolecular complex have been defined. For abacavir hypersensitivity reaction (HSR) the associated MHC is HLA-B*57:01 and for carbamazepine induced Stevens–Johnson syndrome/toxic epidermal necrolysis (SJS/TEN), the associated MHC is HLA-B*15:02. In the former instance the association of drug with MHC has been further defined with the successful resolution of the crystal structure of the abacavir complexed to HLA B57:01.

To fully understand T cell mediated hypersensitivity reactions the nature of the T cells involved and ultimately the mechanism by which the TCR interacts with MHC plus drug must be elucidated. This study seeks to sequence and clone the TCR mediating drug hypersensitivity in response to a series of drugs.

**Materials and methods:** Working from a cohort of cryopreserved PBMCs on HLA-typed patients with confirmed histories of T-cell mediated drug hypersensitivities (abacavir hypersensitivity and carbamazepine SJS/TEN and DRESS) we performed flow cytometry, single cell cloning and single cell TCR sequencing and used these to develop and interrogate T-cell responses. PBMC samples with confirmed T-cell reactivity (ELISpot) were treated with drug for 18–20 h. Upregulation of the activation marker CD137 was used to sort drug reactive CD8+ or CD4+ T cells. T-cells lines or clones were produced and individual T-cells were sorted for single or bulk TCR sequencing.

**Results:** We have developed a pipeline for the identification of pathogenic TCRs. T-cell lines maintained reactivity to candidate drugs. TCR sequencing from abacavir HSR patients (n = 3) identified no public TCR, rather multiple putative pathogenic TCRs have been identified. For carbamazepine induced SJS/TEN (n = 1) and DRESS (n = 1) we have also identified multiple putative pathogenic TCR.

**Conclusions:** Several candidate pathogenic TCRs have been identified in patients with hypersensitivity to abacavir or carbamazepine. These putative TCRs will be cloned into Jurkat T-cell lines to determine if multiple T-cell clones contribute to hypersensitivity or if only a single clone in each patient is pathogenic. Ultimately these cloned TCR will be used to define the crystal structure of the trimolecular complex of drug/MHC/TCR.

**Keywords:** T cell receptor; Abacavir; Carbamazepine

### P162 Specificity and sensitivity of LTT in DRESS: analysis of agreement with the Spanish pharmacovigilance system probability algorithm

#### Rosario Cabañas^1^, Elena Ramirez^2^, Ana María Fiandor^3^, Teresa Bellón^4^

##### ^1^Servicio de Alergia, Hospital Universitario La Paz, Madrid, Spain; ^2^Servicio de Farmacología Clínica, Hospital Universitario La Paz, Madrid, Spain; ^3^Servicio de Alergia, Hospital Universitario La Paz, Madrid, Spain; ^4^IdiPAZ, Madrid, Spain

**Correspondence:** Rosario Cabañas

*Clinical and Translational Allergy* 2016, **6(Suppl 3)**:P162

**Background:** DRESS (drug reaction with eosinophilia and systemic symptoms) is a severe cutaneous adverse reaction (cADR). Causality assessment is often a problem in polymedicated patients. The probability algorithm of the Spanish Pharmacovigilance System (Capellá and Laporte 1993) establishes different categories of causality after scoring of chronology, bibliography, drug withdrawal, re-exposure, and alternative causes (unlikely: <0, conditional: 1–3; possible 4–5, probable: 6–7, definite: >8). LTT has been used as a tool to determine the causality of cADR. LTT specificity and sensitivity are difficult to ascertain as the gold standard (re-challenge) is not acceptable in severe cases.

**Materials and methods:** A total of 34 LTT assays were performed with suspected drugs in 15 DRESS cases included in the Spanish registry PIEL*enRED*. Results were considered positive if stimulation indexes (SI) were ≥2, except for betalactam antibiotics (SI ≥ 3) or iodinated contrast media (SI ≥ 4). The Spanish Pharmacovigilance System algorithm (SPA) was applied and suspected drugs were divided into two categories: unrelated (SPA score ≤3) and related (SPA score ≥4).

**Results:** Positive LTT results were obtained in all patients. In 19 out of 23 positive LTT assays the drugs were related to the adverse reaction (SPA score ≥4). Ten out of the 11 drugs that tested negative were predicted to be unrelated to the adverse reaction (SPA score ≤3). A contingency table was built (Table 9). Fisher’s exact test was used to analyze the data. There was a significant agreement between the two methods (OR 47.5; 95 % CI 3.9–1342.94). If the pharmacovigilance algorithm was considered as gold standard, 95 % sensitivity and 71 % specificity were obtained for LTT assays, with a positive predictive value (PPV) = 82 % and negative predictive value (NPV) = 90 % (p < 0.001). On the other hand, if LTT was considered as gold standard, 82 % sensitivity and 90 % specificity was obtained for the SPA, with a PPV = 90 % and NPV = 70 % (p < 0.001).

**Table 9:** Drug causality data in DRESS patients

	Pharmacovigilance algorithm (SPA) results		Total
	Related drug (SPA ≥ 4)	Unrelated drug (SPA ≤ 3)	
Positive LTT	19	4	23
Negative LTT	1	10	11
Total	20	14	34

**Conclusions:** Although this is a small case series, the results indicate that LTT is a sensitive tool for causality assessment in DRESS. Moreover, the probability algorithm of the Spanish pharmacovigilance system can be useful to identify the culprit drug during the acute disease.

### P163 The role of interleukin-22 in β-lactam hypersensitivity

#### Andrew Sullivan^1^, Paul Whitaker^2^, Daniel Peckham^2^, B. Kevin Park^1^, Dean J. Naisbitt^1^

##### ^1^University of Liverpool, Liverpool, United Kingdom; ^2^St James’s Hospital, Leeds, United Kingdom

**Correspondence:** Andrew Sullivan

*Clinical and Translational Allergy* 2016, **6(Suppl 3)**:P163

**Background:** Antigen-specific T cells are important in the aetiology of cutaneous drug hypersensitivity. Classical T_h_1/T_h_2 phenotypes are used to classify drug hypersensitivity reactions, though these do not fully characterise the function of immune cells as the newer T_h_ subsets such as T_h_9, T_h_17 and T_h_22 have not been studied.

**Materials and methods:** The aim of this study was to use the β-lactam antibiotic piperacillin as a paradigm to fully characterise the phenotype and function of drug-specific T cells. T cells were cloned from both blood and inflamed skin of hypersensitive patients and naïve T cells from healthy donors were primed to piperacillin using dendritic cells.

**Results:** Drug-specific clones were generated from both blood (n = 570, 84 % CD4) and skin (n = 96, 83 % CD4) from patients hypersensitive to piperacillin. All clones secreted high levels of IFNγ and IL13 following drug stimulation. Interleukin-22, perforin and granzyme B were also secreted by over 50 % of clones. In contrast, IL17A secretion was not detected. Naïve T cells primed to piperacillin using autologous dendritic cells, proliferated in the presence of drug (p = 0.001, SI > 2) and had a similar pattern of cytokine secretion to clones generated from hypersensitive patients. Significant differences in chemokine receptor expression were observed between the different populations of T cell clones. CLA, CXCR6 and CCR1 expression was higher on piperacillin-specific skin-derived clones when compared to non piperacillin-specific skin-derived clones (p = 0.01). CCR2, CCR4, CXCR1 and E-cadherin were higher on skin-specific clones when compared to blood-specific clones (p = 0.01). Piperacillin-specific clones isolated from blood and skin of hypersensitive patients, as well as piperacillin-specific T cells from healthy donors migrated in the presence of chemokines specific to their respective cell surface receptors, with migration to CCR4 and CCR10 most prevalent. Finally, regulation of the cytokine secretion through modulation of nuclear receptor signalling was studied. Inhibition of the aryl hydrocarbon receptor during naïve T cell priming abrogated the drug-specific cytokine response.

**Conclusions:** Our data describe a subset of piperacillin-specific T-cells that secrete IL-22, IFNγ, perforin and granzyme B, but not IL-17, in response to antigen challenge. Taken together this suggests that IL-22 is important in the progression of β-lactam hypersensitivity.

### P164 Vancomycin-specific T cell responses and teicoplanin cross-reactivity

#### Wei Yann Haw, Marta E. Polak, Carolann Mcguire, Michael R. Ardern-Jones

##### University of Southampton, Southampton, United Kingdom

**Correspondence:** Wei Yann Haw

*Clinical and Translational Allergy* 2016, **6(Suppl 3)**:P164

**Background:** Glycopeptide antibiotics, vancomycin and teicoplanin, share a similar structure and are the mainstay of therapy for severe gram-positive organisms. Hypersensitivity responses to vancomycin are well recognised but the risk of cross-reactivity with teicoplanin is unclear. Our study aims to examine the role of T cell responses in vancomycin hypersensitivity reactions and explore the potential for cross-reactivity between vancomycin and teicoplanin.

**Materials and methods:** Our cohort comprised of vancomycin exposed allergics who had suffered delayed skin drug hypersensitivity reactions n = 17; non-allergic previously vancomycin exposed controls n = 5; and vancomycin naïve controls n = 12. We tested ex vivo drug induced lymphocyte proliferation and cytokine release. Vancomycin-specific T cell lines were grown to test for teicoplanin cross-reactivity.

**Results:** In our cohort of vancomycin allergics, vancomycin hypersensitivity reaction patterns were drug exanthems (47.1 %), DRESS (29.4 %), or SJS/TEN (23.5 %). Circulating IFN-γ vancomycin-specific T cells were identified at higher frequency than naïve controls: (IFN-γ p < 0.0001; SI p = 0.042). Interestingly, detectable frequencies in vancomycin exposed controls were higher than naïve controls (p < 0.0001; SI p = 0.12) suggesting that low frequency responses were the result of vancomycin priming even in the context of a non-allergic individual. This was confirmed by the expansion of short term T cell lines in vancomycin exposed controls (IFN-γ p = 0.008; SI p = 0.016). Cross-reactivity against teicoplanin was found to be minimal (42.7 × 10^−4^ % IFN-γ and SI 1.0 in vancomycin-specific T-cell lines compared to 48 × 10^−4^ % IFN-γ and SI 1.3 in non-vancomycin-specific T-cells lines).

**Conclusions:** Circulating vancomycin specific-T cell frequencies were found to be higher in vancomycin allergics than vancomycin exposed controls, which in turn were higher than naïve controls. Using vancomycin-specific T cell lines we did not see any evidence of teicoplanin cross-reactivity despite the similar molecular structures of the two drugs. We also showed that low-level of vancomycin-specific responses identified ex vivo in exposed controls were able to efficiently expand on short term culture, confirming that they were genuine vancomycin-specific T cells. This suggests both immune predisposition and potentially adaptive regulation may be important in regulating the development of hypersensitivity reactions to vancomycin.

**Keywords:** Drug hypersensitivity reactions; T cell; Vancomycin; In-vitro diagnostic tests

## Poster Walk 19: BAT and biomarkers (P165–P173)

### P165 A combination of early biomarkers useful for the prediction of severe ADRs

#### Yumi Aoyama^1^, Tetsuo Shiohara^2^

##### ^1^Kawasaki Hospital Kawasaki Medical School, Okayama, Japan; ^2^Kyorin University School of Medicine, Tokyo, Japan

**Correspondence:** Yumi Aoyama

*Clinical and Translational Allergy* 2016, **6(Suppl 3)**:P165

The published version of this abstract can be found at [1].

**Reference**

1. Shiohara T, Mizukawa Y, Yumi A. Monitoring the acute response in severe hypersensitivity reactions to drugs. Curr Opin Allergy Clin Immunol 2015;15(4):294–9.

### P166 Basophil activation test in the diagnostic approach of reactions during general anaesthesia

#### Ana Moreira^1^, Susana Cadinha^1^, Patrícia Barreira^1^, Ana Castro Neves^1^, Daniela Malheiro^1^, Sara Correia^2^, J. P. Moreira Da Silva^1^

##### ^1^Centro Hospitalar Vila Nova Gaia, Vila Nova Gaia, Portugal; ^2^Centro Hospitalar de Setúbal, Setúbal, Portugal

**Correspondence:** Ana Moreira

*Clinical and Translational Allergy* 2016, **6(Suppl 3)**:P166

**Background:** Neuromuscular blocking agents (NMBAs), latex and antibiotics are the most frequent agents causing hypersensitivity reactions (HR) in the perioperative setting, followed by hipnotics and opioid analgesics. The diagnostic approach is complex and relies on suggestive history and skin tests (ST), since basophil activation test (BAT) has not yet been validated and drug provocation test has limited indications because of the pharmacological effects of these drugs. Our aim was to characterize a series of subjects with suspected HR during general anaesthesia (GA) and to evaluate the concordance between ST and BAT in patients submitted to both diagnostic procedures with the suspected drugs: fentanyl (F), propofol (P) and rocuronium (R).

**Materials and methods:** Retrospective analysis of medical records from patients referred to our department, from 2009 to 2015, with suspected HR during GA. The data collected were: demographic data, atopy, clinical manifestations, results from ST and BAT. ST and BAT were performed in the following concentrations: 0.05 mg/ml for F and 10 mg/ml for P and R (SPT); 1/100 dilution for R and 1/10 for F and P (ID); 25, 12.5 and 6.25 μg/ml for F; 100, 50 and 10 μg/ml for P; 10, 5, 2.5 μg/ml for R (BAT).

**Results:** From a total of 34 patients referred to our department, we enrolled 15: mean age 44 (± 21) years; 8 female; 5 atopic; 1 had asthma and 3 had hypertension. Cutaneous reactions were reported by 7 patients, anaphylaxis by 6 and isolated respiratory symptoms by 2. BAT and ST to F were performed in 12 patients, to P in 13 and to R in 12. SPT and ID performed with F (12) and P (13) were all negative. In the case of R SPT were positive in 1 out of 12 tests and ID were positive in 2 out of 11. TAB performed with F (12) was negative in 10 cases, positive in 1 and in determined in another one. TAB performed with P (13) was negative in 9, positive in 3 and indetermined in 1. In the case of R (12) TAB was negative in 10 and positive in 3. Results to ST and BAT were concordant in 83 % of patients tested with F, 69 % with P and 67 % with R. Allergy was excluded by subsequently administration of the suspected drugs in 5 patients (1 F, 4 P). One of them had negative ST and positive BAT (P).

**Conclusions:** ST and BAT to the suspected drug were concordant in the majority of cases (83 % F, 69 % P, 67 % R). According to this results BAT seems to be a valuable contribution in the diagnostic approach of these patients.

**Keywords:** Basophil activation test; General anaesthesia

### P167 IL-10 can be related to successful desensitization

#### Asli Gelincik, Semra Demir, Fatma Sen, Hamza Ugur Bozbey, Muge Olgac, Derya Unal, Raif Coskun, Bahauddin Colakoglu, Suna Buyuozturk, Esin Çatin-Aktas, Gunnur Deniz

##### Istanbul University, Istanbul Faculty of Medicine, Istanbul, Turkey

**Correspondence:** Asli Gelincik

*Clinical and Translational Allergy* 2016, **6(Suppl 3)**:P167

**Background:** The mechanism of drug desensitization is scarcely understood. The aim of the study is to observe the cytokine levels in the serum of patients who underwent a successful drug desensitization.

**Materials and methods:** Patients with a hypersensitivity reaction to any culprit drug and therefore has to be desensitized with the drug were included into the study. IL-4, IL-5, IFN γ and IL-10 levels were determined with ELISA in the peripheral serum samples of the patients before the desensitization to any culprit drug and within 24 h after the procedure. The results were compared with the serum samples of patients who could tolerate the same drugs and healthy subjects who were not exposed to these drugs.

**Results:** 26 patients who experienced allergic reactions due to various drugs and therefore had to be desensitized, 10 patients who could tolerate the same drugs and 5 healthy subjects were included. The diagnosis of the patients were as follows: malignancy (14 patients), multiple sclerosis (2 patients), metabolic storage disorders (2 patients), iron salt deficiency (2 patients) and Basedow Graves disease (1 patient), coronary heart disease (1 patient), and congenital adrenal hyperplasia (1 patient). The drugs used for desensitization in the order of the most frequent to the least were as follows: parenteral or per oral chemotherapeutics, aspirin, corticosteroids, storage enzymes, iron salts and anti-thyroidal drugs. Skin prick tests were positive in 5 patients with parenteral chemotherapeutics, 2 patients with iron salts, 2 patients with storage enzymes and in one patient with methylprednisolone. The baseline cytokine levels were not statistically different between the three groups. The desensitization was not successful in 4 of the patients and because of this insufficient patient number their cytokine levels were not further analyzed. The IL-10 levels after the successful desensitization procedure in 22 patients significantly increased when compared to their baseline levels (p: 0.006). The rise in IL-10 levels were greater in chemotherapeutic desensitizations than the desensitizations with other drugs (p: 0.005) whereas the other three cytokines did not significantly change.

**Conclusions:** Successful desensitization can be related with increase in IL-10. In order to further elucidate the mechanism of successful desensitization, cells secreting IL-10 should be examined.

**Keywords:** IL-10; Desensitization

### P168 Immediate reactions to proton pump inhibitors: value of basophil activation test

#### Maria Salas^1^, Jose Julio Laguna^2^, Esther Barrionuevo^3^, J. Dionicio^2^, Tahia Fernandez^4^, R. Gonzalez-Mendiola^2^, I. Olazabal^5^, Maria Dolores Ruiz^1^, Miguel Blanca^1^, Cristobalina Mayorga^6^, Maria José Torres^1^

##### ^1^Regional Hospital of Málaga-IBIMA, Málaga, Spain; ^2^Hospital de la Cruz Roja, Madrid, Spain; ^3^Regional Hospital of Málaga-IBIMA, Mdálaga, Spain; ^4^Research Laboratory, IBIMA, Málaga, Spain; ^5^Inmunology Department. Alfonso X el Sabio University, Madrid, Spain; ^6^Research Laboratory-IBIMA, Málaga, Spain

**Correspondence:** Maria Salas

*Clinical and Translational Allergy* 2016, **6(Suppl 3)**:P168

**Background:** The incidence of allergic reactions to proton pump inhibitors (PPI) has increased in recent years. Most publications describe isolated cases and limited data are available about the value of skin tests. Therefore, the diagnostic approach is the drug provocation test (DPT), which is not risk free, especially for severe reactions. The aim of the study was to assess the value of the basophil activation test (BAT) for the diagnosis of immediate allergic reactions to PPI.

**Materials and methods:** We evaluated 49 patients with immediate allergic reactions to PPI. Twenty-two subjects with good tolerance to PPI were included as controls. Patients with anaphylaxis or shock were diagnosed by clinical history, once other possible causes were ruled out, and those with urticaria-angioedema or pruritus, by DPT. BAT with omeprazole and pantoprazole at 3 different concentrations (2, 0.2 and 0.02 mg/ml) using CD193 (CCR3) and CD203c for basophil selection and CD63 as activation marker, was performed in all patients and controls.

**Results:** The PPIs involved were omeprazole (N = 43), pantoprazole (N = 1) lansoprazole (N = 2) and esomeprazole (N = 2), one patient had two reactions, one with omeprazole and other one with pantoprazole. A total of 20 cases (40.81 %) reported anaphylaxis, 12 (24.48 %) anaphylactic shock, 13 (26.53 %) urticaria-angioedema, and 4 (8.16 %) pruritus. BAT was positive in 36 cases (73.46 %): 14 to omeprazole and pantoprazole (28.5 %), 19 to omeprazole (38.7 %) and 3 to pantoprazole (6 %). BAT was negative in control patients, indicating a specificity of 100 %. BAT sensitive was 82 %.

**Conclusions:** Immediate hypersensitivity reactions to PPI do occur, with omeprazole being the most frequently involved. Anaphylaxis is the most common clinical entity. BAT is a useful method for diagnosing these patients with a good sensitivity and specificity.

### P169 Improvement of the elevated tryptase criterion to discriminate IgE from non-IgE mediated allergic reactions

#### Gabriel Gastaminza, Alberto Lafuente, Carmen D’Amelio, Amalia Bernad, Olga Vega, Roselle Catherine Madamba, M. Jose Goikoetxea, Marta Ferrer, Jorge Núñez

##### Clinica Universidad de Navarra, Pamplona, Spain

**Correspondence:** Gabriel Gastaminza

*Clinical and Translational Allergy* 2016, **6(Suppl 3)**:P169

**Background:** There is no consensus on an optimal cutoff point (COP) of blood tryptase during the reaction (TDR) to discriminate IgE- from non-IgE-mediated reactions. We aimed to compare the diagnostic accuracy between TDR and TDR/basal tryptase (TDR/BT) index for discriminating IgE- from non-IgE-mediated reactions, and to estimate a COP for the best of these tests.

**Materials and methods:** Patients with an immediate allergic reaction treated in Clínica Universidad de Navarra (Spain) from 2009 to 2015. Allergological study was performed to classify the reaction into IgE- or non-IgE-mediated. The area under de curve (AUC) of the receiver operating characteristic (ROC) analysis was calculated to indicate the discriminative power of each test.

**Results:** We included 102 patients (45 % men; aged 3–99 years; 48 % with IgE-mediated reaction; 54 % with anaphylaxis criteria) who had an allergic reaction (emergency room, n = 27; hospitalization, n = 15; perioperative, n = 44; other hospital areas, n = 16).

The median TDR for the IgE-mediated reactions was 7.6 µg/l (p25–75: 4.9–13.8 µg/l) and 5.2 µg/l (p25–75: 3.6–7.5 µg/l) for the non-IgE-mediated reactions (p = 0.047).

The median TDR/BT ratio was 2.6 (p25–75: 1.7–4.3) in IgE-mediated reactions and 1.2 (p25–75: 1.0–1.6) in non-IgE-mediated reactions (p = 0.001).

The TDR/BT ratio showed the greatest ability to discriminate IgE from non-IgE meditated reactions compared with TDR (AUC TDR/BT = 0.78 and AUC TDR = 0.65; p = 0.003). The optimal COP for TDR/BT to discriminate between IgE and non-IgE reactions was 1.66.

**Conclusions:** TDR/BT ratio showed a significantly better diagnostic performance than TDR to discriminate IgE from non-IgE mediated allergic reactions. An optimal TDR/BT ratio threshold around 1.7 may be useful in clinical practice to classify allergic reactions as IgE or non-IgE mediated.

**Keywords:** Anaphylaxis; Tryptase

### P170 Low expression of Tim-3 could serve as a biomarker for control and diagnose maculopapular exanthema induced by drugs

#### Tahia Diana Fernández^1^, Inmaculada Doña^2^, Francisca Palomares^1^, Rubén Fernández^1^, Maria Salas^2^, Esther Barrionuevo^2^, Maria Isabel Sanchez^2^, Miguel Blanca^2^, Maria José Torres^2^, Cristobalina Mayorga^1^

##### ^1^Research Unit for Allergic Diseases. IBIMA-Regional University Hospital of Malaga-UMA, Málaga, Spain; ^2^Allergy Unit. IBIMA-Regional University Hospital of Malaga-UMA, Málaga, Spain

**Correspondence:** Francisca Palomares

*Clinical and Translational Allergy* 2016, **6(Suppl 3)**:P170

The published version of this abstract can be found at [1].

**Reference**

1. https://aaaai.confex.com/aaaai/2016/webprogram/Paper23785.html.

### P171 Role of basophil activation test using two different activation markers for the diagnosis of allergy to fluoroquinolones

#### Esther Barrionuevo^1^, Tahía Fernandez^2^, Arturo Ruiz^1^, Adriana Ariza^2^, Maria Salas^1^, Inmaculada Doña^1^, Ana Molina^2^, Miguel Blanca^1^, Maria Jose Torres^1^, Cristobalina Mayorga^2^

##### ^1^Regional Hospital of Málaga, Málaga, Spain; ^2^Research Laboratory-IBIMA, Málaga, Spain

**Correspondence:** Esther Barrionuevo

*Clinical and Translational Allergy* 2016, **6(Suppl 3)**:P171

**Background:** Fluoroquinolones (FQ) are the second most frequent cause of hypersensitivity to antibiotics after betalactams. Most reactions induced by FQ were immediate. For the in vitro diagnosis only the basophil activation test (BAT) has shown to be useful although with suboptimal sensitivity. The aim of our study was to analyze the BAT to FQ using two different activations markers, CD63 and CD203c, in the evaluation of patients with immediate allergic reactions to these drugs.

**Materials and methods:** Seventeen patients with confirmed immediate allergic reactions to FQ (6 to Ciprofloxacin and 11 to Moxifloxacin) were included in the study. Eighteen controls with tolerance to FQ were also included. BAT was performed with Moxifloxacin and Ciprofloxacin at 2 and 0.2 mg/ml using CD203c and CD63 as activation markers. Positive results were considered when SI > 3 to at least one of the concentrations used in the test.

**Results:** Data indicated that although both Ciprofloxacin and Moxifloxacin are able to induce both activation marker expression (CD63 and CD203c), there is a predominance in the expression of each one depending on the drug included in the test. Thus Ciprofloxacin was able to mainly increase the expression of CD63 (40 %, p = 0.0053) whereas Moxifloxacin mainly increase the expression of CD203c (10 %). In addition, analyzing the expression of both markers in basophils from Moxifloxacin allergic patients stimulated with the culprit drug, we found a higher expression of CD203c in patients suffering anaphylactic shock (7 %), whereas was CD63 the marker that showed a higher up-regulation in patients with anaphylaxis (17 %). When we analyzed the sensitivity and specificity of the test using these activation markers we can see that the best results were observed using the culprit drug and CD203c as activation marker for Moxifloxacin (S = 36.4 % and E = 94.4 %) and CD63 for Ciprofloxacin (S = 83.3 % and E = 88.9 %).

**Conclusions:** The BAT must be performed using the culprit drug and CD203c for Moxifloxacin or CD63 for Ciprofloxacin as activation marker to diagnose Quinolone Allergy. Although this differential expression of both activation markers seems to be also related with the culprit drug and clinical entity. Using this criteria and a cut-off of 3, we have obtained a better sensitivity for Ciprofloxacin.

### P172 The importance of basophil activation test in anaphylaxis due to celecoxib

#### Amalia Bernad Alonso, Carmen D’Amelio Garófalo, Olga Vega Matute, Marta Ferrer Puga, María José Goikoetxea Lapresa, Roselle Catherine Yu Madamba, Gabriel Gastaminza Lasarte

##### Clínica Universidad de Navarra, Pamplona, Spain

**Correspondence:** Amalia Bernad Alonso

*Clinical and Translational Allergy* 2016, **6(Suppl 3)**:P172

**Background:** Celecoxib, is a specific COX-2 inhibitor which is an alternative treatment for patients with intolerance to NSAIDs.

**Report:** We present two cases of anaphylaxis due to Celecoxib. The first is a 57 year old male who was taking Celecoxib as an alternative medication for sciatic pain since he had poor gastric tolerance with NSAIDs. He had a history of idiopathic recurrent urticaria. He had an episode of anaphylactic shock 30 min after taking 1 tablet of Celecoxib which prompted him to sought consult at the emergency room wherein he was administered with Epinephrine IM. On follow up after 1 month, prick test with Celecoxib revealed negative. Because the suspicion that the reaction was caused by allergy to Anisakis, a challenge test with Celecoxib was done that resulted positive, and epinephrine was administered. Basophil activation test was positive with Celecoxib and negative to Parecoxib. Two months later, he presented with hives 2 h after taking 750 mg of acetylsalicylic acid (ASA). Tolerance test with Meloxicam and Nabumetone were performed which the patient tolerated well.

The second case is a 58 year old male who had an episode of anaphylaxis 3 h after taking Celecoxib for headache. Tryptase was elevated, 19.9 μg/l. A month earlier, he had generalized itching after taking one tablet of celecoxib. He took Metamizol previously which he tolerated well. No history of atopy nor allergy to medications. Skin tests to NSAIDs were negative. Basophil activation test was positive with celecoxib and negative to ASA, Metamizole, Paracetamol, Parecoxib and Dexketoprofen. He underwent challenge test with Aspirin with positive result. Both patients were diagnosed with intolerance to NSAIDs and anaphylaxis secondary to Celecoxib.

**How this report contributes to current knowledge:** Anaphylaxis due to Celecoxib is considered a rare entity as patients with intolerance to NSAIDs usually tolerate Celecoxib and other COX-2 inhibitors. The positive result in the basophil activation test together with a compatible clinical history makes us hypothesize that these are cases of a selective hypersensitivity to Celecoxib, instead of the fact that both patients were finally NSAIDs intolerant. Hence, BAT can be useful for the diagnosis, given the low sensitivity of skin tests.

**Consent:** Written informed consent was obtained from the patient for publication of this abstract and any accompanying images.

### P173 The role of basophil activation test in the diagnosis of immediate type drug hypersensitivity to betalactam antibiotics

#### Antonia Thinnes, Hans F. Merk, Jens Malte Baron, Martin Leverkus, Galina Balakirski

##### Department for Dermatology and Allergology, University Hospital of Aachen, Aachen, Germany

**Correspondence:** Galina Balakirski

*Clinical and Translational Allergy* 2016, **6(Suppl 3)**:P173

**Background:** Basophil activation test (BAT) is reported to be a useful and very promising technique in the diagnosis of immediate type drug hypersensitivity reactions. It is used in combination with in vivo and in vitro diagnostic tools and may contribute to the sensitivity of the diagnostic work out.

**Materials and methods:** In order to investigate the role of BAT in the diagnosis of immediate type drug hypersensitivity to betalactam antibiotics we analyzed all BATs performed with betalactam antibiotics in our department during the period from 2009 to 2012. We compared the results of in vivo diagnostics (skin prick test, intracutaneous test, patch test) and in vitro diagnostics (specific IgE) with the results of the BAT under the aspect, if BAT represent a useful tool for assessment of the individual risk of the patient to experience another immediate type drug hypersensitivity reaction on reexposure to the tested drug.

**Results:** We performed BAT with betalactam antibiotics in 64 cases: 20 % (n = 13) with penicillin (PEN), 38 % (n = 24) with aminopenicilins (AMP) and 42 % (n = 27) with cephalosporins (CPH).

In the PEN-group 23 % (n = 3) of the patients had at least one positive in vivo test, but negative BAT and 15 % (n = 2) had positive BAT, but negative in vivo tests.

In the AMP-group 17 % (n = 4) of patients had at least one positive in vivo test, but negative BAT and 17 % (n = 4) had positive BAT, but negative in vivo tests. Only 8 % (n = 2) of patients had positive both BAT und at least one in vivo test. 4 % (n = 1) of patients had both positive at least one in vivo test and specific IgE, but negative BAT.

In the CPH-group 22 % (n = 6) of the patients had positive BAT, but no other positive test results and 4 % (n = 1) had positive both BAT und at least one in vivo test.

**Conclusions:** In case of the negative in vivo and in vitro test results (inclusive BAT) the individual risk of the patient to experience another immediate type drug hypersensitivity reaction on reexposure to the tested drug was considered to be low, so drug provocation test (DPT) as the next diagnostic step was recommended: from overall of 41 such cases DPT was performed in 32 % (n = 13) and was unremarkable in 100 % (n = 13).

Our results confirm that BAT may be an important tool to increase the sensitivity of the diagnostic and make the better risk assessment possible. However, the limitation of this study is that we didn’t perform DPT in patients with positive in vivo or in vitro results and therefore are not able to estimate the frequency of false positive BATs.

Frequency of positive in vivo and in vitro results in the diagnostic work out of immediate type drug hypersensitivity reactions to betalactam antibiotics in Department of Dermatology and Allergology at the University Hospital of Aachen, Germany during the period from 2009 to 2012.

Positive BAT results in patients with negative in vivo tests and specific IgE may provide important information for assessment of the individual risk of the patient to experience another immediate type drug hypersensitivity reaction on reexposure to the tested drug (Table 10).

**Table 10:** The role of basophil activation test in the diagnosis of immediate type drug hypersensitivity to betalactam antibiotics

	Penicillin 20 % (n = 13)	Aminopenicillins 38 % (n = 24)	Cephalosporins 42 % (n = 27)	Total 100 %(n = 64)
positive in vivo results and negative BAT	23 % (n = 3)	17 % (n = 4)	–	11 % (n = 7)
negative in vivo results as well as absence of specific IgE and positive BAT	*15* *% (n* = *2)*	*17* *% (n* = *4)*	*22* *% (n* = *6)*	*19* *% (n* = *12)*
positive in vivo results and positive BAT	–	8 % (n = 2)	4 % (n = 1)	4.5 % (n = 3)
positive in vivo results, positive specific IgE and negative BAT	–	4 % (n = 1)	–	1.5 % (n = 1)
negative in vivo results and negative BAT	62 % (n = 8)	54 % (n = 13)	74 % (n = 20)	64 % (n = 41)

**Keywords:** Basophil activation test; Betalactam antibiotics; Immediate type drug hypersensitivity

## Poster Walk 20: TCR recognition, cellular (P174–P183)

### P174 Characterisation of the effect of co-inhibitory signalling on the activation of drug-derived antigen-specific T-cells

#### Andrew Gibson, Monday Ogese, Lee Faulkner, B. Kevin Park, Dean J. Naisbitt

##### University of Liverpool, Liverpool, United Kingdom

**Correspondence:** Andrew Gibson

*Clinical and Translational Allergy* 2016, **6(Suppl 3)**:P174

**Background:** The factors governing inter-individual susceptibility to drug hypersensitivity remain ill-defined. Although the association of specific HLA alleles with hypersensitivity is important, for most drugs, the majority of individuals who are positive for an HLA risk allele do not develop a reaction. Thus, predisposition is likely mediated by other parameters, which may include T-cell co-inhibitory pathways. As polymorphisms in co-inhibitory pathways are associated with dysregulated immune responses, we investigated the role of these pathways during drug (SMX-NO)-specific T-cell responses. Programmed death-1 (PD-1) and cytotoxic T-lymphocyte associated protein 4 (CTLA4) are considered to be key immune checkpoints, and TIM-3 is of current interest due to its reported upregulation alongside PD-1.

**Materials and methods:** Naïve and memory T-cells from healthy donors were incubated for 8 days with SMX-NO and dendritic cells ± PD-L1, CTLA4, TIM-3 blocking antibody. Antigen-reactivity was then assessed by T-cell cytokine secretion and proliferation. Cell phenotype was assessed by flow cytometry. T-cell clones were then generated from these cultures.

**Results:** While blockade of PD-L1 or CTLA4 enhanced the activation of SMX-NO-primed naïve T-cells, only the blockade of CTLA4 enhanced the proliferative response of antigen-stimulated memory T-cells suggesting a greater regulatory role for CTLA4 during secondary T-cell responses. Blockade of TIM-3 had no effect on T-cell activation of either naïve or memory cells. While all receptors were upregulated on T-cells after antigen exposure, PD-1 was upregulated at earlier time points than CTLA4 and TIM-3 indicating a differential role for these receptors during early and late stage T-cell activation. High expression of individual co-inhibitory receptors has previously been associated with exhausted T-cells, while other studies indicate that these cells are highly functional. We found no correlation between the level of individual co-inhibitory receptor expression and the strength of T-cell activation in the T-cell clones.

**Conclusions:** Drug-induced stimulation of naïve T-cells was significantly enhanced by blocking PD-L1 or CTLA4. However, the co-inhibitory pathways did not have a great effect on memory T-cell responses. Any differences in the control of the different T-cell co-inhibitory pathways may be important in determining whether a particular individual develops a hypersensitivity response to a drug.

### P175 Characterization of drug hapten-specific T cell responses in piperacillin hypersensitive patients

#### Zaid Al-Attar^1^, Fiazia Yaseen^1^, Xiaoli Meng^1^, Rozalind Jenkins^1^, Paul Whitaker^2^, Daniel Peckham^2^, Lee Faulkner^1^, John Farrel^1^, Kevin Park^1^, Dean Naisbitt^1^

##### ^1^MRC Centre for Drug Safety Science, Dept Molecular & Clinical Pharmacology, University of Liverpool, Liverpool, United Kingdom; ^2^Regional Adult Cystic Fibrosis Unit, St James’s Hospital, Leeds, United Kingdom

**Correspondence:** Zaid Al-Attar

*Clinical and Translational Allergy* 2016, **6(Suppl 3)**:P175

**Background:** Piperacillin is a b-lactam frequently used to combat bacterial infections in patients with cystic fibrosis. However, its use is associated with high incidence of delayed-type hypersensitivity reactions. Previous studies have identified lymphocyte proliferative responses and cytokine secretion from PBMCs isolated from approximately 75 % of hypersensitive patients but not from tolerant controls. The drug forms a hapten that binds covalently to specific lysine residues on serum albumin (HSA).

**Aim**: To generate a synthetic piperacillin-HSA conjugate and to quantify the level of modification at specific lysine residues. Furthermore, the activation of T-cells with free drug and the drug-protein conjugate was assessed.

**Materials and methods:** Piperacillin was incubated with HSA at different molar ratios (10:1–250:1 drug:protein). Mass spectrometry was used to characterize modification at specific lysine residues. PBMCs from hypersensitive patients were cultured in the presence of parent drug and the piperacillin-HSA conjugate and T-cell clones generated by serial dilution. T cell clones were analysed for antigen-specific proliferative responses, cytokine release and TCR-Vb usage.

**Results:** Cyclised and hydrolysed forms of piperacillin hapten were detected on over 10 lysine residues of HSA. Quantitative analysis revealed that 3–23 % of lysine 541 was modified with piperacillin haptens. Sixty CD4+ clones displayed reactivity against piperacillin-modified HSA but did not respond to free piperacillin or to other b-lactam HSA adducts. T cell activation was dependent on protein processing by antigen presenting cells and the level of modification with the piperacillin hapten, as low levels of modification failed to activate the clones. A further sixty CD4+ clones were responsive to free piperacillin and were not activated with the piperacillin-HSA conjugates. T-cell clones secreted a variety of molecules: IFN-γ (72 % of clones), IL5 (48 %), IL13 (37 %), perforin (48 %), granzyme B (26 %) and FasL (52 %) and showed a restricted expression of TCR-Vb9 (68 %).

**Conclusions:** We have shown that drug hapten-responsive CD4+ T-cells with divergent antigen specificities circulate in hypersensitive patients. These data have important implications for studies investigating the nature of the drug antigen. It is necessary to synthesize conjugates with different drug haptens to fully investigate the antigen specificity of the T-cell repertoire.

### P176 Characterization of the response of T-cells to telaprevir and its metabolite in normal volunteers

#### Zaid Al-Attar, Khetam Alhilali, Yanni Xue, John Farrell, Lee Faulkner, Kevin Park, Dean Naisbitt

##### MRC Centre for Drug Safety Science, Department of Molecular and Clinical Pharmacology, University of Liverpool, Liverpool, United Kingdom

**Correspondence:** Zaid Al-Attar

*Clinical and Translational Allergy* 2016, **6(Suppl 3)**:P176

**Background:** Telaprevir is antiretroviral drug developed for the treatment of hepatitis C. Human exposure is associated with a high frequency of cutaneous side effects varying in severity from mild rash (56 %) to Stevens Johnson syndrome/toxic epidermal necrolysis (<1 %). Telaprevir interconverts to an R-diastereomer (VRT-127394), which is the major metabolite. There is no known HLA risk allele associated telaprevir hypersensitivity. However, this does not rule out a role for antigen-specific T-cells in mediating hypersensitivity.

**Aim:** The aims of this study were to (1) generate T-cell clones with specificity for telaprevir and/or its metabolite and (2) explore the nature of the induced response.

**Materials and methods:** The optimal T-cell priming concentration of telaprevir and its metabolite was identified using a PBMC toxicity assay. Naïve T-cells from 3 normal volunteers were primed with autologous dendritic cells (DCs) and telaprevir or VRT-127394 for 10 days. Alternatively, PBMCs from 3 normal volunteers were cultured with telaprevir or VRT-127394 for 14 days. T-cells from both experimental procedures were cloned by serial dilution and repetitive mitogen stimulation. T-cell proliferative responses were assessed using [^3^H] thymidine incorporation and IFN-γ. Antigen-specific clones were phenotyped for CD4, CD8 and TCR Vb expression.

**Results:** T-cell responses to telaprevir or VRT-127394 were not detected using the DC priming assay. However, 13 telaprevir-responsive T-cell clones were isolated from PBMC cultures from one volunteer. All 13 clones were stimulated to proliferate in the presence of telaprevir and its metabolite in a dose-dependent manner at a similar concentration range. Clones were not activated in the presence of antigen presenting cells pulsed with telaprevir for 1–16 h. 11 clones secreted IFN-γ when activated with telaprevir. 10 clones were CD4+ and 3 were CD8+. Clones expressed a restricted pattern of TCR-Vb which was dominated by Vb2 and Vb22.

**Conclusions:** The data presented here shows that telaprevir-responsive T-cells were detected in healthy volunteers. CD4+ and CD8+ clones were activated with both telaprevir and the major metabolite VRT-127394. Future work will focus on assessing the nature of the T-cell response in hypersensitive patients.

### P177 Characterization of the T cell receptor signatures of drug-responsive T cells

#### Patricia Illing^1^, Nicole Mifsud^1^, Heidi Fettke^1^, Jeffrey Lai^1^, Rebecca Ho^1^, Patrick Kwan^2^, Anthony Purcell^1^

##### ^1^Infection and Immunity Program, Monash Biomedicine Discovery Institute and Department of Biochemistry and Molecular Biology, Monash University, Clayton, Australia; ^2^Royal Melbourne Hospital, University of Melbourne, Parkville, Australia

**Correspondence:** Patricia Illing

*Clinical and Translational Allergy* 2016, **6(Suppl 3)**:P177

**Background:** The strongest associations reported to date between adverse drug reactions and specific Human Leukocyte Antigen (HLA) allotypes are between HLA-B*57:01 and abacavir hypersensitivity syndrome, HLA-B*58:01 and allopurinol hypersensitivities, and HLA-B*15:02 and carbamazepine induced Stevens–Johnson Syndrome/Toxic Epidermal Necrolysis. A lesser association also exists between HLA-A*31:01 and carbamazepine induced hypersensitivities. In this study we examined the diversity of the T cell response to these drugs (and metabolites) by characterising the T cell receptor (TCR) signatures of drug-responsive CD8+ T cells at a single cell level in patients and healthy donors. These studies were complimented by analyses of the diversity and perturbation of the HLA peptide repertoire following drug/metabolite exposure.

**Materials and methods:** To characterise drug responsive TCR signatures, peripheral blood mononuclear cells were stimulated with the parent drug or metabolite in vitro and drug-responsive T cells characterized (phenotype and function) by flow cytometry. Single-cell flow cytometric sorting of drug-responsive CD8+ T cells was performed prior to a paired analysis of TCR alpha and beta chain variable regions using a novel multiplex nested RT-PCR methodology. To assess the influence of drugs/metabolites on peptide presentation by HLA molecules, HLA transfected B-Lymphoblastoid cell lines were grown to high density in the presence of the molecule of interest. 5–10 × 10^8^ cells were harvested, the HLA molecules immunoaffinity purified, and the peptide/small molecule ligands dissociated and analysed using Liquid Chromatography Tandem Mass Spectrometry.

**Results:** Our data demonstrated that the TCR signatures for abacavir-responsive CD8+ T cells were oligoclonal, correlating with previous reports of a large and diverse perturbation in HLA-B*57:01 peptide repertoire during abacavir exposure. Interestingly, whilst both carbamazepine and allopurinol hypersensitivities are postulated to occur via a direct interaction of the HLA-peptide-drug-TCR at the cell surface, and cause minimal change of the HLA peptide repertoire, their TCR usage profiles are divergent with carbamazepine being more restricted and allopurinol/oxypurinol exhibiting greater diversity.

**Conclusions:** These data suggest a spectrum of diversity in the T cell response that may correlate with altered modes of drug presentation in HLA associated drug hypersensitivity.

**Keywords:** Human leukocyte antigen; Carbamazepine; Allopurinol; Abacavir

### P178 Defining the signals between hepatocytes and immune cells in idiosyncratic drug-induced liver injury (DILI)

#### Monday O. Ogese^1^, Lee Faulkner^2^, B. Kevin Park^2^, Catherine Betts^1^, Dean J. Naisbitt^2^

##### ^1^AstraZeneca R&D, Cambridge, United Kingdom; ^2^University of Liverpool, Liverpool, United Kingdom

**Correspondence:** Monday O. Ogese

*Clinical and Translational Allergy* 2016, **6(Suppl 3)**:P178

**Background:** An association between HLA genotype and the development of certain forms of DILI is well established. Furthermore, it is now apparent that drug-specific T-cells are activated in certain patients with DILI. The cross-talk signals between hepatocytes and the immune cells resident in the liver and circulation are likely to be critical in determining the outcome of drug exposure and development of immune-mediated DILI. However, tissue-specific immune signalling with respect to human DILI remains largely unexplored. Thus, the aim of this study was to profile the signals released by fresh human hepatocytes upon drug exposure and to characterise the impact of these molecules (cytokines and chemokines) on the phenotype and function of antigen presenting cells (APC).

**Materials and methods:** Fresh human hepatocytes were exposed to three test compounds implicated in DILI (flucloxacillin, amoxicillin and isoniazid) and one reactive metabolite, nitroso-sulphamethoxazole (SMX-NO), and end-points of hepatocyte toxicity, cell viability and oxidative stress assessed.

**Results:** HMGB1 and LDH release as well as ATP depletion occurred in a drug- and concentration-dependent manner. Furthermore, compound-specific activation of Nrf2 marker genes was observed with each of the test drugs. SMX-NO differentially induced the expression of NQO1, TXNRD1 and SRXN1 responsible for cytoprotection against chemically reactive metabolites. Also, the expression of AKR1B10, SRXN1 and LOC344887 were significantly increased by all the test compounds. Hepatocytes cultured with test drugs released a mixture of pro-inflammatory (IFN-γ, GM-CSF, IL-12p40, IL-12p70, IL-1β, TNF-α, TNF-β, IL-8 and MCP-1) and anti-inflammatory cytokines (IL-1RA, IL-10 and IL-13). Co-culture of APC with supernatant from drug treated hepatocytes resulted in a highly drug-dependent release of cytokines as well as significant changes in expression of MHC class II and CD86 on the cell surface of dendritic cells.

**Conclusions:** In conclusion, our study begins to define the various factors that might be important in determining whether drug exposure in patients results in an immune response and tissue injury. We found that cross-talk signals were highly drug specific. The development of a liver/immune cell culture system will be an important step forward in advancing our understanding of the molecular mechanisms underlining the development of idiosyncratic DILI.

**Keywords:** DILI; Hepatocytes; Idiosyncratic; Cytokines

### P179 Development of novel chemicals that do not bind to HLA-B*57:01 or activate CD8+ T-cells through modification of the 6-amino cyclopropyl group of abacavir

#### Paul Thomson, John Farrell, Mohammad Alhaidari, Neill Berry, Paul M. O’Neill, B. Kevin Park, Dean J. Naisbitt

##### University of Liverpool, Liverpool, United Kingdom

**Correspondence:** Paul Thomson

*Clinical and Translational Allergy* 2016, **6(Suppl 3)**:P179

**Background:** Exposure to the reverse transcriptase inhibitor abacavir has been associated with hypersensitivity reactions mediated by CD8^+^ T-cells in individuals carrying the human leukocyte antigen (HLA-B*57:01) risk allele. It has been shown that abacavir can interact directly with the antigen binding cleft of HLA-B*57:01, altering its conformation and thereby causing an alteration of the peptides displayed on the cell surface. It has been hypothesized that these alternative self-peptide sequences trigger the CD8+ T-cell response in abacavir hypersensitive patients.

We examined whether it is possible to synthesize compounds that retain antiviral activity, but do not bind to HLA-B*57:01 and activate CD8+ T-cells.

**Materials and methods:** Twenty-five abacavir analogues were synthesized with modifications around the cyclopropyl group with compounds divided into three main groups. Abacavir responsive CD8^+^ T-cell clones were generated from healthy donors positive for the HLA-B*57:01 risk allele. IFN-g secretion was measured when the clones were cultured in the presence of autologous antigen presenting cells and abacavir or abacavir analogues using an ELISpot assay. Anti-viral activity of the analogues was assessed using a range of established assays. In silico docking studies were carried out to find potential binding orientations of the abacavir analogues within the F-pocket of HLA-B*57:01.

**Results:** CD8^+^ T-cell clones proliferated and secreted IFN-g in response to abacavir. Several analogues in groups 1 and 2 displayed some antiviral activity without triggering CD8+ T-cell responses. Molecular docking studies of these analogues to HLA-B*57:01 demonstrated a quantitative relationship between the protein binding and the T-cell response. These data prompted us to synthesize a 3rd group of compounds where the NH-cyclopropyl group of abacavir was replaced with substituted cyclic amine derivatives. This third series was subjected to additional antiviral activity and T-cell response assays and several molecules were shown to be devoid of T-cell activity, whilst maintaining a favourable antiviral profile.

**Conclusions:** These studies demonstrate that modification of the cyclopropyl moiety of abacavir may result in compounds that retain the antiviral activity, without generating an unwanted T-cell response. This approach represents an exciting approach to the design of safe antiviral drugs eliminating the need for personalised medicine therapy regimes.

### P180 Generation and characterization of dapsone- and nitroso-dapsone-specific T-cell clones using lymphocytes from healthy volunteers

#### Abdulaziz Alzahrani^1^, Monday O. Ogese^2^, John Farrell^1^, Lee Faulkner^1^, Andrew Gibson^1^, Arun Tailor^1^, B. Kevin Park^1^, Dean J. Naisbitt^1^

##### ^1^University of Liverpool, Liverpool, United Kingdom; ^2^AstraZeneca R&D, Cambridge, United Kingdom

**Correspondence:** Abdulaziz Alzahrani

*Clinical and Translational Allergy* 2016, **6(Suppl 3)**:P180

**Background:** Dapsone is an antibiotic commonly used in the treatment of leprosy. Its use is associated with development of hypersensitivity in 0.5–3.6 % patients. HLA-B*13:01 is associated with an increased susceptibility to dapsone hypersensitivity but no studies have been carried out to determine the immunological mechanism(s) underlying the reactions and/or the role of the HLA risk allele in antigen presentation. The aims of this study were to: (1) Prime naive T-cells to dapsone and nitroso-dapsone (2) Generate dapsone-and nitroso-dapsone-specific T-cell clones from healthy donors and (3) Characterize the pathways of drug-specific T-cell activation.

**Materials and methods:** Peripheral blood mononuclear cells (PBMCs) were isolated from healthy volunteers, and CD14^+^ monocytes and naïve T-cells were isolated by magnetic bead separation. Monocyte-derived dendritic cells (DC) were used to prime naïve T-cells in the presence of either dapsone or nitroso dapsone using an established DC priming assay. Drug-specific T-cell clones were generated by serial dilution and repetitive mitogen stimulation. Antigen specificity was assessed by measurement of proliferation and cytokine release using [^3^H]-thymidine release and IFN-γ Elispot, respectively.

**Results:** Moderate priming of naive T-cells to both dapsone-and nitroso-dapsone was detected but only five dapsone-specific T-cell clones were generated from one out of three donors tested. No nitroso-dapsone-specific T-cell clones were detected in any of the three donors. The dapsone-responsive clones were stimulated to proliferate is a dose-dependent manner, but showed no cross reactivity with nitroso-dapsone or three closely related structures. The clones secreted IFNgamma, IL-5 and IL-13 alongside the cytolytic molecule granzyme B. All of the dapsone-specific clones were CD4^+^, and showed MHC class II-restricted activation. Use of fixed or pulsed antigen presenting cells suggested that activation of T cell clones by dapsone did not require antigen processing.

**Conclusions:** Naïve T cells were primed to both dapsone and nitroso dapsone. Dapsone-specific T-cell clones were activated via a processing independent pathway, thus suggesting a non-covalent interaction between drug, MHC and T cell receptor. On-going studies using PBMCs from volunteers ezxpressing HLA-B*13:01 will investigate the role of HLA*B13:01 in the activation of dapsone- and nitroso-dapsone-specific T cells.

**Keywords:** Dapsone; Hypersensitivity; T-cells; Dendritic cells; Cytokines

### P181 Identification of benzylpenicillin-hapten peptides responsible for naïve T-cell activation and immunization of allergic patients to penicillin

#### Marie Eliane Azoury^1^, Lucia Fili^2^, Rami Bechara^1^, Noémie Scornet^3^, Cathy Nhim^1^, Richard Weaver^4^, Nancy Claude^4^, Delphine Joseph^3^, Bernard Maillere^5^, Paola Parronchi^2^, Marc Pallardy^1^

##### ^1^University Paris-Saclay, Univ Paris-Sud, INSERM UMR996, Châtenay-Malabry, France; ^2^University of Florence, Department of Experimental and Clinical medicine, Florence, Italy; ^3^University Paris-Saclay, Univ Paris-Sud, UMR CNRS8076, Châtenay-Malabry, France; ^4^Institut de Recherches Internationales Servier, Suresnes, France; ^5^SIMOPRO, IBiTecS, CEA, Saclay, France

**Correspondence:** Marc Pallardy

*Clinical and Translational Allergy* 2016, **6(Suppl 3)**:P181

**Background:** Allergic reactions to drugs are often unpredictable and may have many side effects including anaphylaxis. According to the hapten hypothesis, drug of low molecular weight can bind to proteins and form immunogenic complexes. Antigen presenting cells, such as dendritic cells (DCs), recognize and internalize drug hapten complexes, and digest them into benzylpenicillin-hapten peptides (BP-P), which are presented on HLA molecules to drug-specific T-cells. This lead to the immunization of the exposed person. There is in vitro evidence that T-cells from allergic patients react to benzylpenicillin-Human Serum Albumin (BP-HSA) bioconjugate and our group has recently shown the existence of naïve CD4^+^ T lymphocytes specific to BP-HSA in healthy donors. In this context, we were interested in identifying BP-P from HSA able to immunize patients to BP thus contributing to hypersensitivity reactions.

**Materials and methods:** We have considered HSA as a good model for BP haptenization since BP is known to bind covalently to HSA and because HSA is the most abundant protein in the sera. We have synthetized BP-HSA bioconjugate, investigated BP-HSA-specific naïve CD4^+^ T-cell responses in healthy donors and identified BP binding positions on HSA using mass-spectrometry. Twelve 15-mer BP-P were identified as potential T-cell epitopes using the predictive IEDB computational approach and were synthesized using an original BP-lysine monomer. Naïve CD4^+^ T cells from non-allergic donors were stimulated once a week with autologous DCs loaded with BP-HSA or BP-P to amplify BP-HSA- or BP-P-specific T-cells respectively. Activation of specific CD4^+^ T-cells was detected using interferon-γ ELISpot and their frequency was calculated using the Poisson distribution.

**Results:** In this study, BP-HSA- and BP-P-specific naïve CD4^+^ T cells were detected in 15/16 and 11/14 of the tested healthy donors respectively. Most donors responded to 3 peptides with BP covalently bound on lysines 159, 212 and 525 respectively. Two of these benzylpenicillinoylated peptides (lysines 159 and 525) were found to induce peripheral blood mononuclear cells (PBMC) proliferation in patients with allergic reaction to BP using the lymphocyte transformation test.

**Conclusions:** This study showed the capacity of BP-HSA to be recognized by naïve T-cells from multiple healthy donors and allowed the identification for the first time of BP-P responsible for naïve T-cell activation and immunization of allergic patients to BP.

### P182 Massive expansion of clonotypic and polycytotoxic CD8+ T cells in toxic epidermal necrolysis

#### Axel Patrice Villani^1^, Aurore Rozières^2^, Benoît Bensaïd^3^, Mathilde Tardieu^3^, Floriane Albert^3^, Virginie Mutez^3^, Tugba Baysal^3^, Marc Pallardy^4^, Janet Maryanski^5^, Jean-François Nicolas^6^, Osami Kanagawa^3^, Marc Vocanson^3^

##### ^1^Inserm U1111 - CIRI, Lyon I University, Edouard Herriot Hospital, Lyon, France; ^2^Inserm U1111 - CIRI, Lyon I University, Lyon, France; ^3^Inserm U1111 - CIRI, Lyon, France; ^4^INSERM UMR 996, Université Paris-Sud, Châtenay-Malabry, Lyon, France; ^5^Unité de Thérapie Cellulaire et Génique (UTCG) and URE 004 (ImCelVir), Université Nice Sophia Antipolis, Nice, France; ^6^Inserm U1111 - CIRI, Lyon I University, Lyon-Sud Hospital, Lyon, France

**Correspondence:** Axel Patrice Villani

*Clinical and Translational Allergy* 2016, **6(Suppl 3)**:P182

**Background:** Toxic epidermal necrolysis (TEN) is a life-threatening and blistering adverse drug reaction, characterized by an acute epidermal necrolysis. Diverse studies have reported that the onset of TEN correlates with skin infiltration by cytotoxic lymphocytes (T, NK cells) and inflammatory monocytes.

**Materials and methods:** To further characterize the phenotype of skin-infiltrating lymphocytes at the acute phase of TEN, we conducted a prospective study on the blood and blister fluids from 11 TEN patients, using flow and mass cytometry, as well as next generation TCR sequencing.

**Results:** Our results confirm that conventional CD8+ T cells (CD45+ TCRab+ CD8b+) and, at a lesser extend CD4+ T cells, were the main leucocyte subsets found in recent TEN blisters. Consequently, the CD4/CD8 ratio was inversed in blisters (mean: 0.8) compared to blood (mean: 2). However, we failed to repeatedly detect NK (CD45+ TCRab+ NKP46+) or NKT cells (CD45+ TCRab+ TCRVa24+) in TEN blisters. Strikingly, deep sequencing of the T cell receptor CDR3 repertoire revealed massive clonal expansions of T cells in blister cells of 6 TEN patients, which were confirmed at TCR-Vb usage level by flow cytometry. Over-represented TCR-Vb+ blister cells were mainly effector memory CD8+ CD45RA-CD27+ T cells and displayed a poly-cytotoxic phenotype since they co-expressed Granulysin, Granzyme B, Granzyme A, Perforin and TWEAK, as demonstrated by mass cytometry.

**Conclusions:** Our results highlight a massive skin recruitment of clonotypic and poly-cytotoxic CD8+ T cells in TEN patients, which could explain the severity of this life-threatening disease.

**Keywords:** Toxic epidermal necrolysis; CD^8+^ T cells; Polycytotoxicity; Clonotypic

### P183 Pharmaco-immunological synapse of HLA-drug-TCR in SCAR

#### Shuen-Iu Hung

##### National Yang-Ming University, Taipei, Taiwan

**Correspondence:** Shuen-Iu Hung

*Clinical and Translational Allergy* 2016, **6(Suppl 3)**:P183

**Background:** Life-threatening severe cutaneous adverse reactions (SCAR), including drug reaction with eosinophilia and systemic symptoms (DRESS), Stevens–Johnson syndrome (SJS) and toxic epidermal necrolysis (TEN) are known as T cells-mediated drug hypersensitivity, some of which showed strong HLA genetic predisposition. Our previous studies have discovered that *HLA*-B*15:02, *HLA*-A*31:01, and *HLA*-B*58:01, are the genetic markers of carbamazepine-induced SJS/TEN, -DRESS, and allopurinol-SCAR, respectively. Although we recently also have identified that HLA can directly interact with the drugs and metabolites (JACI 2012; JACI 2015), the role of TCR and immune synapse of HLA-drug-TCR in SCAR remain unclear.

**Materials and methods:** We enrolled patients with SCAR caused by carbamazepine, allopurinol, phenytoin, lamotrigine or antibiotics, and collected the blister cells from skin lesions or PBMC from patients in the acute or recovery stage. We applied the technology of next-generation sequencing to investigate the TCR repertoire of SCAR. Real-time quantitative PCR was used to measure and validate the gene expression of TCR. We generated the recombinant TCRalpha/beta fusion protein, TCRalpha/beta transfectants, and used in vitro cell cultures to examine the molecular interaction of HLA-drug-TCR in drug hypersensitivity.

**Results:** We found that different drugs-induced SCAR showed various profiles of TCR usage. Particularly, limited and shared drug-specific CDR3 clonotypes were predominantly expressed in the blister cells and PBMC of SCAR patients. The in vitro drug-expanded T cells highly expressed the specific CDR3 clonotypes and produced granulysin upon drug stimulation. Furthermore, we generated drug-specific TCRalpha/beta recombinant protein, and found it could directly interact with the drug antigen and show cross-reactivity to the structure-related compounds or metabolites. Using in vitro cell culture and ELISPOT assays, we found that the specific TCRalpha/beta transfectants were activated upon drug stimulation, and the response was facilitated by the co-culture of antigen-presenting cells with the expression of matched HLA transgene.

**Conclusions:** Our data suggest that clonotype-specific TCRalpha/beta directly interacts with drug antigens presented by HLA in the T cells-mediated drug hypersensitivity. This study provides a new insight into the pharmaco-immunological synapse of HLA-drug-TCR in SCAR.

**Keywords:** Immune synapse; TCR; HLA; Drug antigen; SCAR

## Poster Walk 21: New in vitro methods, haptens, etc. (P184–P194)

### P184 Amoxicillin-clavulanate forms distinct multiple haptenic structures on human serum albumin in patients

#### Xiaoli Meng^1^, Arun Tailor^1^, Caroline J. Harrison^1^, Rosalind E. Jenkins^1^, Paul Whitaker^2^, Neil S. French^1^, Dean J. Naisbitt^1^, B. Kevin Park^1^

##### ^1^University of Liverpool, Liverpool, United Kingdom; ^2^St James’s Hospital, Leeds, United Kingdom

**Correspondence:** Xiaoli Meng

*Clinical and Translational Allergy* 2016, **6(Suppl 3)**:P184

**Background:** Amoxicillin-clavulanate (AC) is one of the most common causes of drug induced liver injury (DILI) in Europe and the US. The mechanisms of amoxicillin-clavulanate–induced liver injury (AC-DILI) remain to be defined, however, recent studies have shown that AC-DILI is associated with both HLA class I and class II alleles, indicating immune-mediated mechanisms have been involved.

**Materials and methods:** In order to investigate the molecular basis involved in AC-DILI, we have characterized the binding of AC to proteins in vitro and in patients receiving amoxicillin-clavulanate therapy using novel mass spectrometric methods. Clavulanic acid was incubated with N-acetyl lysine in phosphate buffer and the structures of adducts were characterised by mass spectrometry. In addition, amoxicillin and clavulanic acid were incubated with human serum albumin (HSA) in vitro to identify possible adducts formed on proteins, and the protein adducts formed in cell culture medium and in patients were also characterised by mass spectrometric methods.

**Results:** Amoxicillin formed adducts with lysine residues on HSA in vitro, with K190, K199 and K525 being the most reactive sites. In addition, amoxicillin-modified K190 and K525, and novel adducts derived from amoxicillin dimmer were also detected in plasma samples from patients, and more extensive modification was observed in patients that had been administered higher doses of amoxicillin. The binding of clavulanic acid to HSA was rather complicated compared to amoxicillin. A total of **seven** types of adducts were identified when clavulanic acid was incubated with HSA at high concentration in vitro, including that formed by direct binding of clavulanic acid to lysine residues, novel pyrazine adducts derived from binding to the degradation products of clavulanic acid, and a cross-linking adduct. Importantly, stable adducts derived from formylacetic acid and pyrazine were also detected in all patients.

**Conclusions:** The finding of distinct novel types of AC haptens formed in patient is vital for further exploration of the immunological consequences to define the mechanisms of drug hypersensitivity exemplified by AC.

**Keywords:** Covalent binding; Haptenic structures; Albumin

### P185 Dendrimeric antigens for studying the influence of penicillin determinants orientation on IgE recognition

#### Maria Isabel Montañez^1^, Cristobalina Mayorga^2^, Francisco Najera^3^, Adriana Ariza^1^, Tahia D. Fernandez^1^, Maria Salas^2^, Angela Martin-Serrano^1^, Miguel Blanca^2^, Ezequiel Perez-Inestrosa^3^, Maria Jose Torres^2^

##### ^1^Research Laboratory. IBIMA, Regional University Hospital of Malaga, UMA, Malaga, Spain; ^2^Allergy Unit. IBIMA, Regional University Hospital of Malaga, UMA, Malaga, Spain; ^3^Department of Organic Chemistry, University of Malaga, IBIMA, BIONAND, Malaga, Spain

**Correspondence:** Maria Isabel Montañez

*Clinical and Translational Allergy* 2016, **6(Suppl 3)**:P185

**Background:** Benzylpenicilloyl-dendrimer conjugates are recognized by IgE specific to BP (benzylpenicillin). Dendrimers are monodisperse synthetic carriers, which chemical structure can be perfectly characterized. Their use compared to the conventional poly-l-lysine carrier improves the reproducibility and sensibility of in vitro tests to diagnose allergy. Moreover, increasing understanding of the immunological recognition of drug-carrier conjugates in vitro would help to improve diagnostic tests. Herein we include other penicillin epitope, AX (amoxicillin), and study Dendrimeric Antigen (DeAn) conjugates that contain two types of epitopes, BP and AX, in the same carrier dendrimer molecule. We hypothesize that the orientation and tridimensional disposition of the peripheral epitopes in the DeAn may play key roles in the IgE recognition.

**Materials and methods:** Synthesis of DeAn conjugates was accomplished by reaction of generation 4 PAMAM (PolyAMidoAMine) dendrimers with either one kind of penicillin (BP or AX) or both penicillins (BP and AX). Their chemical and tridimensional structures were studied by mono- and bi-dimensional NMR (Nuclear Magnetic Resonance) and MDS (Molecular Dynamic Simulation). The conjugates were immunologically evaluated by RAST (Radio-Allergo-Sorbent-Test) inhibition using sera from 6 patients allergic to penicillins (selective and cross-reactive).

**Results:** Spatial conformation showed differences between benzylpenicilloyl, with a hidden side chain, and amoxicilloyl, exposing the entire molecule outside the dendrimeric skeleton. Concerning immunological assays, at the maximum conjugate concentration, serum allergic to BP showed 80 % of RAST inhibition to both BP-DeAn and biepitope-DeAn, and negative to AX-DeAn. Sera allergic to AX showed 90 % of inhibition to both AX-DeAn and biepitope-DeAn, with no inhibition to BP-DeAn. All sera with cross-reactivity to both penicillins inhibited above 90 % to both AX-DeAn and biepitope-DeAn and above 60 % to BP-DeAn.

**Conclusions:** Experimental data show less recognition of benzylpenicilloyl compared to amoxicilloyl units in those biepitope-DeAn, probably due to the different accessibility of the side chain of both penicillins to IgE recognition. Therefore there is a correlation between the tridimensional chemical structures of penicillin epitopes and the way DeAn are recognized by sIgE. Moreover, the biepitope DeAn conjugates could represent the basis of a novel method for screening a wider proportion of allergic patients with a single test.

**Keywords:** Dendrimer; IgE; Penicillin

### P186 Dendrimeric antigens on solid supports: designed materials for IgE quantification

#### Yolanda Vida^1^, Maria Isabel Montañez^2^, Noemi Molina^1^, Daniel Collado^1^, Francisco Najera^1^, Adriana Ariza^3^, Maria Jose Torres^4^, Cristobalina Mayorga^2^, Ezequiel Perez-Inestrosa^1^

##### ^1^Universidad de Malaga-IBIMA, Department of Organic Chemistry. Andalusian Centre for Nanomedicine and Biotechnology-BIONAND, Malaga, Spain; ^2^Research Laboratory Carlos Haya Hospital-IBIMA. Andalusian Centre for Nanomedicine and Biotechnology-BIONAND, Malaga, Spain; ^3^Research Laboratory Carlos Haya Hospital-IBIMA., Malaga, Spain; ^4^Allergy Service, Carlos Haya Hospital, Malaga, Spain. Andalusian Centre for Nanomedicine and Biotechnology-BIONAND, Malaga, Spain

**Correspondence:** Yolanda Vida

*Clinical and Translational Allergy* 2016, **6(Suppl 3)**:P186

**Background:** Complex functional materials consisting of bioactive molecules immobilized on solid supports present potential applications in biosensoring. Advances in the fabrication of these surface materials are of growing interests in antibody-based diagnostic. We describe recent progress in the preparation of new materials for biosensor applications where Dendrimeric Antigens (DeAn), synthetic antigens where the role of the carrier protein is performed by a dendrimer, were supported on silica particles to assemble DeAn@SiO_2_ composites. DeAn@SiO_2_ containing the allergenic determinant to amoxicillin (AXO) has been studied for quantifying IgE specific to Amoxicillin.

**Materials and methods:** We report on the use of silica particles as a solid support for Radio-Allergo-Sorbent-Test (RAST) with a view to its use as a complementary diagnostic method for identifying allergic responses to penicillin. We prepared nanoconjugated Dendrimeric Antigens (DeAn) consisting of second-generation PAMAM dendrimers (PAMAM-G2) peripherally decorated with the suspected amoxicillin hapten (AXO). These organic–inorganic hybrid materials were carefully characterized and the preparation methodology was checked to be highly reproducible. To study the potential of those composites in the detection and quantification of antibodies, they were clinically tested by RAST using sera from allergic patients and control individuals tolerant to this drug, according to conventional protocols applied in the clinical practice.

**Results:** 0.4 mg of DeAn@SiO_2_ composites were used in each assay, producing positive RAST results (7 %) in sera from three allergic patients. No signal was obtained in the RAST on two control individuals (even though being one of them with diagnosed immediate allergic reaction to benzylpenicillin), ensuring a high specificity in the detection of antibodies. Additionally, larger amounts of composites (1 and 1.6 mg) were used in the assays, increasing the RAST level for patients allergic (14 and 18 % respectively), which successfully improves the sensitivity of the test. The percentage of RAST obtained with the control gave negatives values, maintaining the specificity of the test.

**Conclusions:** DeAn@SiO_2_ composites, containing the allergenic determinant to AXO, proved effective in detecting and quantifying IgE in sera from patients allergic to amoxicillin, in a specific and selective way. This new material is thus promising candidate for improving in vitro clinical diagnostic practice.

**Keywords:** IgE quantification; Dendrimers; Dendrimeric antigens

### P187 Development of a screening assay for drug hypersensitivity using naïve T cells from donors with seven different HLA class I risk alleles

#### Lee Faulkner, Sally Wood, Ana Alfirevic, Munir Pirmohamed, Dean J. Naisbitt, B. Kevin Park

##### University of Liverpool, Liverpool, United Kingdom

**Correspondence:** Lee Faulkner

*Clinical and Translational Allergy* 2016, **6(Suppl 3)**:P187

**Background:** A small proportion of adverse drug reactions (ADRs) are due to drug hypersensitivity where the immune response causes an unexpected and sometimes severe clinical reaction. Genome-wide screens have identified many different genetic associations between drugs and ADRs. Of particular interest to hypersensitivity reactions are the HLA Class I and Class II risk alleles. Not all individuals with a HLA risk allele will develop hypersensitivity. Thus, the frequency/severity of a reaction is a function of the chemistry of the drug, the biology of the immune system, the genotype of the individual and the underlying medical condition.

Drug hypersensitivity reactions are rare and are rarely detected during clinical trials. It is only once the drug is widely prescribed that these reactions become apparent. Since hypersensitivity reactions that target skin and liver are a major cause of drug withdrawal from the market, there is a need to develop a screening assay which could potentially detect these reactions during drug development.

**Materials and methods:** We have established a biobank of lymphocytes isolated from 1000 HLA-typed volunteers and have developed a DC priming assay which can prime naïve T cells to various drugs. The current form of the DC assay is labour intensive, requires large numbers of cells, uses different plate formats, has multiple readouts and takes 3–4 weeks to run. The aim is to modify this into a screening assay using a single plate with a single readout. We have selected up to 5 donors expressing seven different HLA Class I risk alleles: A*3101, A*3303, A*6801, B*1301, B*1502, B*5701 and B*5801. Ten donors without the HLA risk alleles will be included as negative controls and a unique donor exposed to nitroso-sulfamethoxazole (SMX-NO) will be used as a positive control.

**Results:** Initial development has concentrated on using the SMX-NO and 2 β-lactam antibiotics as positive controls to optimise the assay conditions, to assess the frequency of T-cell responses and to measure the strength of the response induced. Early results show that proliferative responses to SMX-NO have been detected in the screening assay at a similar intensity to that detected in the existing form of the assay. Further work will concentrate on reproducibility and inter-donor variation.

**Conclusions:** Once established, the screening assay will be used to investigate responses to ticlopidine, dapsone, carbamazepine, flucloxacillin allopurinol and abacavir.

### P188 Different patterns of recognition of structures derived from amoxicillin by IgE antibodies from patients with immediate hypersensitivity reactions to betalactams

#### Adriana Ariza^1^, Cristobalina Mayorga^1^, María Isabel Montañez^2^, María Salas^3^, Inmaculada Doña^3^, Ángela Martín-Serrano^2^, Ezequiel Pérez-Inestrosa^4^, Dolores Pérez-Sala^5^, Miguel Blanca^3^, Antonio E. Guzmán^6^, María José Torres^3^

##### ^1^Research Laboratory, IBIMA – Regional University Hospital of Malaga – University of Malaga, Málaga, Spain; ^2^Research Laboratory, IBIMA – Regional University Hospital of Malaga – University of Malaga; BIONAND, Málaga, Spain; ^3^Allergy Unit, IBIMA – Regional University Hospital of Malaga – University of Malaga, Málaga, Spain; ^4^Department of Organic Chemistry, IBIMA – University of Malaga; BIONAND, Málaga, Spain; ^5^Department of Chemical and Physical Biology, Centro de Investigaciones Biológicas, Madrid, Spain; ^6^Pharmacy Unit, Regional University Hospital of Malaga, Málaga, Spain

**Correspondence:** Adriana Ariza

*Clinical and Translational Allergy* 2016, **6(Suppl 3)**:P188

**Background:** Amoxicillin (AX) is the most frequent antibiotic elicitor of allergic reactions to drugs. It is widely accepted the requirement of the binding of the AX to a carrier molecule for its optimal IgE recognition. Although the carrier molecule seems to form part of the antigenic determinant, its contribution in the pattern of response is not fully known. The aim of this work was to study the recognition of different structures derived from AX by IgE antibodies from allergic patients to this drug.

**Materials and methods:** Patients (N = 21) with an immediate allergic reaction to AX and radioallergosorbent test (RAST) values >7 % were included. The IgE recognition study was performed by RAST inhibition using on the solid phase AX bound to poly-l-lysine and in the fluid phase the following inhibitors: AX, amoxicilloic acid, AX bound to butylamine (AXO-BA) and to human serum albumin (AXO-HSA), and fractions of different molecular weights of human serum modified with AX.

**Results:** Two well-defined patterns of recognition were observed: A group of patients in whom AX itself was significantly higher recognized (Group A, N = 8) and another group of patients who preferentially recognized AXO-BA and AXO-HSA (Group B, N = 13). No significant differences were observed for amoxicilloic acid, being significantly the lowest recognized structure in both groups. Regarding the pattern of recognition of different fractions from AX-modified human sera, groups A and B showed differences, with a higher recognition of the lowest molecular weight fraction (<3 kDa) in Group A and no differential recognition in Group B.

Interestingly, all patients classified as Group A had selective allergic reactions to AX, and all those in Group B had cross-reactive reactions.

**Conclusions:** Although both AX derivatives and carrier molecules are important for the IgE recognition, differences exist between patients with a selective or a cross-reacting response. In selective patients, it is necessary that the side chain of the AX was preserved in the AX-carrier adducts and exposed to the IgE, and this fact is influenced by the conditions in the formation of adducts. However, for cross-reactive patients, whose IgE antibodies recognize the AX side chain and part of the common structure of penicillins, the conditions for the generation of AX-carrier adducts seem not to be relevant for their recognition. The poor recognition of the amoxicilloic acid reinforces the need for binding the AX to a carrier molecule for an optimal recognition.

**Keywords:** Amoxicillin; IgE; Carrier molecule; Hypersensitivity

### P189 High-resolution typing of HLA polymorphism and T-cell receptor repertoire for severe adverse drug reactions based on the cost-effective next-generation sequencing approaches

#### Tai-Ming Ko, Yuan-Tsong Chen, Jer-Yuarn Wu

##### Academia Sinica, Taipei, Taiwan

**Correspondence:** Tai-Ming Ko

*Clinical and Translational Allergy* 2016, **6(Suppl 3)**:P189

**Background:** Immune-related severe adverse drug reaction (ADR) is widely hypothesised triggered by the combination of HLA, drugs, peptides, and T-cell receptor (TCR) molecules. Currently, numerous types of severe ADRs are reported to be related to particular HLA polymorphisms, such as HLA-B*15:02 allele associated with carbamazepine-induced Steven’s Johnson syndrome (SJS) and HLA-B*58:01 allele associated with allopurinol-induced severe cutaneous adverse reactions (SCARs). The use of TCR by drug-specific T cells is also considered a co-factor responsible for severe ADR. The major challenges of using Sanger sequencing, for high-throughput typing of HLA and TCR gene in large cohort studies, is the high cost involved. Moreover, it is unknown whether CBZ-SJS or allopurinol-SCARs is linked to more specified HLA types. Our aim was to identify which specified HLA types and TCR profiling correlated with CBZ-SJS or allopurinol-SCARs based on using cost-effective next-generation sequencing approaches.

**Materials and methods:** We enrolled patients with CBZ-SJS, CBZ-tolerant subjects, patients with allopurinol-SCARs, allopurinol-tolerant subjects, and healthy controls. For HLA typing, we developed an analysis pipeline for illumina Miseq or PacBio sequencing platform. For TCR typing, a widely used primer set was used and an analysis pipeline for illumina Miseq or Hiseq sequencing platform was developed. Sanger sequencing for the validation of HLA and TCR was performed.

**Results:** We genotyped all major HLA regions, including HLA-A, HLA-B, HLA-C, HLA-DPB1, HLA-DQB1, and HLA-DRB1, in all the subjects. Compared with sequence-based typing ($400 per sample) for HLA typing, the cost based on next-generation sequencing ($240 per sample) was reduced. CBZ-SJS patients or allopurinol-SCAR patients were HLA-B*15:02:01-positive or HLA-B*58:01:01-positive, respectively. For TCR, we genotyped all the V beta subfamilies. Dominant CDR3 sequences were verified by Sanger sequencing. Compared with Sanger sequencing ($500 per sample), the cost based on next-generation sequencing ($50 per sample) was much lower. Additionally, novel TCR clonotypes, possibly associated with SJS patients, were identified.

**Conclusions:** A cost-effective method for high-throughput and high-resolution typing of HLA and TCR was developed. HLA-B*15:02:01 and HLA-B*58:01:01 were found as more specified HLA genotypes for developing CBZ-SJS and allopurinol-SCARs, respectively.

### P190 Identification and fate of intracellular proteins haptenated by amoxicillin

#### Francisco J. Sánchez-Gómez^1^, Juan M. González-Morena^1^, Yolanda Vida^2^, Ezequiel Pérez-Inestrosa^2^, Miguel Blanca^3^, María J. Torres^3^, Dolores Pérez-Sala^1^

##### ^1^Department of Chemical and Physical Biology, Centro de Investigaciones Biológicas-CSIC, Madrid, Spain; ^2^Department of Organic Chemistry, University of Málaga, Málaga, Spain; ^3^Allergy Unit, IBIMA-Regional Hospital of Málaga, Málaga, Spain

**Correspondence:** Francisco J. Sánchez-Gómez

*Clinical and Translational Allergy* 2016, **6(Suppl 3)**:P190

**Background:** The common use of beta-lactams during the last decades is leading to an increase in the prevalence of allergic responses. In the allergic response, the adduct formation between small drugs and proteins, called haptenation, allows drugs to be recognized as antigens by the immune system. Moreover, it has been hypothesized that structures from target proteins can also contribute to the formation of the antigenic determinants. Identification of protein targets for haptenation is relevant for their potential use in novel diagnostic tools as well as for understanding the complete mechanisms involved in antigen presentation and the role played by the cells that participate in allergy induction. In recent years our lab has undertaken the identification of targets proteins for the haptenation by beta-lactams.

**Materials and methods:** We have attempted the identification of protein targets for covalent modification by amoxicillin (AX) and a biotinylated analogue (AX-B) as model compounds. To accomplish these aims, we decided to use a B-lymphocyte cell line as cellular model. The identification of the proteins haptenated by AX and AX-B has been approached with mass spectrometry experiments. In addition, intercellular communication mediated by vesicular delivery of molecular messages has been explored by microscopy techniques.

**Results:** In our experiments we have observed a complex pattern of proteins modified by amoxicillin. Among them, we have isolated and identified several novel targets for haptenation by AX in B-lymphocytes. In addition, these haptenated proteins have been observed both intracellularly and in the conditioned medium, where they were found both in soluble and vesicular fractions.

**Conclusions:** Our results indicate that intracellular proteins may be haptenated by beta-lactam antibiotics and reach the extracellular medium. The importance of these targets in the development of allergic reactions will be the subject of further investigations.

Funding: MINECOSAF2012-36519; ISCIII RD12/0013/0008

**Keywords:** Allergy; Amoxicillin; Haptenation; Exosomes; Intercellular communication

### P191 In vitro detection of terbinafine protein adducts

#### Arun Tailor, Toru Usui, Yanni Xue, Xiaoli Meng, Dean J. Naisbitt, B. Kevin Park

##### University of Liverpool, Liverpool, United Kingdom

**Correspondence:** Arun Tailor

*Clinical and Translational Allergy* 2016, **6(Suppl 3)**:P191

**Background:** Idiosyncratic drug induced liver injury (iDILI) is a severe adverse reaction which manifests in some, but not all drug recipients. The incidence of these reactions is so low that these adverse events cannot be attributed to the normal pharmacological action of a drug, but are probably due to the individual biology of a patient. To support this claim, an increasing number of genetic associations have been found in cases of iDILI, and many of these are with HLA alleles. This indicates that iDILI may have an immunological aetiology and there is increasing evidence to support this. For example: the detection of T-cells in liver biopsies from iDILI patients and the isolation of drug specific T-cells in patients suffering from flucloxacillin and co-amoxiclav induced liver injury. This sets a precedence to investigate other iDILI drugs such as terbinafine in the context of drug hypersensitivity reactions. Terbinafine can form a chemically reactive metabolite and like β-lactam antibiotics, may form drug-protein haptens to activate an immune response. Although the hapten model for drug hypersensitivity is well established, the precise nature of the drug antigen is unclear. The aim of this study is to investigate the formation of terbinafine adducts using a chemically reactive metabolite of terbinafine: allylic aldehyde, 7,7-dimethylhept-2-ene-4-ynal (TBF-A).

**Materials and methods:** TBF-A was incubated for 24 h at a 10:1 molar ratio for synthetic peptides and a 1:1 ratio for other molecules. Mass spectrometric analysis was conducted using an API 4000 or an QTRAP 5500 hybrid quadrupole-linear ion trap mass spectrometer (AB Sciex). Conjugates were detected using multiple reaction monitoring and also enhanced product ion scanning methods

**Results:** TBF-A showed binding to *N*-acetyl cysteine and glutathione but not to *N*-acetyl lysine. Additionally, TBF-A was shown to bind covalently to glutathione s-transferase pi. Following incubation with human serum albumin, no conjugates were detected. Synthetic cysteine containing peptides were also generated and shown to bind TBF-A.

**Conclusions:** TBF-A binds to cysteine residues in small molecules, larger proteins and also in synthetic peptides. Thus, we are now conducting additional studies to isolate TBF-A specific T-cells in order to investigate whether formation of this metabolite is important for the activation of the adaptive immune system in patients with iDILI.

### P192 MicroRNAs dysregulation in PBMCs from drug hypersensitivity patients during drug challenge in vitro

#### Alejandra Monroy Arreola^1^, Jesus Agustin Badillo Corona^1^, Silvia Mendez Flores^2^, Judith Dominguez Cherit^2^, Dean J. Naisbitt^3^, Noe Valentin Duran Figueroa^1^, Jose Luis Castrejon Flores^1^

##### ^1^Instituto Politecnico Nacional, Mexico, Mexico; ^2^Instituto Nacional de Ciencias Medicas y Nutricion, Mexico, Mexico; ^3^University of Liverpool, Liverpool, United Kingdom

**Correspondence:** Alejandra Monroy Arreola

*Clinical and Translational Allergy* 2016, **6(Suppl 3)**:P192

**Background:** Drug hypersensitivity reactions account for approximately 15 % of all adverse drug reactions, where the skin is the main organ affected generating an important health problem. During the last 20 years, we have gained insight into the immunological mechanism(s) behind different forms of cutaneous hypersensitivity, but our knowledge of post-transcriptional immune regulators remains poorly understood. MicroRNAs (miRNAs) play a key role in several immunological processes and in the pathogenesis of several diseases including severe skin conditions; nonetheless, their cellular origin and the changes in the expression as a result of drug exposure needs to be further investigated.

**Materials and methods:** Two allergic patients to sulfamethoxazole (SMX1 and SMX2) and one allergic patient to levofloxacin (LFV1) were enrolled in the study. Approval for the study was obtained from local research ethics committee, and informed written consent was obtained from each donor. The presence of drug specific T-cells was determined by LTT. Additionally, PMBCs (1.5 × 10^5^ cells/ml) from each patient were incubated by triplicate in cell culture alone or in the presence of the culprit drugs at different concentrations. The change in the pattern of expression of the miRNAs was compare between drug free cells and drug incubated cells at 0, 12, 24 and 48 h by RT-PCR using commercial specific probes for miR-18a, -21, -142 and -155. The U6 gene was used as reference, and the relative expression was calculated using the delta Cq method.

**Results:** Positive stimulation indexes were obtained from the three patients at different concentrations. Moreover, a significant increase in the expression of miR-18a, -21, -142 and -155 was detected after 12–48 h in the PBMCs of all the patients. Quantitative relative expression of the different miRNAs showed a fold change between 2 and 100 during the incubation time when compared with cells without drug. When a different drug, not related with the reaction, was used, the change in the pattern of expression was reduced or similar to basal levels.

**Conclusions:** Our findings clearly show that the incubation of the culprit drugs with PBMCs from hypersensitive patients modify the expression of miRNAs in a dose and time dependent manner. At the presence, we are evaluating the change in the pattern of expression of miRNAs in PBMCs of hypersensitive patients caused by anticonvulsants and in patients with severe cutaneous reactions (Table 11).

**Table 11:** Clinical information and functional assays results from three hypersensivity patients to antibiotics

Patient information				Lymphocyte transformation test	Change in the pattern of expresion of miRNAs with drugs			
Code	Sex	Age	Skin reaction	SI/Drug (μg/ml)	miRNA	Drug (μg/ml)	Relative expression	Time (h)
SMX1	F	60	MPE	5.0/SMX (200) 5.2/SMX-NO (20)	142	SMX (400)	153 ± 28	12
					18a	SMX-NO (12)	71.6 ± 1.21	24
					155	SMX-NO (12)	5.6 ± 1.1	24
SMX2	M	35	MPE	2.0/SMX (400) 3.0/SMX-NO (20)	18a	SMX (400)	2.5 ± 0.6	24
					21	SMX-NO (12)	6.3 ± 3.2	24
					142	SMX (400)	3.9 ± 0.7	24
LVF1	M	47	MPE	2.0/LVF (10)	142	LVF (10)	12.2 ± 0.9	24
					18a	LVF (10)	4.0 ± 0.4	48
					155	LVF (10)	5.1 ± 1.1	48

Relative expression results are presented as the Mean ± SD obtained by the delta Cq method

*SMX* sulfamethoxazole, *SMX*-*NO* sulfamethoxazole nitroso, *LVF* levofloxacin, *MPE* maculopapular exanthema

**Keywords:** MicroRNAs; Drug hypersensitivity; Mechanisms

### P193 NSAIDs-exacerbated cutaneous disease: high throughput gene expression profiling

#### José Antonio Cornejo-García^1^, James Perkins^2^, Natalia Blanca-López^3^, Diana Pérez-Alzate^3^, Raquel Jurado-Escobar^2^, Inmaculada Doña^4^, Gador Bogas^4^, María J. Torres^4^, Gabriela Canto^3^, Miguel Blanca^4^

##### ^1^Research Laboratory and Allergy Unit, IBIMA, Regional University Hospital of Malaga, UMA, Malaga, Spain; ^2^Research Laboratory, IBIMA, Regional University Hospital of Malaga, UMA, Malaga, Spain; ^3^Allergy Service, Infanta Leonor University Hospital, Madrid, Spain; ^4^Allergy Unit, IBIMA, Regional University Hospital of Malaga, UMA, Malaga, Spain

**Correspondence:** José Antonio Cornejo-García

*Clinical and Translational Allergy* 2016, **6(Suppl 3)**:P193

**Background:** Some patients with a history of chronic spontaneous urticaria may suffer from an exacerbation of this pathology after aspirin and other non-steroidal anti-inflammatory drugs (NSAIDs) intake, a condition termed NSAIDs-exacerbated cutaneous disease (NECD). In addition to the participation of COX-1 inhibition the underlying mechanism may be influenced by differential gene expression. To assess the genetic mechanisms potentially implicated in NECD we analysed gene expression patterns using microarrays technology.

**Materials and methods:** Total RNA was obtained during the acute phase from skin biopsies in 3 patients with NECD, and 14 heathy controls. After quality control evaluation, gene expression patterns were compared through the GeneChip Human Gene 2.0 ST microarrays system (Affymetrix).

Mechanism may be influenced by differential gene expression. To assess the genetic mechanisms potentially implicated in NECD we analysed gene expression patterns using microarrays technology.

R**esults:** We found 163 differentially expressed transcripts in NECD patients from a total of 48,227 analysed (FDR p value <0.05, and a log fold change >1 or <1). Top upregulated transcripts were related to the structural integrity of epithelial cells (*KRT16, KRT17*), the HLA system (*HLA*-*DQB1*) and microRNAs (*MIR4427*). Some downregulated transcripts were also related to microRNAs (*MIR3127*) and microRNA-mediated gene repression (*DND1*), adhesion and migration of epithelial cells (*POSTN*), transcription factors (*ZNF667*), and collagen integrity (*COL6A5*).

**Conclusions:** We described a differential expression pattern in NECD suggesting intricate interactions in gene regulation affecting skin structural integrity, cell adhesion and migration, and the HLA system. Our results shed new light for understanding the mechanisms underlying this entity: (normal’ > KRT16, KRT17), the HLA system (*HLA*-*DQB1*) and microRNAs (*MIR4427*). Some downregulated transcripts were also related to microRNAs (*MIR3127*) and microRNA-mediated gene repression (*DND1*), adhesion and migration of epithelial cells (*POSTN*), transcription factors (*ZNF667*), and collagen integrity (*COL6A5*).

**Keywords:** Drug hypersensitivity; NSAIDs; NECD; Gene expression

### P194 Utility of skin tests in non-immediate reactions to amoxicillin

#### Luis Mario Tubella Marti^1^, Fernando Pineda De La Losa^2^, Francisca Arribas Poves^2^, Jaime Tubella Lopez^1^, Teodora Lopez Santiago^1^

##### ^1^Alergology Service. Hospital Delfos., Barcelona, Spain; ^2^DIATER Laboratorios, Madrid, Spain

**Correspondence:** Luis Mario Tubella Marti

*Clinical and Translational Allergy* 2016, **6(Suppl 3)**:P194

**Background:** Allergy to beta-lactam antibiotics, specifically penicillins, is presented differently depending on the time spent from administration of the drug until the reaction. Thus we can classify these manifestations as immediate, accelerated or delayed, or simply as immediate or not immediate.

**Materials and methods:** Patient who comes to be attended after having suffered 2 episodes of RAM after taking amoxicillin. Both episodes coursed with generalized skin rash, 8 days after taking amoxicillin 500 per a picture of tonsillitis and the second 5 days after taking amoxicillin 500 per a tooth extraction. On both occasions the picture is manifested after a latency period of 48–72 h after the last intake. Skin tests with amoxicillin (DAP DIATER kit) by prick test and ID to 1:10 and 1:100 and undiluted. Oral challenge test against Cefuroxime increasing doses up to the maximum cumulative dose of 500 mg. RAST tests against penicilloyl G and V, ampicillin, amoxicillin and cefaclor.

**Results:** The skin tests were positive for amoxicillin ID undiluted, 1:10 and 1:100 at 72 h. The oral food challenge to cefuroxime at the doses administered was negative. RAST tests against penicilloyl G and V, Ampicillin, Amoxicillin and negative cefaclor.

**Conclusions:** We report a case of delayed reaction to amoxicillin with tolerance to cefuroxime, where the history and skin tests were essential in the diagnosing of this case.

**Keywords:** Diagnostic; Skin test; Amoxicillin; Non-immediated

